# Clinical risk factors of adverse outcomes among women with COVID-19 in the pregnancy and postpartum period: a sequential, prospective meta-analysis

**DOI:** 10.1016/j.ajog.2022.08.038

**Published:** 2023-02

**Authors:** Emily R. Smith, Erin Oakley, Gargi Wable Grandner, Gordon Rukundo, Fouzia Farooq, Kacey Ferguson, Sasha Baumann, Kristina Maria Adams Waldorf, Yalda Afshar, Mia Ahlberg, Homa Ahmadzia, Victor Akelo, Grace Aldrovandi, Elisa Bevilacqua, Nabal Bracero, Justin S. Brandt, Natalie Broutet, Jorge Carrillo, Jeanne Conry, Erich Cosmi, Fatima Crispi, Francesca Crovetto, Maria del Mar Gil, Camille Delgado-López, Hema Divakar, Amanda J. Driscoll, Guillaume Favre, Irene Fernandez Buhigas, Valerie Flaherman, Christopher Gale, Christine L. Godwin, Sami Gottlieb, Eduard Gratacós, Siran He, Olivia Hernandez, Stephanie Jones, Sheetal Joshi, Erkan Kalafat, Sammy Khagayi, Marian Knight, Karen L. Kotloff, Antonio Lanzone, Valentina Laurita Longo, Kirsty Le Doare, Christoph Lees, Ethan Litman, Erica M. Lokken, Shabir A. Madhi, Laura A. Magee, Raigam Jafet Martinez-Portilla, Torri D. Metz, Emily S. Miller, Deborah Money, Sakita Moungmaithong, Edward Mullins, Jean B. Nachega, Marta C. Nunes, Dickens Onyango, Alice Panchaud, Liona C. Poon, Daniel Raiten, Lesley Regan, Daljit Sahota, Allie Sakowicz, Jose Sanin-Blair, Olof Stephansson, Marleen Temmerman, Anna Thorson, Soe Soe Thwin, Beth A. Tippett Barr, Jorge E. Tolosa, Niyazi Tug, Miguel Valencia-Prado, Silvia Visentin, Peter von Dadelszen, Clare Whitehead, Mollie Wood, Huixia Yang, Rebecca Zavala, James M. Tielsch

**Affiliations:** a Department of Global Health, Milken Institute School of Public Health, George Washington University, Washington, DC; b PeriCOVID (PREPARE)–Uganda Team, Makerere University–Johns Hopkins University Research Collaboration, Kampala, Uganda; c Department of Obstetrics and Gynecology, University of Washington School of Medicine, Seattle, WA; d Department of Global Health, University of Washington, Seattle, WA; e Division of Maternal-Fetal Medicine, University of California, Los Angeles, Los Angeles, CA; f Division of Division of Clinical Epidemiology, Department of Medicine, Solna, Karolinska Institutet, Stockholm, Sweden; g Division of Maternal-Fetal Medicine, The George Washington University School of Medicine and Health Sciences, Washington, DC; h Centers for Disease Control and Prevention, Kisumu, Kenya; i Department of Pediatrics, University of California, Los Angeles, Los Angeles, CA; j Department of Women and Child Health, Women Health Area, Fondazione Policlinico Universitario Agostino Gemelli, Istituto di Ricovero e Cura a Carattere Scientifico, Rome, Italy; k Department of Obstetrics and Gynecology, University of Puerto Rico School of Medicine, San Juan, PR; l Puerto Rico Obstetrics and Gynecology (PROGyn); m Division of Maternal-Fetal Medicine, Department of Obstetrics, Gynecology and Reproductive Sciences, Rutgers Robert Wood Johnson Medical School, New Brunswick, NJ; n Department of Sexual and Reproductive Health and Research, World Health Organization, Geneva, Switzerland; o Departamento de Obstetricia y Ginecologia, Clinica Alemana de Santiago, Facultad de Medicina Clinica Alemana-Universidad del Desarrollo, Santiago, Chile; p International Federation of Gynecology and Obstetrics, London, United Kingdom; q Department of Women’s and Children’s Health, Obstetrics and Gynecology Clinic, University of Padua, Padua, Italy; r BCNatal, Barcelona Center for Maternal-Fetal and Neonatal Medicine, Hospital Sant Joan de Déu Barcelona and Hospital Clínic de Barcelona, Universitat de Barcelona, and Center for Biomedical Research on Rare Diseases, Barcelona, Spain; s Department of Obstetrics and Gynecology, Hospital Universitario de Torrejón, Madrid, Spain; t School of Medicine, Universidad Francisco de Vitoria, Madrid, Spain; u Surveillance for Emerging Threats to Mothers and Babies, Puerto Rico Department of Health, San Juan, PR; v Asian Research & Training Institute for Skill Transfer, Bengaluru, India; w Center for Vaccine Development and Global Health, University of Maryland School of Medicine, Baltimore, MD; x Materno-fetal and Obstetrics Research Unit, Département Femme-Mère-Enfant, Lausanne University Hospital, Lausanne, Switzerland; y Department of Pediatrics, University of California, San Francisco, San Francisco, CA; z Neonatal Medicine, School of Public Health, Faculty of Medicine, Imperial College London, London, United Kingdom; aa Gynecology and Obstetrics, Félix Bulnes Hospital and RedSalud Clinic, Santiago, Chile; ab South African Medical Research Council, Vaccines and Infectious Diseases Analytics Research Unit and Department of Science and Technology/National Research Foundation, South African Research Chair Initiative in Vaccine Preventable Diseases, Faculty of Health Sciences, University of the Witwatersrand, Johannesburg, South Africa; ac Department of Obstetrics and Gynecology, School of Medicine, Koç University, Istanbul, Turkey; ad Kenya Medical Research Institute–Centre for Global Health Research, Kisumu, Kenya; ae National Perinatal Epidemiology Unit, Nuffield Department of Population Health, University of Oxford, Oxford, United Kingdom; af Catholic University of Sacred Heart, Rome, Italy; ag Medical Research Council /Uganda Virus Research Institute and London School of Hygiene & Tropical Medicine Uganda Research Unit, Entebbe, Uganda; ah Paediatric Infectious Disease Research Group, St George’s University of London, London, United Kingdom; ai Department of Metabolism, Digestion and Reproduction, Imperial College London, London, United Kingdom; aj Department of Women and Children’s Health, School of Life Course Sciences, King’s College London, London, United Kingdom; ak Institute of Women and Children’s Health, King’s College Hospital, London, United Kingdom; al Clinical Research Branch, National Institute of Perinatology, Mexico City, Mexico; am Division of Maternal-Fetal Medicine, The University of Utah Health, Salt Lake City, UT; an Division of Maternal-Fetal Medicine, Department of Obstetrics and Gynecology, Northwestern University Feinberg School of Medicine, Chicago, IL; ao Department of Obstetrics and Gynecology, The University of British Columbia, Vancouver, Canada; ap Department of Obstetrics and Gynecology, The Chinese University of Hong Kong, Hong Kong; aq George Institute for Global Health, London, United Kingdom; ar Department of Epidemiology and Center for Global Health, University of Pittsburgh Graduate School of Public Health, Pittsburgh, PA; as Department of Medicine, Faculty of Medicine and Health Sciences, Stellenbosch University, Cape Town, South Africa; at Departments of Epidemiology and International Health, Johns Hopkins Bloomberg School of Public Health, Baltimore, MD; au Kisumu County Department of Health, Kisumu, Kenya; av Institute of Primary Health Care (BIHAM), University of Bern, Bern, Switzerland; aw Service of Pharmacy, Lausanne University Hospital, University of Lausanne, Lausanne, Switzerland; ax Pediatric Growth and Nutrition Branch, *Eunice Kennedy Shriver* National Institute of Child Health and Human Development, National Institutes of Health, Bethesda, MD; ay International Federation of Gynecology and Obstetrics, Imperial College London, London, United Kingdom; az Maternal-Fetal Unit, Universidad Pontificia Bolivariana, Medellín, Colombia; aaa Centre of Excellence in Women and Child Health, Aga Khan University, Nairobi, Kenya; aab Nyanja Health Research Institute, Salima, Malawi; aac Department of Obstetrics and Gynecology, Maternal Fetal Medicine, Oregon Health & Science University, Portland, OR; aad Department of Obstetrics and Gynecology, Maternal Fetal Medicine, St. Luke’s University Health Network, Bethlehem, PA; aae Department of Obstetrics and Gynecology, Sancaktepe Sehit Prof. Dr. Ilhan Varank Training and Research Hospital, Istanbul, Turkey; aaf Division of Children with Special Medical Needs, Puerto Rico Department of Health, San Juan, PR; aag Global Health Institute, King’s College London, London, United Kingdom; aah Department of Maternal Fetal Medicine, University of Melbourne, Royal Women’s Hospital, Melbourne, Australia; aai Department of Epidemiology, Gillings School of Global Public Health, The University of North Carolina at Chapel Hill, Chapel Hill, NC; aaj Department of Obstetrics and Gynecology, Peking University First Hospital, Beijing, China

**Keywords:** COVID-2019, maternal mortality, neonatal mortality, pneumonia, pregnancy, preterm birth, SARS-CoV-2, small-for-gestational-age

## Abstract

**Objective:**

This sequential, prospective meta-analysis sought to identify risk factors among pregnant and postpartum women with COVID-19 for adverse outcomes related to disease severity, maternal morbidities, neonatal mortality and morbidity, and adverse birth outcomes.

**Data Sources:**

We prospectively invited study investigators to join the sequential, prospective meta-analysis via professional research networks beginning in March 2020.

**Study Eligibility Criteria:**

Eligible studies included those recruiting at least 25 consecutive cases of COVID-19 in pregnancy within a defined catchment area.

**Methods:**

We included individual patient data from 21 participating studies. Data quality was assessed, and harmonized variables for risk factors and outcomes were constructed. Duplicate cases were removed. Pooled estimates for the absolute and relative risk of adverse outcomes comparing those with and without each risk factor were generated using a 2-stage meta-analysis.

**Results:**

We collected data from 33 countries and territories, including 21,977 cases of SARS-CoV-2 infection in pregnancy or postpartum. We found that women with comorbidities (preexisting diabetes mellitus, hypertension, cardiovascular disease) vs those without were at higher risk for COVID-19 severity and adverse pregnancy outcomes (fetal death, preterm birth, low birthweight). Participants with COVID-19 and HIV were 1.74 times (95% confidence interval, 1.12–2.71) more likely to be admitted to the intensive care unit. Pregnant women who were underweight before pregnancy were at higher risk of intensive care unit admission (relative risk, 5.53; 95% confidence interval, 2.27–13.44), ventilation (relative risk, 9.36; 95% confidence interval, 3.87–22.63), and pregnancy-related death (relative risk, 14.10; 95% confidence interval, 2.83–70.36). Prepregnancy obesity was also a risk factor for severe COVID-19 outcomes including intensive care unit admission (relative risk, 1.81; 95% confidence interval, 1.26–2.60), ventilation (relative risk, 2.05; 95% confidence interval, 1.20–3.51), any critical care (relative risk, 1.89; 95% confidence interval, 1.28–2.77), and pneumonia (relative risk, 1.66; 95% confidence interval, 1.18–2.33). Anemic pregnant women with COVID-19 also had increased risk of intensive care unit admission (relative risk, 1.63; 95% confidence interval, 1.25–2.11) and death (relative risk, 2.36; 95% confidence interval, 1.15–4.81).

**Conclusion:**

We found that pregnant women with comorbidities including diabetes mellitus, hypertension, and cardiovascular disease were at increased risk for severe COVID-19–related outcomes, maternal morbidities, and adverse birth outcomes. We also identified several less commonly known risk factors, including HIV infection, prepregnancy underweight, and anemia. Although pregnant women are already considered a high-risk population, special priority for prevention and treatment should be given to pregnant women with these additional risk factors.

**AJOG at a Glance**

**Why was this study conducted?**

Pregnant women are at risk for severe SARS-CoV-2 complications, and those with comorbidities might be at even higher risk for adverse outcomes. Furthermore, some vaccines and treatments are only recommended for those at highest risk. There is no global consensus about what risk factors signify such risk. Heterogeneity in the design and analysis of published studies and limited global data further complicate definitive guidance.

**Key findings**

We pooled individual patient data from 21 studies (33 countries, 21,977 pregnancies) and found that comorbidities, nutritional status, and older maternal age were associated with severe COVID-19–related outcomes (intensive care unit admission, ventilation, mortality), adverse pregnancy outcomes, and fetal/neonatal morbidity and mortality.

**What does this add to what is known?**

We pooled and reanalyzed data from global collaborators. We assessed high-priority risk factors and 24 consistently defined maternal and newborn outcomes. Given the large sample, including data from low- and middle-income countries, we generated estimates on rare outcomes (maternal mortality, stillbirth) and risk factors (anemia, underweight, HIV) where data have been lacking.

## Introduction

Since the onset of the novel COVID-19 pandemic, the World Health Organization (WHO) and the Centers for Disease Control and Prevention classified pregnant women as a group at higher risk of severe complications from SARS-CoV-2 infection compared with nonpregnant people.^1^^,^^2^ Despite known risk, pregnant women have been widely excluded from pharmaceutical clinical trials, resulting in an underdocumentation of the physiology, case count, complications, and consequences of COVID-19 in pregnancy.

Initial evidence showed that SARS-CoV-2 infection during pregnancy is linked to increased likelihood of adverse maternal, fetal, and neonatal outcomes.^3^, ^4^, ^5^ A systematic review of 42 studies (N=438,548) found that pregnant women with SARS-CoV-2 infection had significantly higher odds of preeclampsia, preterm birth, stillbirth, and intensive care unit (ICU) admission compared with those not infected.^5^ Although vertical transmission of COVID-19 from mother to fetus reportedly occurs in a low percentage of cases, neonates can be negatively affected by maternal infection in other ways.^6^^,^^7^ In 2 systematic reviews of 42 and 66 studies, neonates of mothers with confirmed COVID-19 had 3 times higher odds of neonatal ICU (NICU) admission than those born to uninfected mothers.^5^^,^^6^

Among pregnant women, multiple risk factors for severe SARS-CoV-2 infection have been identified.^3^^,^^8^ The Surveillance for Emerging Threats to Mothers and Babies Network in the United States (N=7950) determined that pregnant women aged >25 years with prepregnancy obesity, chronic lung disease, chronic hypertension, and pregestational diabetes mellitus had a 32% to 85% increased risk of moderate-to-severe COVID-19 compared with pregnant women without these conditions.^9^ Pregnant women with ≥3 underlying health conditions had over twice the risk of moderate-to-severe COVID-19 illness compared with those with no comorbidities.^9^

In the general population, nutritional status has been introduced as a potential risk factor for severe COVID-19. A meta-analysis of 7 studies (N=9912) found that among people with COVID-19, those with anemia had 2.44 higher odds of severe illness compared with nonanemic people.^10^ A scientific review found that sufficient intake of micronutrients, proteins, dietary fiber, short-chain fatty acids, and omega-3 polyunsaturated fatty acids may act as a protective factor against severe illness in COVID-19 patients.^11^ Further research is required for pregnant women, for whom nutritional guidance would be particularly useful.

There is an urgent need to pool high-quality and internationally representative data assessing the underlying risk factors and outcomes linked to COVID-19 in pregnancy. Currently, scarcity of similarly collected and analyzed data hampers our ability to make strong recommendations for the introduction and prioritization of new pharmaceutical interventions in pregnancy. The primary aim of this sequential, prospective meta-analysis (sPMA) is to accrue harmonized global data to inform policy and practice, grounded in the epidemiology of COVID-19 in the pregnancy, peripartum, and postnatal periods.

## Objectives

This study aimed to identify risk factors among pregnant and postpartum women with SARS-CoV-2 infection for adverse outcomes related to: (1) disease severity; (2) maternal morbidities; (3) fetal and neonatal mortality and morbidity; and (4) adverse birth outcomes.

## Methods

We registered the protocol for this prospective meta-analysis via the International Prospective Register of Systematic Reviews (PROSPERO) (ID: 188955) in May 2020, and the full protocol has been published elsewhere.^12^ The meta-analysis project was determined to be exempt from institutional review board review.

## Language

Not all of those who are pregnant or give birth identify as women; throughout this document, the term “pregnant women” should be taken to be inclusive of all persons who have the biological capability to carry a pregnancy regardless of gender identity.

## Eligibility criteria

Eligible studies include registries and single- or multisite cohort studies that recruited pregnant and recently postpartum women with confirmed or suspected COVID-19. They must have enrolled at least 25 women within a defined catchment area. We included data from those with infection onset up to 42 days after the pregnancy outcome.

## Study selection

We invited principal investigators of studies of COVID-19 in pregnancy to join the sPMA via professional research networks and collaborations with key stakeholder networks.

## Data extraction and individual patient data integrity

Following identification of eligible studies, investigators shared individual patient data (IPD) with the technical team for review and analysis. The technical team processed data to review data quality, identify outliers, and reconstruct variables to align with harmonized definitions of outcomes as defined in our protocol. We shared results with investigators for review and approval. For study sites unable to share IPD directly, the technical team worked with investigators to implement a common set of Stata codes to complete the same process of review, data quality checks, and harmonization.

In cases where studies collected data from overlapping catchment areas, we worked with investigators to identify and remove potential duplicates from the analysis. Because of the harmonization process and removal of overlapping data, there are some differences between our study results and those of the original published studies; these differences are summarized in Table S1.

## Assessment of risk of bias

We used an adapted Newcastle–Ottawa scale to review study quality and risk of bias for each participating study; criteria for determination of high or low risk for each study design element are presented in Table S2.^13^

## Outcomes

We examined 24 outcomes related to: (1) COVID-19 severity; (2) maternal morbidities; (3) fetal and neonatal morbidity and mortality; and (4) adverse birth outcomes. Specific definitions of each outcome—and 4 alternative outcomes used in sensitivity analyses—are presented in Table S3. The definition of maternal, fetal, and neonatal death and adverse birth outcomes were based on WHO case definitions.^14^, ^15^, ^16^, ^17^ Individual study sites defined hospitalization, critical care, and maternal morbidity outcomes. For maternal morbidities, fetal and neonatal mortality, and all birth outcomes, we restricted to cases of COVID-19 with infection onset during pregnancy or within 7 days of pregnancy outcome, excluding postpartum cases with COVID-19 onset at 8 to 42 days postpartum. Cases with unknown gestational age at onset were included in the analysis of pregnancy-specific outcomes and were assumed to be infections during pregnancy on the basis of the study design.

## Risk factors

The sPMA steering committee, on the basis of expert opinion, identified 9 high-priority maternal risk factors including comorbidities, nutritional status, age, parity, and COVID-19 symptomatic status. Comorbidities included preexisting diabetes mellitus, hypertension, cardiovascular disease, and HIV coinfection.

Nutrition-related risk factors included body mass index (BMI) and anemia. We relied on prepregnancy BMI to determine the category for each participant, and we examined 2 risk factors: underweight (BMI <18.5 kg/m^2^) and obesity (BMI ≥30 kg/m^2^). Both risk factors were compared with a reference group of participants who were of normal weight or overweight before pregnancy (BMI, 18.5 to <30 kg/m^2^). Anemia was diagnosed on the basis of a hemoglobin measurement <11 g/dL at the time of COVID-19 diagnosis.

We considered 2 age groups as risk factors: younger maternal age (15–19 years) and older maternal age (35–45 years). Both groups were compared with a reference group of women aged 20 to 34 years. Lastly, we considered being symptomatic for COVID-19, as opposed to having no symptoms, as a risk factor for the outcomes of interest.

## Generating study-specific estimates

We used a standard set of analysis codes to calculate study-specific estimates, comparing those with and without each risk factor (proportions and relative risks [RRs] with 95% confidence intervals [CIs]) for each participating study. Within each study, individual participants were excluded from the analysis if they were missing data on the risk factor of interest. Any study missing >25% of the data on an outcome of interest was excluded from that specific analysis.

## Data synthesis

We applied a 2-stage IPD meta-analytic framework to generate pooled absolute risks and RRs, with 95% CI for each risk factor–outcome pair when there were ≥3 studies with available data. We presented unadjusted estimates because the goal of this study was to present descriptive epidemiologic data among a group of people (pregnant women with COVID-19 and their infants), rather than to examine a causal relationship.^18^^,^^19^ To estimate the pooled absolute risk for each adverse outcome overall and within risk factor groups, we used a logistic-normal random-effects model.^20^ In cases where the logistic-normal model did not converge, we used a random-effects model with the Freeman–Tukey double arcsine transformation to ensure stable estimates and approximate asymptotic normality.^21^ We used a DerSimonian and Laird random-effects meta-analysis to generate RRs for each risk factor–outcome pair and assessed heterogeneity across studies using the I^2^ statistic.

We excluded studies with 0 total events from that particular analysis. In case of 0 events within a risk factor subgroup, we applied a continuity correction of 0.5 when calculating pooled absolute risks. For pooled RRs, we applied a continuity correction of the inverse number of events in the opposite group within the same study for the risk factor–outcome pair. All meta-analyses were conducted in Stata, version 16.1 (StataCorp LLC, College Station, TX).

## Results

### Study selection

**Figure 1:**
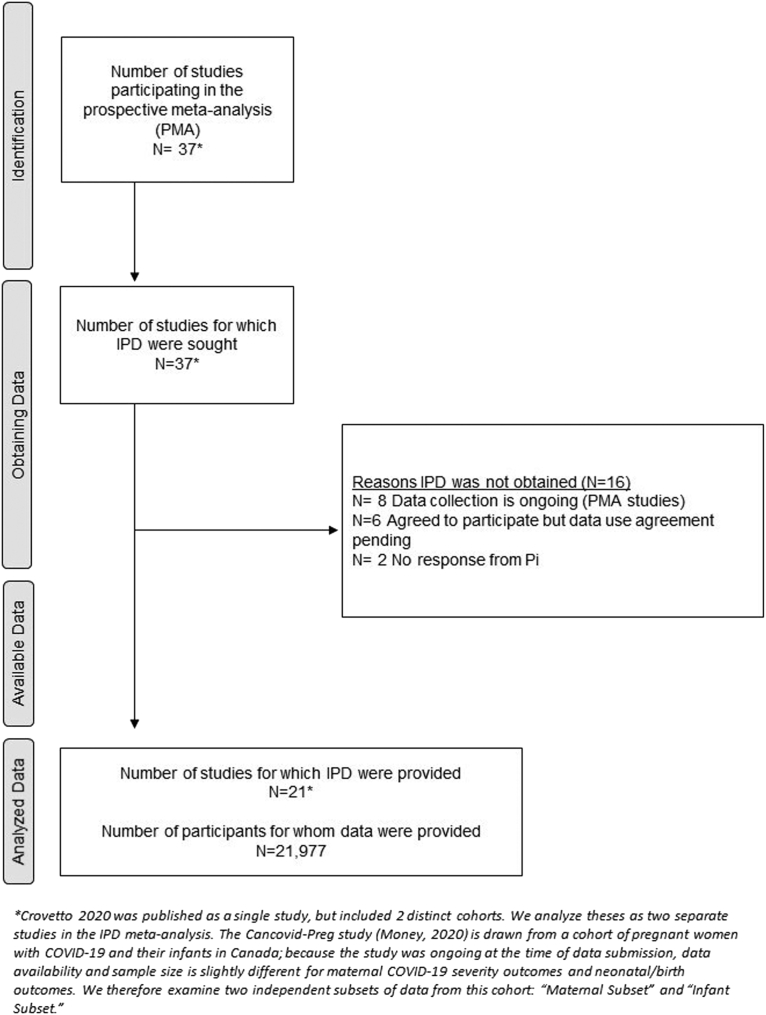
PRISMA diagram for risk factor analysis study The PRISMA flow diagram outlines the identification and recruitment of studies and final inclusion of individual patient data for this study. PRISMA, Preferred Reporting Items for Systematic Reviews and Meta-Analyses. *Smith. Individual patient data meta-analysis: risk factors among COVID-19 pregnancies. Am J Obstet Gynecol 2023.*

We included data from 21 studies conducted across 33 countries and territories (Afghanistan, Albania, Argentina, Belgium, Brazil, Canada, Chile, China, Colombia, Democratic Republic of the Congo, Egypt, France, French Guiana, Germany, Ghana, Hong Kong [China], India, Indonesia, Ireland, Israel, Italy, Kenya, Mexico, Nigeria, Portugal, PR, South Africa, Spain, Switzerland, Turkey, Uganda, United Kingdom, United States) with data from 21,977 cases of confirmed or suspected SARS-CoV-2 infections in pregnancy or the postpartum period. This iteration of the analysis included data from any study that met eligibility criteria and was able to share data by December 2021 (Figure 1). One study (Crovetto et al^22^) included 2 distinct cohorts with separate recruitment strategies, which were considered separately throughout the analysis. Furthermore, the Canadian Surveillance of COVID-19 in Pregnancy (CANCOVID-Preg) study (Money^23^) followed a cohort of pregnant women with SARS-CoV-2 infection and their infants in Canada; because the study was ongoing at the time of data submission, risk factor data availability and sample size were slightly different for maternal COVID-19 severity outcomes (n=2045) and neonatal/birth outcomes (n=2626). Therefore, we present the outcomes from the CANCOVID-Preg study as 2 independent subsets of the cohort in our tables (see CANCOVID-Preg–Maternal Subset and CANCOVID-Preg–Infant Subset).

#### Study characteristics

Cases occurred between January 2020 and December 2021 (Table 1). More than 11,000 cases were contributed by the Mexico National Registry (R.J.M.P.), accounting for approximately half of the data for COVID-19 severity outcomes. The other 20 studies contributed 10,946 pregnant patients and completed follow-up through the end of pregnancy for 9850 participants, including 9695 live births (Table 1).

**Table 1:** Description of studies contributing to the individual patient data meta-analysis

					Gestational age at infection							
Study PI	Countries	Total pregnancies	Livebirths	Mean age (SD)	1st tri	2nd tri	3rd tri	Postpartum	Unknown	Hospitalized (%)	Admitted to ICU (%)	Data collected through
Martinez-Portilla et al, 2020^46^	Mexico	11,031	n/a	28.5 (6.0)	n/a	n/a	n/a	n/a	100%	20%	2%	March 2021
Vouga et al, 2021^47^	14 countries^a^	2391	1830	31.3 (5.4)	10%	20%	37%	5%	29%	22%^b^	4%	Dec. 2021
McClymont et al, 2022^23^^c^	Canada	2045	—	31.2 (5.4)	7%	28%	49%	0%	16%	n/a	2%	Sept. 2021
McClymont et al, 2022^23^^c^	Canada^d^	—	2626	—	2%	7%	19%	0%	72%	n/a	n/a	Sept. 2021
Hernández et al, 2020^48^	Chile	1347	1113	29 (6.2)	1%	12%	64%	4%	19%	16%	6%	Nov. 2020
Knight et al, 2020^49^	United Kingdom	1243	1034	31.0 (6.0)	3%	12%	75%	4%	7%	100%^e^	6%	Oct. 2020
Bracero, Valencia, Delgado-Lopez (Unpublished data, October 20, 2021)	PR (United States)	938	744	26.6 (5.6)	11%	20%	38%	1%	30%	n/a	n/a	Oct. 2021
Sakowicz et al, 2020^50^	United States (Chicago, IL)	503	509	30.8 (5.8)	5%	21%	73%	0%	1%	n/a	1%	Feb. 2021
Sanin, Mesa, Tolosa (Unpublished data, June 28, 2021)	Colombia	409	188	n/a	4%	9%	32%	3%	52%	68%	22%	March 2021
Nachega et al, 2022^51^	DRC, Ghana, Kenya, Nigeria, South Africa, Uganda	349	136	30.7 (5.8)	6%^f^	18%^f^	64%^f^	0%^f^	12%^f^	100%	19%	Dec. 2020
Lokken et al, 2021^52^	United States (Washington, DC)	240	156	28.6 (5.8)	16%	28%	56%	0%	0%	10%	3%	Sept. 2020
Divakar (Unpublished data, February 8, 2021)	India (Karnataka)	214	216	26.4 (4.2)	0%	2%	82%	15%	0%	n/a	n/a	Dec. 2020
Gil, Fernandez Buhigas (Unpublished data, May 4, 2021)	Spain (Madrid)	212	168	32.6 (5.9)	29%	37%	33%	0%	1%	4%	0%	May 2021
Crovetto et al, 2021, Cohort II^22^	Spain (Barcelona)	176	178	32.0 (6.2)	n/a	n/a	14.%^g^	1%^g^	86%^g^	16%	1%	May 2020
Crovetto et al, 2021, Cohort I^22^	Spain (Barcelona)	173	154	32.7 (5.4)	n/a	n/a	n/a	n/a	100%^g^	0%	0%	March–May 2020, with follow-up through labor and delivery
Bevilacqua, Laurita Longo (Unpublished data, May 5, 2021)	Italy (Rome)	163	156	32.3 (5.4)	6%	5%	88%	0%	2%	7%	1%	March 2021
Nunes (Unpublished data, September 29, 2021)	South Africa	139	137	31.8 (6.6)	2%	22%	71%	0%	5%	15%	n/a	Sept. 2020
Akelo, Tippett Barr (Unpublished data, August 19, 2021)	Kenya	125	94	26.3 (5.2)	1%	12%	31%	27%	29%	9%	n/a	Aug. 2021
Yang, Juan (Unpublished data, October 26, 2020)	China	116	100	30.8 (3.8)	3%	6%	82%	9%	1%	100%	8%	March 2020
Kalafat et al, 2020^53^	Turkey	77	72	28.0 (5.9)	n/a	n/a	n/a	n/a	100%	75%	1%	June 2020
Brandt et al, 2021^54^	United States (New Brunswick)	61	60	30.3 (6.4)	0%	5%	90%	5%	0%	7%	2%	June 2020
Poon (Unpublished data, September 29, 2021)	Hong Kong	25	24	33.7 (5.4)	4%	28%	64%	0%	4%	92%	4%	June 2021

*DRC*, Democratic Republic of the Congo; *ICU*, intensive care unit; *n/a*, not applicable; *PI*, principal investigator(s); *SD*, standard deviation; *tri*, trimester; *USA*, United States of America.

*Smith. Individual patient data meta-analysis: risk factors among COVID-19 pregnancies. Am J Obstet Gynecol 2023.*

a The COVI-Preg study estimates in this analysis are drawn from facilities in 14 countries: Afghanistan (1%), Albania (<1%), Argentina (2%), Belgium (1%), Brazil (7%), Egypt (<1%), France (22%), French Guiana (3%), Germany (1%), Indonesia (1%), Ireland (2%), Israel (9%), Portugal (5%), and Switzerland (45%). Facilities participating in the COVI-Preg study with the potential to record overlapping cases with other sites participating in the current analysis were excluded, including facilities in Chile, China, Colombia, Italy, Spain (Barcelona), Mexico, Canada, United Kingdom, and the United States

b Hospitalization data were missing in the COVI-Preg study for 194 participants (8% of the sample). ICU admission data are only available from those with a recorded hospital admission

c The CANCOVID-Preg study follows a cohort of pregnant women with SARS-CoV-2 infection and their infants; because the study was ongoing at the time of data submission, risk factor data availability and sample size are slightly different for maternal COVID-19 severity outcomes and neonatal/birth outcomes. We present the data as 2 subsets of the same cohort for this ongoing study. In the “Maternal Subset,” we present data on pregnant women with COVID-19, including outcomes on ICU admission, ventilation, and critical care (n=2045). In the “Infant Subset,” we present data on live births to pregnant women with COVID-19, including outcomes on preterm birth (n=2626)

d Data from CANCOVID-Preg represents all provinces, with missing data randomly distributed across provinces except for the risk factor “pre-existing hypertension,” which is unavailable for the full cohort from Ontario

e Note that for the UKOSS study, 100% of patients are hospitalized. However, the reason for hospitalization may not be COVID-19, and some participants presented at the hospital for an unrelated reason and were found to have an incidental COVID-19 infection

f For the AFREhealth study, gestational age at COVID-19 onset was not recorded. Here, we present trimester of hospital admission as a proxy. N=41 were missing trimester of hospital admission (12%). However, the study is not included in the risk factor analysis for gestational age at onset

g Antibody testing at antenatal care (Cohort I) and at labor and delivery (Cohort II) was the primary method of diagnosis; thus, gestational age at COVID-19 onset is unknown for almost all observations.

The mean maternal age across studies was 29.4 years, ranging from 26 years in Kenya (V.A., B.A.T.B.) and India (H.D.) to 32 years in Italy (E.B., V.L.L.). Among the 18 studies that recorded gestational age at SARS-CoV-2 infection, 11 recruited most of their participants in the third trimester; 10 of these studies included people in the postpartum period. The Nachega (multicountry Africa) and Yang (China) studies were composed entirely of patients hospitalized for COVID-19; the Knight (United Kingdom) and Poon (Hong Kong, China) studies were composed entirely (or almost entirely) of patients hospitalized for COVID-19, labor and delivery, or other causes (Table 1).

### Risk of bias of included studies

Detailed risk of bias ratings for each participating study are presented in summary in Table S4 and in detail in Table S5. Studies generally had moderate-to-low risk of bias according to the adapted Newcastle–Ottawa scale criteria, with 15 of 21 studies earning at least 4 out of 5 or 4 out of 6 stars across all outcome categories where that study was included in the analysis. The most common cause for high risk of bias rating was related to representativeness of the study population; 5 of 21 studies did not collect data on the reason for screening for individual patients. Another 8 studies primarily used methods to identify cases that were deemed to be at higher risk of bias (such as testing for clinical concern based on symptoms or travel). In total, 13 of 21 studies had elevated risk of bias in this area.

### Synthesis of results

#### Overall incidence

**Figure 2:**
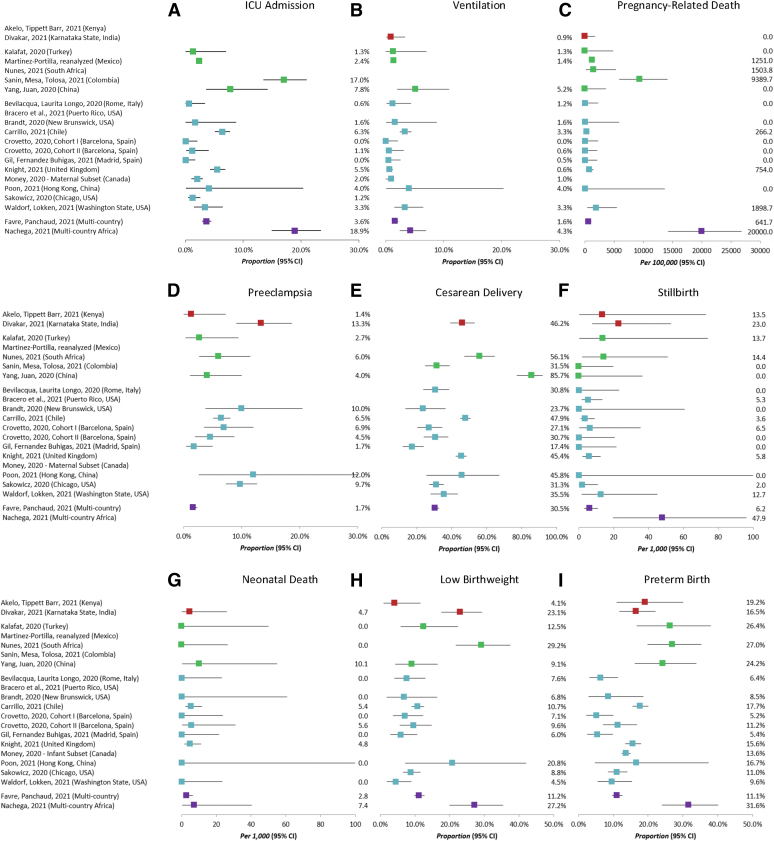
Incidence of outcomes by study The incidence and 95% confidence intervals of selected adverse outcomes across the 21 participating studies, including: **A,** intensive care unit admission, **B,** ventilation, **C,** pregnancy-related death, **D,** preeclampsia, **E,** cesarean delivery, **F,** stillbirth, **G,** neonatal death, **H,** low birthweight, and **I,** preterm birth. Studies are grouped by World Bank income group levels: lower-middle–income countries are shown in *red*; upper-middle–income countries are shown in *green*; high-income countries are shown in *blue*. Two studies (shown in *purple*) are multicountry studies that contain countries from multiple income groups. The complete list of countries for each of these multicountry studies is presented in Table 1. *Smith. Individual patient data meta-analysis: risk factors among COVID-19 pregnancies. Am J Obstet Gynecol 2023.*

Overall event incidence for each site is shown in Figure 2. There was considerable heterogeneity between studies for most assessed outcomes. This is likely owing to a combination of factors including varying sampling frames across studies, true differences in the incidence of outcomes in the general population, and underlying differences in the standard of care provided by health systems in each setting.

#### Comorbidities

We found that pregnant women with COVID-19 who also had chronic illnesses, including diabetes mellitus, hypertension, and cardiovascular disease, were at higher risk for most outcomes related to COVID-19 severity, and pregnancy-related death (Table 2). Risk of mortality was 3.79 times higher for pregnant women with preexisting diabetes mellitus (95% CI, 2.61–5.50; 15 studies, 15,705 pregnancies) (Table S7), 2.75 times higher for those with preexisting hypertension (95% CI, 1.76–4.28; 14 studies, 15,705 pregnancies) (Table S8), and 16.76 times higher for those with cardiovascular disease (95% CI, 4.42–63.64; 11 studies, 15,368 pregnancies) (Table S9) compared with those without these chronic health conditions.

**Table 2:** Relative risks and 95% confidence intervals comparing women with each risk factor with women without the risk factor: comorbidites

Outcome	N	Diabetes mellitus	N	Hypertension	N	CVD	N	HIV coinfection
		Pooled RR (95% CI)		Pooled RR (95% CI)		Pooled RR (95% CI)		Pooled RR (95% CI)
COVID-19 severity and mortality								
ICU admission	16	2.55 (1.97–3.31)	14	2.10 (1.63–2.70)	12	2.98 (1.83–4.85)	3	1.67 (1.06–2.63)
Ventilation	14	5.88 (2.77–12.48)	13	4.87 (2.93–8.09)	12	6.11 (2.85–13.08)	3	1.01 (0.30–3.32)
Critical care	14	3.03 (1.86–4.92)	12	2.42 (1.73–3.39)	11	2.82 (1.78–4.48)	3	1.72 (1.10–2.69)
Pneumonia	10	2.02 (1.65–2.47)	8	2.13 (1.74–2.61)	8	1.18 (0.65–2.16)	1	—
Pregnancy-related death	15	3.79 (2.61–5.50)	14	2.75 (1.76–4.28)	11	16.76 (4.42–63.64)	4	2.70 (0.58–12.47)
Maternal morbidity								
Hemorrhage	7	1.89 (0.96–3.70)	7	1.33 (0.60–2.94)	5	2.42 (0.29–20.01)	3	1.06 (0.57–1.99)
Placental abruption	6	7.25 (2.47–21.25)	6	6.68 (2.35–18.98)	3	9.99 (1.70–58.58)	2	—
Preeclampsia	10	2.98 (1.61–5.51)	9	5.80 (4.11–8.19)	8	4.78 (2.24–10.22)	2	—
Preeclampsia or eclampsia	7	4.32 (1.58–11.84)	6	4.09 (2.08–8.07)	6	6.38 (2.80–14.58)	2	—
Hypertensive disorders of pregnancy (any)	9	2.73 (1.62–4.58)	8	3.16 (2.24–4.47)	8	4.29 (2.17–8.48)	2	—
Hypertensive disorders of pregnancy (at/after COVID-19)	2	—	2	—	1	—	0	—
Preterm labor	8	3.54 (1.89–6.61)	7	3.93 (1.44–10.75)	8	3.94 (1.39–11.19)	2	—
Preterm labor with onset before 37 wk GA^a^	6	2.48 (1.24–4.98)	5	2.16 (0.73–6.40)	5	2.40 (0.31–18.46)	2	—
Cesarean delivery	12	1.40 (1.13–1.74)	11	1.31 (1.09–1.57)	10	1.44 (1.08–1.92)	3	1.51 (1.00–2.28)
Intrapartum cesarean delivery	9	1.30 (0.90–1.87)	8	1.58 (1.23–2.04)	9	1.59 (1.03–2.48)	3	1.47 (0.91–2.37)
Fetal and neonatal mortality and morbidity								
Stillbirth^b^	16	6.53 (2.13–20.05)	15	3.43 (1.41–8.37)	12	9.10 (2.24–36.92)	4	2.97 (0.35–25.26)
Perinatal death	12	7.71 (2.12–28.03)	11	4.94 (2.07–11.81)	10	8.47 (2.70–26.53)	3	8.63 (1.40–53.31)
Early neonatal death	12	6.97 (1.07–45.27)	11	11.74 (3.23–42.70)	10	12.58 (2.69–58.80)	3	—
Neonatal death^c^	13	6.85 (1.22–38.49)	12	8.10 (2.71–24.25)	10	13.04 (3.18–53.43)	4	—
NICU admission at birth	8	1.83 (1.15–2.93)	6	2.28 (1.26–4.13)	5	2.02 (0.65–6.30)	1	—
Adverse birth outcomes								
Very low birthweight (<1500 g)	13	5.28 (2.62–10.63)	12	6.30 (3.16–12.55)	10	8.35 (3.64–19.19)	4	2.41 (0.80–7.20)
Low birthweight (<2500 g)	13	1.80 (1.21–2.69)	12	1.87 (1.39–2.50)	10	2.01 (1.19–3.39)	4	1.38 (0.93–2.04)
Small for gestational age (3rd)	14	4.11 (1.53–11.06)	13	3.34 (1.86–6.00)	11	3.14 (1.58–6.23)	4	2.14 (1.02–4.48)
Small for gestational age (10th)	14	1.62 (0.81–3.21)	13	1.91 (1.29–2.84)	11	1.84 (1.11–3.03)	4	1.57 (0.93–2.63)
Moderate preterm birth (<34 wk)	14	3.23 (2.09–5.01)	13	3.55 (2.48–5.08)	11	3.04 (1.57–5.91)	4	1.78 (0.67–4.74)
Moderate preterm birth (<34 wk) with onset before 34 wk GA^a^	8	2.03 (1.24–3.31)	7	2.23 (1.46–3.41)	5	2.27 (0.93–5.50)	3	2.18 (0.93–5.07)
Preterm birth (<37 wk)	15	2.25 (1.77–2.86)	14	2.22 (1.72–2.86)	12	1.90 (1.41–2.56)	4	1.22 (0.83–1.81)
Preterm birth (<37 wk) with onset before 37 wk GA	8	1.40 (0.97–2.01)	7	1.61 (1.21–2.12)	6	1.25 (0.63–2.49)	3	1.40 (0.81–2.41)

Relative risks are calculated by pooling unadjusted relative risks from all participating studies with at least 1 adverse event for the given outcome using a DerSimonian–Laird random-effects model meta-analysis. For any study with 0 events in one arm (Risk Group or Reference Group), we used a continuity correction of the inverse of the number of events in the oppposite group within the same study.

*CI*, confidence interval; *GA*, gestational age; *ICU*, intensive care unit; *NICU*, neonatal intensive care unit; *RR*, relative risk.

*Smith. Individual patient data meta-analysis: risk factors among COVID-19 pregnancies. Am J Obstet Gynecol 2023.*

a These outcomes (preterm labor, moderate preterm birth before 34 weeks’ gestation, and preterm birth before 37 weeks’ gestation) were included in the sensitivity analyses where we restricted confirmed COVID-19 cases to those with confirmed COVID-19 onset before 37 weeks’ gestation (or 34 weeks for very moderate preterm birth). The full comparison group was used for each of the sensitivity analyses

b The outcome presented here is stillbirths occurring at ≥28 weeks’ gestation per the World Health Organization definition

c The outcome “neonatal death” was reported by 15 participating studies. However, most studies were not designed to follow up neonates until 28 days after birth. Therefore, counts of neonatal death are underestimated.

Pregnant women with COVID-19 and one of these chronic conditions were at higher risk for maternal morbidity, including placental abruption, preeclampsia, preeclampsia or eclampsia, hypertensive disorders of pregnancy, preterm labor, and any cesarean delivery. Those with hypertension or cardiovascular disease were also at increased risk of having an intrapartum cesarean delivery. Infants born to mothers with both COVID-19 and one of these chronic conditions were at higher risk for mortality (stillbirth, perinatal death, and neonatal death) and NICU admission. These infants were more likely to be born preterm, have low birthweight, and be small for gestational age.

Although less data were available on HIV coinfection with COVID-19 during pregnancy, we found that coinfection increased the risk of severe COVID-19 disease (Table 2). Among pregnant women with COVID-19, those with HIV had a 67% increased risk of being admitted to the ICU (95% CI, 1.06–2.63; 3 studies, 2150 pregnancies) and 72% increased risk of needing critical care (95% CI, 1.10–2.69; 3 studies, 2150 pregnancies). Those with both COVID-19 and HIV were more likely to be delivered by cesarean delivery (RR, 1.51; 95% CI, 1.00–2.28; 3 studies, 1688 pregnancies), and infants born to those with HIV coinfection were at increased risk for perinatal death (RR, 8.63; 95% CI, 1.40–53.31; 3 studies, 1727 fetuses or infants) (Table S10).

#### Nutritional status and body mass index

We found increased risk of COVID-19 severity among pregnant and postpartum people who were either obese or underweight compared with those who were normal to overweight before pregnancy (Table 3). Pregnant women with a prepregnancy or early-pregnancy BMI of ≥30 kg/m^2^ were at increased risk for ICU admission (RR, 1.81; 95% CI, 1.26–2.60), ventilation (RR, 2.05; 95% CI, 1.20–3.51), and pneumonia (RR, 1.66; 95% CI, 1.18–2.33), but not for pregnancy-related death (RR, 1.00; 95% CI, 0.19–5.26) (Table S11).

**Table 3:** Relative risks and 95% confidence intervals comparing women with each risk factor with women without the risk factor: nutrition-related factors

		Obese		Underweight		Anemia
Outcome	N	Pooled RR (95% CI)	N	Pooled RR (95% CI)	N	Pooled RR (95% CI)
COVID-19 severity and mortality						
ICU admission	8	1.81 (1.26–2.60)	8	5.53 (2.27–13.44)	4	1.67 (1.28–2.19)
Ventilation	7	2.05 (1.20–3.51)	7	9.36 (3.87–22.63)	4	1.78 (1.02–3.12)
Critical care	7	1.89 (1.28–2.77)	7	5.71 (2.40–13.59)	3	—
Pneumonia	5	1.66 (1.18–2.33)	5	2.71 (1.13–6.49)	2	—
Pregnancy-related death	7	1.00 (0.19–5.26)	7	14.10 (2.83–70.36)	5	2.36 (1.15–4.81)
Maternal morbidity						
Hemorrhage	4	1.43 (0.85–2.41)	4	6.00 (0.89–40.41)	2	—
Placental abruption	2	—	2	—	2	—
Preeclampsia	4	1.60 (1.01–2.54)	4	2.18 (0.63–7.53)	3	—
Preeclampsia or eclampsia	3	2.16 (0.68–6.82)	3	3.08 (0.64–14.81)	3	—
Hypertensive disorders of pregnancy (any)	5	1.86 (1.30–2.67)	5	1.93 (0.59–6.26)	3	0.87 (0.52–1.46)
Hypertensive disorders of pregnancy (at/after COVID-19)	1	—	1	—	1	—
Preterm labor	6	0.91 (0.57–1.46)	6	3.76 (0.95–14.82)	2	—
Preterm labor with onset before 37 wk GA^a^	4	0.84 (0.51–1.39)	3	0.62 (0.02–18.50)	2	—
Cesarean delivery	7	1.23 (1.07–1.41)	7	1.15 (0.54–2.45)	4	0.75 (0.47–1.19)
Intrapartum cesarean delivery	6	1.28 (1.06–1.56)	6	1.42 (0.26–7.78)	3	0.67 (0.28–1.62)
Fetal and neonatal mortality and morbidity						
Stillbirth^b^	8	1.89 (0.31–11.60)	8	—	5	3.75 (1.00–14.11)
Perinatal death	6	3.17 (0.43–23.21)	6	—	3	—
Early neonatal death	6	—	6	—	3	—
Neonatal death^c^	6	—	6	—	4	2.98 (0.49–18.13)
NICU admission at birth	4	1.42 (0.82–2.47)	4	2.21 (0.26–18.78)	2	—
Adverse birth outcomes						
Very low birthweight (<1500 g)	6	1.70 (0.76–3.79)	6	14.81 (3.25–67.39)	4	1.64 (0.47–5.73)
Low birthweight (<2500 g)	6	0.97 (0.68–1.37)	6	1.98 (0.74–5.26)	4	0.99 (0.60–1.62)
Small for gestational age (3rd)	6	0.68 (0.24–1.95)	6	7.14 (1.98–25.73)	4	1.11 (0.56–2.21)
Small for gestational age (10th)	6	0.75 (0.41–1.37)	6	2.46 (0.90–6.70)	4	0.99 (0.64–1.53)
Moderate preterm birth (<34 wk)	6	1.75 (1.06–2.89)	6	7.53 (2.33–24.29)	4	0.91 (0.51–1.61)
Moderate preterm birth (<34 wk) with onset before 34 wk GA^a^	3	1.46 (0.89–2.40)	2	—	2	—
Preterm birth (<37 wk)	7	1.38 (1.10–1.73)	7	1.58 (0.59–4.26)	4	0.94 (0.67–1.32)
Preterm birth (<37 wk) with onset before 37 wk GA^a^	3	1.17 (0.90–1.51)	2	—	3	0.92 (0.62–1.37)

Relative risks are calculated by pooling unadjusted relative risks from all participating studies with at least 1 adverse event for the given outcome using a DerSimonian–Laird random-effects model meta-analysis. For any study with 0 events in one arm (Risk Group or Reference Group), we used a continuity correction of the inverse of the number of events in the oppposite group within the same study.

*CI*, confidence interval; *GA*, gestational age; *ICU*, intensive care unit; *NICU*, neonatal intensive care unit; *RR*, relative risk.

*Smith. Individual patient data meta-analysis: risk factors among COVID-19 pregnancies. Am J Obstet Gynecol 2023.*

a These outcomes (preterm labor, moderate preterm birth before 34 weeks’ gestation, and preterm birth before 37 weeks’ gestation) were included in the sensitivity analyses where we restricted confirmed COVID-19 cases to those with confirmed COVID-19 onset before 37 weeks’ gestation (or 34 weeks for very moderate preterm birth). The full comparison group was used for each of the sensitivity analyses

b The outcome presented here is stillbirths occurring at ≥28 weeks’ gestation per the World Health Organization definition

c The outcome “neonatal death” was reported by 15 participating studies. However, most studies were not designed to follow up neonates until 28 days after birth. Therefore, counts of neonatal death are underestimated.

Pregnant women who were underweight prepregnancy had >5 times increased risk for ICU admission (RR, 5.53; 95% CI, 2.27–13.44; 8 studies, 1721 pregnancies) or any critical care (RR, 5.71; 95% CI, 2.40–13.59; 7 studies, 1822 pregnancies), >9 times increased risk for ventilation (RR, 9.36; 95% CI, 3.87–22.63; 7 studies, 1822 pregnancies), and nearly 3 times increased risk for pneumonia (RR, 2.71; 95% CI, 1.13–6.49; 5 studies, 1129 pregnancies) compared with pregnant women who were normal to overweight prepregnancy (Table S12). Although this was based on a small sample size, underweight pregnant women with COVID-19 were found to have a sharply increased risk of pregnancy-related death (RR, 14.10; 95% CI, 2.83–70.36; 7 studies, 700 pregnancies).

Prepregnancy obesity was also associated with increased risks for maternal morbidity such as preeclampsia (RR, 1.60; 95% CI, 1.01–2.54), any hypertensive disorders of pregnancy (RR, 1.86; 95% CI, 1.30–2.67), any cesarean delivery (RR, 1.23; 95% CI, 1.07–1.41), and intrapartum cesarean delivery (RR, 1.28; 95% CI, 1.06–1.56) (Table 3). Prepregnancy underweight was associated with adverse birth outcomes such as very low birthweight (RR, 14.81; 95% CI, 3.25–67.39), small-for-gestational age infants in the third percentile (RR, 7.14; 95% CI, 1.98–25.73), and moderately preterm birth (RR, 7.53; 95% CI, 2.33–24.29).

Although data were limited, we found an increased risk of COVID-19 severity among pregnant women with anemia at the time of COVID-19 diagnosis compared with those without anemia (Table 3). Those with anemia had an increased risk of ICU admission (RR, 1.67; 95% CI, 1.28–2.19; 4 studies, 1089 pregnancies), ventilation (RR, 1.78; 95% CI, 1.02–3.12; 4 studies, 974 pregnancies), and death (RR, 2.36; 95% CI, 1.15–4.81; 5 studies, 809 pregnancies). We also found an increased risk of stillbirth for pregnant women with anemia (RR, 3.75; 95% CI, 1.00–14.11; 5 studies, 748 fetuses or infants) (Table S13).

### Maternal age

Older maternal age (35–45 years) was associated with multiple COVID-19–associated adverse outcomes compared with those aged 20 to 34 years (Table 4). Older maternal age was associated with increased risk of ICU admission (RR, 1.60; 95% CI, 1.36–1.89; 16 studies, 18,758 pregnancies), ventilation (RR, 2.13; 95% CI, 1.68–2.71; 16 studies, 18,407 pregnancies), any critical care (RR, 1.62; 95% CI, 1.38–1.90; 15 studies, 18,452 pregnancies) (Table S14), and pneumonia diagnosis (RR, 1.51; 95% CI, 1.35–1.70; 10 studies, 15,670 pregnancies). Older pregnant women also had increased risk for placental abruption (RR, 3.94; 95% CI, 1.40–11.13) and cesarean delivery (RR, 1.21; 95% CI, 1.10–1.32). Infants born to older pregnant women with COVID-19 had higher risk of stillbirth, perinatal death, NICU admission, preterm birth, and low birthweight.

**Table 4:** Relative risks and 95% confidence intervals comparing women with each risk factor with women without the risk factor: maternal age and primiparity

		Age 15–19 years		Age 35–45 years		Primiparity
Outcome	N	Pooled RR(95% CI)	N	Pooled RR (95% CI)	N	Pooled RR (95% CI)
COVID-19 severity and mortality						
ICU admission	12	1.42 (0.53–3.77)	16	1.60 (1.36–1.89)	14	0.90 (0.71–1.13)
Ventilation	12	2.59 (0.79–8.51)	16	2.13 (1.68–2.71)	12	0.67 (0.39–1.16)
Critical care	11	1.24 (0.48–3.17)	15	1.62 (1.38–1.90)	12	0.82 (0.62–1.08)
Pneumonia	9	0.82 (0.62–1.08)	10	1.51 (1.35–1.70)	8	0.59 (0.46–0.77)
Pregnancy-related death	13	0.73 (0.27–1.94)	16	1.62 (0.81–3.24)	14	0.75 (0.45–1.25)
Maternal morbidity						
Hemorrhage	6	1.93 (0.94–3.98)	6	1.17 (0.82–1.68)	7	1.26 (0.90–1.77)
Placental abruption	5	--	6	3.94 (1.40–11.13)	6	0.64 (0.19–2.09)
Preeclampsia	10	2.03 (0.89–4.61)	13	1.12 (0.73–1.74)	11	2.10 (1.45–3.03)
Preeclampsia or eclampsia	8	3.27 (1.11–9.64)	9	0.93 (0.63–1.37)	8	1.75 (1.22–2.53)
Hypertensive disorders of pregnancy (any)	10	2.06 (0.77–5.55)	12	1.17 (0.93–1.49)	10	1.56 (1.13–2.15)
Hypertensive disorders of pregnancy (at/after COVID-19)	2	--	3	1.91 (0.45–8.16)	3	1.39 (0.54–3.57)
Preterm labor	8	2.48 (0.53–11.60)	10	1.39 (0.96–2.02)	8	0.86 (0.51–1.43)
Preterm labor with onset before 37 wk GA^a^	5	1.62 (0.42–6.22)	8	1.28 (0.87–1.87)	6	0.88 (0.51–1.51)
Cesarean delivery	10	0.86 (0.65–1.13)	13	1.21 (1.10–1.32)	12	1.00 (0.90–1.11)
Intrapartum cesarean delivery	9	0.90 (0.63–1.31)	10	1.03 (0.89–1.20)	8	1.35 (1.14–1.60)
Fetal and neonatal mortality and morbidity						
Stillbirth^b^	15	4.59 (1.69–12.45)	18	1.75 (0.92–3.33)	17	1.34 (0.62–2.90)
Perinatal death	11	4.80 (1.28–17.99)	14	1.53 (0.82–2.83)	12	1.78 (0.89–3.54)
Early neonatal death	11	5.94 (1.02–34.56)	14	1.80 (0.51–6.33)	12	1.60 (0.45–5.62)
Neonatal death^c^	12	9.38 (2.21–39.89)	15	1.96 (0.65–5.87)	13	1.25 (0.43–3.60)
NICU admission at birth	6	1.59 (0.48–5.23)	9	1.35 (1.12–1.63)	8	1.03 (0.85–1.25)
Adverse birth outcomes						
Very low birthweight (<1500 g)	13	6.27 (1.86–21.15)	16	1.39 (0.89–2.16)	14	1.03 (0.61–1.73)
Low birthweight (<2500 g)	13	0.96 (0.54–1.73)	16	1.24 (1.04–1.47)	14	1.27 (1.04–1.54)
Small for gestational age (3rd)	14	4.33 (1.87–10.06)	17	1.46 (1.01–2.12)	15	2.11 (1.42–3.11)
Small for gestational age (10th)	14	1.40 (0.83–2.36)	17	0.98 (0.79–1.21)	15	1.74 (1.41–2.15)
Moderate preterm birth (<34 wk)	14	3.06 (1.48–6.35)	17	1.51 (1.19–1.93)	15	1.10 (0.84–1.44)
Moderate preterm birth (<34 wk) with onset before 34 wk GA^a^	7	2.90 (1.18–7.14)	10	1.43 (1.07–1.90)	8	1.07 (0.74–1.53)
Preterm birth (<37 wk)	14	1.22 (0.84–1.78)	18	1.40 (1.19–1.64)	15	1.02 (0.87–1.19)
Preterm birth (<37 wk) with onset before 37 wk GA^a^	7	1.06 (0.68–1.67)	11	1.27 (1.07–1.50)	9	1.02 (0.83–1.26)

Relative risks are calculated by pooling unadjusted relative risks from all participating studies with at least 1 adverse event for the given outcome using a DerSimonian–Laird random-effects model meta-analysis. For any study with 0 events in one arm (Risk Group or Reference Group), we used a continuity correction of the inverse of the number of events in the oppposite group within the same study.

*CI*, confidence interval; *GA*, gestational age; *ICU*, intensive care unit; *NICU*, neonatal intensive care unit; *RR*, relative risk.

*Smith. Individual patient data meta-analysis: risk factors among COVID-19 pregnancies. Am J Obstet Gynecol 2023.*

a These outcomes (preterm labor, moderate preterm birth before 34 weeks’ gestation, and preterm birth before 37 weeks’ gestation) were included in the sensitivity analyses where we restricted confirmed COVID-19 cases to those with confirmed COVID-19 onset before 37 weeks’ gestation (or 34 weeks for very moderate preterm birth). The full comparison group was used for each of the sensitivity analyses

b The outcome presented here is stillbirths occurring at ≥28 weeks’ gestation per the World Health Organization definition

c The outcome “neonatal death” was reported by 15 participating studies. However, most studies were not designed to follow up neonates until 28 days after birth. Therefore, counts of neonatal death are underestimated.

Compared with pregnant women with COVID-19 aged 20 to 34 years, younger pregnant women (15–19 years) were at increased risk for preeclampsia or eclampsia (RR, 3.27; 95% CI, 1.11–9.64; 8 studies, 1074 pregnancies) (Table S15). Infants born to younger women with COVID-19 had higher risks of stillbirth, perinatal death, and neonatal death. Younger women with COVID-19 were also more likely to experience adverse pregnancy outcomes, including moderate preterm birth (RR, 2.90; 95% CI, 1.18–7.14; 7 studies, 1321 infants), very low-birthweight infants (RR, 6.27; 95% CI, 1.86–21.15; 13 studies, 3203 infants), and small-for-gestational-age infants (<3rd percentile; RR, 4.33; 95% CI, 1.87–10.06, 14 studies, 3901 infants).

#### Primiparity

Overall, we found limited differences in risks of adverse outcomes among primiparous compared with multiparous pregnant women with COVID-19 (Table 4). Primiparous women were less likely to be diagnosed with pneumonia than multiparous women (RR, 0.59; 95% CI; 0.46–0.77; 8 studies, 4249 pregnancies) and were more likely to experience preeclampsia or eclampsia, any hypertensive disorders of pregnancy, or intrapartum cesarean delivery, compared with multiparous women (Table S16).

#### Symptomatic SARS-CoV-2 infection

We found increased risks for adverse outcomes related to COVID-19 severity among pregnant women with symptomatic infection compared with those with asymptomatic SARS-CoV-2 infection, including ICU admission, any critical care, and pneumonia (Table S17). However, most other outcomes related to maternal morbidity, fetal and neonatal mortality and morbidity, and adverse birth outcomes were similar across symptomatic and asymptomatic groups, with a few exceptions. Pregnant women with symptomatic COVID-19 were more likely to have an intrapartum cesarean delivery (RR, 1.25; 95% CI, 1.05–1.48) compared with those with asymptomatic infection (Table S18).

We also found increased risk of preterm and moderate preterm birth among symptomatic pregnant women (RR, 1.30; 95% CI, 1.06–1.60 and RR, 1.65; 95% CI, 1.00–2.73, respectively). However, when we restricted to only pregnant women with infection onset before 37 weeks’ gestation for preterm birth and before 34 weeks’ gestation for moderate preterm birth, we found that asymptomatic pregnant women had an increased risk of preterm and moderate preterm birth (RR, 0.71; 95% CI, 0.52–0.97 and RR, 0.57; 95% CI, 0.41–0.81) compared with symptomatic pregnant women.

## Comment

### Principal findings

As in the general population, we found that pregnant women with comorbidities including diabetes mellitus, hypertension, cardiovascular disease, and obesity were at increased risk for severe COVID-19–related outcomes, as well as for maternal morbidities and adverse birth outcomes, compared with pregnant women without these comorbidities. Given pooled global data, we also identified several less commonly known risk factors for pregnant women with COVID-19, including HIV coinfection, being underweight at the start of pregnancy, and anemia at the time of COVID-19 diagnosis.

### Comparison with existing literature

We found that among pregnant women with COVID-19, those living with HIV were nearly twice as likely to be admitted to the ICU or need critical care. Women living with HIV already have greater likelihood of antenatal, delivery, and postpartum complications, including preterm birth, cesarean delivery, postpartum sepsis, venous thromboembolism, postpartum infection, and mortality.^24^ Neonates born to these women are at higher risk owing to prematurity, low birthweight, and intrauterine growth restriction, resulting in higher rates of NICU admission and neonatal mortality.^24^^,^^25^ Factors related to HIV severity such as HIV progression, antiretroviral therapy, CD4 cell count, and viral load additionally affect the immune response to coinfection.^26^

A recent systematic review of SARS-CoV-2 infection among people living with HIV in the general population found strong evidence that HIV is a risk factor for both SARS-CoV-2 infection and for mortality owing to COVID-19; that review did not examine pregnant and postpartum women as a subgroup of interest.^27^ Given that pregnant women are at higher risk for severe COVID-19 illness and complications from HIV, SARS-CoV-2 and HIV coinfection may be particularly concerning in this population. However, our analysis of COVID-19 infection among pregnant women living with HIV had several limitations. First, we do not yet have sufficient data to examine either treatment status or viral load among pregnant women with HIV; thus, we cannot shed light on how these factors could mediate excess risk. Furthermore, adverse outcomes related to both COVID-19 severity and pregnancy outcomes can be affected by social, behavioral, and structural factors prevalent in HIV-endemic regions.^28^

Undernutrition in pregnant women with COVID-19 was identified as an important risk factor for COVID-19 severity and adverse birth outcomes. Underweight pregnant women had elevated risks for severe COVID-19 and pregnancy-related death, and for moderately preterm, very low-birthweight, and small-for-gestational-age infants. In addition, being anemic during pregnancy increased the risk for pregnancy-related death, ICU admission, and stillbirth. Although the results for anemia were based on 4 studies, the effect estimates for severe COVID-19 were consistent with those reported in a recent meta-analysis highlighting linkages between low hemoglobin, hypoxia, respiratory organ dysfunction, and severe outcomes from COVID-19 infection in the general population.^10^ In pregnant and nonpregnant women, single or multiple nutritional deficiencies are known to decrease immune responses, consequently increasing the risk of infection, disease severity, and morbidity and mortality.^29^, ^30^, ^31^ These linkages are especially important during pregnancy when the demand for macro- and micronutrients to support maternal physiological functioning, placental development, and fetal growth is even higher.^32^ Failure to meet these demands has been linked to preterm births and stillbirths in both high-income^33^, ^34^, ^35^ and low- and middle-income countries.^36^ These indicators of undernutrition are generally linked to many different health conditions (eg, iron deficiency, other infections), and it is difficult to infer specific mechanisms of action on the basis of this analysis. Nonetheless, our findings on the association between undernutrition or anemia and preterm births and stillbirths among pregnant women with COVID-19 further underscore the need for close monitoring and management of this group, including provision of additional nutritional support to prevent disease and adverse birth outcomes.^35^^,^^37^

We found that pregnant women with any COVID-19 symptoms were at increased risk for ICU admission, ventilation, cesarean delivery, and preterm birth compared with asymptomatic pregnant women on the basis of a large sample size of global studies. Although a previous systematic review of published literature examined this question, data on symptomatic compared with asymptomatic SARS-CoV-2 infection in pregnancy were only available for a small subset of studies and participants in this review (4 studies on ICU admission with 1178 participants, 3 studies on mechanical ventilation with 1023 participants, and 9 studies on cesarean delivery and preterm birth with 4233 participants).^5^ Our study found that symptomatic pregnant women are more likely to give birth preterm than asymptomatic pregnant women with SARS-CoV-2 infection.

However, in a sensitivity analysis restricted only to participants infected before 37 weeks’ gestation, we found that asymptomatic pregnant women are more likely than symptomatic pregnant women to have a preterm birth. These seemingly conflicting results may be related to features of study sampling; for example, this difference may be because of the large percentage of asymptomatic participants who are identified during screening at labor and delivery. Across the 10 studies included in the restricted analysis, 64% of infants born to asymptomatic participants were identified at ≥37 weeks’ gestation vs 26% of infants born to symptomatic participants.

### Strengths and limitations

IPD meta-analyses are considered the gold-standard method for generating aggregate estimates. Here, we standardized data quality assessment and harmonized definitions of risk factors and outcomes. This is especially valuable for outcomes such as stillbirth, preterm birth, and perinatal mortality, which have varying definitions globally. We included data from 33 countries and territories, including many low- and middle-income countries, whereas the bulk of the published literature on COVID-19 in pregnancy comes from middle- or high-income countries. Therefore, by pooling global data we were able to investigate risk factors such as HIV status, undernutrition, and anemia, which are more common in low-income countries, but for which individual studies may not have adequate power to draw meaningful conclusions. We were also able to identify risks linked to rare outcomes such as pregnancy-related death and stillbirth.

Our study had several limitations. First, the studies contributing to the IPD meta-analysis recruited participants differently, varying from hospital-based surveillance to universal screening during antenatal care. Furthermore, representativeness of the sample was deemed to be at elevated risk of bias for most studies because of limited information about identification and screening at the individual patient level or the use of identification strategies that are only somewhat representative of the population of interest. Some studies only recruited women admitted to the hospital with COVID-19 infection, whereas others included both symptomatic and asymptomatic women who tested positive for the infection. Given the heterogeneity of the sampling frames between studies, it is not possible to draw inferences about the absolute risk of adverse outcomes. The heterogeneity in baseline rates of adverse outcomes globally further complicates interpretation of the absolute risks. However, the RRs comparing those with and without the risk factors of interest generally seemed consistent between sites, and heterogeneity was relatively low for pooled estimates. In addition, although this analysis pooled a large, global sample of pregnant and postpartum women with COVID-19, half of the overall sample for critical care outcomes (ICU admission, ventilation, any critical care, pneumonia, and mortality) was derived from the Mexican National Registry, which collected no information on maternal morbidity, birth, or neonatal outcomes. This analysis also did not examine risk factors related to social determinants of health, which may exacerbate the biological risk factors identified in this analysis.

We identified risk factors for adverse maternal morbidities and fetal and neonatal outcomes among pregnant women with COVID-19, and these are generally consistent with risk factors for adverse pregnancy outcomes including preexisting diabetes mellitus or hypertension,^38^, ^39^, ^40^ cardiovascular disease,^41^ obesity,^40^^,^^42^ underweight,^42^^,^^43^ anemia,^44^^,^^45^ and HIV infection.^24^^,^^25^ Because the studies in this IPD meta-analysis only included individuals with SARS-CoV-2 infection, we were unable to evaluate whether the presence of infection confers additional risk beyond the risk because of risk factors without the presence of COVID-19 infection. Similarly, we identified risk factors for adverse COVID-19–related outcomes, and these are generally consistent with risk factors identified in the general nonpregnant population. Nonetheless, this study provides high-quality evidence that pregnant women with these risk factors are also at risk for adverse outcomes from COVID-19 illness.

### Conclusions and implications

Although pregnant women are already considered a high-risk population by the WHO and should be given equitable access to safe and effective preventives and therapeutics, special priority should be given to pregnant women with additional risk factors, including those related to chronic and infectious comorbidities, nutritional status, and maternal age. These data strongly support the need for access to vaccines and treatments for SARS-CoV-2 infection for pregnant women, prioritizing those with risk factors for severe illness and adverse birth outcomes.

## Supplementary Data

Video 1 <VIDSE>*Smith. Individual patient data meta-analysis: risk factors among COVID-19 pregnancies. Am J Obstet Gynecol 2022.*

Video 2 <VIDSE>*Smith. Individual patient data meta-analysis: risk factors among COVID-19 pregnancies. Am J Obstet Gynecol 2022.*
