# Supplementary material for: Clinical risk factors of adverse outcomes among women with COVID-19 in the pregnancy and postpartum period: a sequential, prospective meta-analysis
Source: Am J Obstet Gynecol. 2023 Feb;228(2):161–77. doi: 10.1016/j.ajog.2022.08.038 (PMC9398561; doi:10.1016/j.ajog.2022.08.038)
Supplement: Table S1 [file mmc3.docx]

Table S1. Summary of previously published studies and explanation of differences from prior publication in data submitted to the PMA

| ***PMA Data Contributor*** | ***Description of Re-analysis of Published Data*** | ***Citation*** |
| --- | --- | --- |
| Brandt, 2020 (USA - New Brunswick) | The data submitted to the PMA by Brandt are published in the study Brandt et al (2021). For this analysis, we re-analyze the data submitted according to the PMA protocol, using the full sample size of COVID-19 infected pregnant people (n=61). | Brandt, JS, Hill, J, Reddy, A, Schuster, M, Patrick, HS, Rosen, T, et al. Epidemiology of coronavirus disease 2019 in pregnancy: risk factors and associations with adverse maternal and neonatal outcomes. American Journal of Obstetric Gynecology. 2021 Apr; 224(4): 389.e1-389.e9. Available from: doi: 10.1016/j.ajog.2020.09.043 |
| Carrillo, 2021 (Chile) | A subset of the data submitted to the PMA by Carrillo from the GESTACOVID Chile study are originally published in the study Hernández et al, 2020. The original study included a sample of 661 pregnant patients with COVID-19 recruited from 23 hospitals in Chile between April 7, 2020 and July 6, 2020. In addition to this original subset of data, Carillo also submitted additional cases of COVID-19 in pregnancy recruited for the GESTACOVID study through November 2020. For this analysis, we re-analyze the data submitted according to the PMA protocol, retaining a sample of 1,347 COVID-positive cases with confirmed COVID-19 diagnosis during pregnancy or within 42 days postpartum. | Hernández, O., Honorato, M., Silva, M.C., Sepúlveda-Martínez, Á., Fuenzalida, J., Abarzúa, F., et al. COVID-19 and pregnancy in Chile: preliminary report of the GESTACOVID multicenter study. Rev. Chil. Obstet. Ginecol. 2020 Sept: 85(1): S75-S89. http://dx.doi.org/10.4067/S0717-75262020000700011 |
| Crovetto et al, 2020 (Spain) | The data submitted to the PMA by Crovetto et al are published in the study Crovetto et al (2020). For this analysis, we re-analyze the data submitted according to the PMA protocol. Further, for this analysis, Crovetto et al identified 32 additional COVID-positive observations among those who were initially identified as COVID-negative at early pregnancy screening and then tested positive during follow-up PCR testing at labor and delivery (Crovetto et al follow-up study forthcoming). For the PMA, we consider the Crovetto et al study as two separate cohort studies based on the distinct study designs for each cohort, with a sample size of n=173 for COVID-positive pregnancies in Cohort I and n=176 for COVID-positive pregnancies in Cohort II. | [Crovetto, F, Crispi, F, Llurba, E, Pascal, R, Larroya, M, Trilla, C, et al. Impact of SARS-CoV-2 infection on pregnancy outcomes: A population-based study. Clinical Infectious Diseases. 2021 Feb; 73(10): 1768-1775. Available from: https://doi.org/10.1093/cid/ciab104](https://doi.org/10.1093/cid/ciab104) |
| Favre, Panchaud, 2021 (COVI-Preg Study) | A subset of the data submitted to the PMA from the COVI-Preg study are originally published in the study Vouga et al., 2021. The original study included a sample of 1,079 pregnant patients with confirmed or suspected SARS-CoV-2 recruited to the internataional registry between March 24 and July 26, 2020. This sample included cases from 16 countries: Belgium, Brazil, Canada, Chile, Colombia, France, French Guyana, Germany, Ireland, Italy, Israel, Portugal, Spain, Switzerland, and the USA.  Investigators submitted this subset of data, as well as additional data reported by participating centers through December 2021, including data from centers in additional countries not included in the initial publication (Afghanistan, Albania, Argentina, China, Egypt, England, Indonesia, and Mexico). In order to avoid any potential duplication with other study sites, we worked with investigators to exclude data from centers and countries where duplication was possible or confirmed, including: Canada, Chile, China, Colombia, Italy (Rome), Spain (Barcelona), Mexico, UK, and USA). Ultimately, we worked with the COVI-Preg investigators to create a sample of n=2391 pregnant patients with confirmed SARS-CoV-2 infection recruited from participating centers in 14 countries.  In our re-analysis of the COVI-Preg study data, we updated the outcome "stillbirth" to conform with the PMA definition of death of the fetus before or during birth at or after 28 weeks gestational age (the original definition for this study was at or after 24 weeks). | [Vouga, M., Favre, G., Martinez-Perez, O., Pomar, L., Forcen Acebal, L., Abascal-Saiz, A., et al. Maternal outcomes and risk factors for COVID-19 severity among pregnant women. *Scientific Reports*. 2021 Jul; 11: 13898. Available from: https://doi.org/10.1038/s41598-021-92357-y](https://doi.org/10.1038/s41598-021-92357-y) |
| Kalafat 2020 (Turkey) | The data submitted to the PMA by Kalafat are also published as a subset of the data in the previously published study Kalafat, Yassa, Koc, Tug, and the TULIP Collaboration, 2020. For this analysis, we re-analyze the data submitted by Kalafat according to the PMA protocol, with a sample size of n=77 COVID-positive pregnancies (collected in Istanbul, Turkey). | [Kalafat, E, Yassa, M, Koc, A, Tug, N, the TULIP collaboration. Utility of lung ultrasound assessment for probable SARS-CoV-2 infection during pregnancy and universal screening of asymptomatic individuals. Ultrasound in Obstetrics & Gynecology. 2020 Sept; 56(4): 624-626. Available from: https://doi.org/10.1002/uog.23099](https://doi.org/10.1002/uog.23099) |
| Knight, 2021 (UK) | A subset of the data submitted to the PMA by Knight are originally published in the study Knight et al, 2020. The overlapping data include the COVID-positive pregnancies identified across 194 obstetric units in the United Kingdom between March 1 2020 and April 14, 2020 (n=427). Beyond this initial data, Knight also submitted additional cases of COVID-19 in pregnancy identified from the same 194 obstetric units in the United Kingdom through October 2020. For this analysis, we re-analyzed the data according to the PMA protocol, retaining all COVID-19 infected observations submitted, for a sample size of n= 1,243. | [Knight M, Bunch K, Vousden N, Morris E, Simpson N, Gale C et al. Characteristics and outcomes of pregnant women admitted to hospital with confirmed SARS-CoV-2 infection in UK: national population based cohort study. BMJ, 2020; 369 :m2107 https://doi.org/10.1136/bmj.m2107](https://doi.org/10.1136/bmj.m2107) |
| Martinez-Portilla, 2021 (Mexico) | A subset of the data submitted to the PMA by Martinez-Portilla is originally published in the study Martinez-Portilla et al (2020). The original study includes 5,183 cases of COVID-19 in pregnancy among women ages 15-45, recruited during the time period February through October 2020 from 475 monitoring hospitals participating in the COVID-19 National Data Registry of Mexico. Martinez-Portilla also submitted additional cases of COVID-19 in pregnancy among women ages 15-45 from the same source through March 2021 (n=5,848). For this analysis, we re-analyze all submitted cases of COVID-19 in pregnancy (n=11,031). | Martinez-Portilla, R.J., Sotiriadis, A., Chatzakis, C., Torres-Torres, J., Espino y Sosa, S., Sandoval-Mandujano, K., et al. Pregnant women with SARS-CoV-2 infection are at higher risk of death and pneumonia: propensity score matched analysis of a nationwide prospective cohort (COV19Mx). Ultrasound in Obstetrics & Gynecology, 2020 Dec: 57(2): 224-231. https://doi.org/10.1002/uog.23575 |
| Money, 2020 (Canada) | Data submitted to the PMA from the Cancovid-Preg study will also be published in a forthcoming article by Money et al. The forthcoming publication includes a larger sample size than submitted to the PMA, including cases recruited to the study after data submission for meta-analysis. | Forthcoming article (JAMA, 2022) |
| Nachega, 2021 (Multi-country Africa) | Data submitted to the PMA from the AFREHealth study of COVID-19 in pregnancy will also be published in a forthcoming article by Nachega et al. The forthcoming publication includes a larger sample size than submitted to the PMA, including cases recruited to the study after data submission for meta-analysis. | Forthcoming article (CID, 2022) |
| Sakowicz et al, 2021 (USA – Chicago) | A subset of the data submitted to the PMA by Sakowicz et al are published in the study Sakowicz et al, 2020. The overlapping data include all COVID-positive pregnancies delivered prior to May 31, 2020 (n=101). Sakowicz et al also submitted additional COVID-positive cases to the PMA delivered on or after June 1, 2020 (n=402) that are not included in this original publication. For this analysis, we re-analyzed the data according to the PMA protocol, retaining all COVID-19 infected observations submitted by Sakowicz et al (n=503). | Sakowicz, A, Ayala, AE, Ukeje, CC, Witting, CS, Grobman, WA, Miller, ES. Risk factors for severe acute respiratory syndrome coronavirus 2 infection in pregnant women. American Journal of Obstetrics and Gynecology MFM. 2020 Nov; 2(4): 100198. Available from: doi: 10.1016/j.ajogmf.2020.100198 |
| Waldorf, Lokken, 2021 (USA) | The data submitted to the PMA by Waldorf and Lokken are originally published in the study Lokken et al, 2021. For this analysis, we re-analyzed the data according to the PMA protocol, retaining the full sample size of n= 240. | Lokken, E.M., Huebner, E.M., Gray Taylor, G., Hendrickson, S., Vanderhoeven, J., Kachikis, A., et al. Disease severity, pregnancy outcomes, and maternal deaths among pregnant patients with severe acute respiratory syndrome coronavirus 2 infection in Washington State. Am J Obstet Gynecol, 2021 Jul: 225(1): 77.e1-77.e14. doi: 10.1016/j.ajog.2020.12.1221 |

Table S2. Description of the adapted Newcastle Ottawa scale for assessing risk of bias - Risk Factor Analysis

| **Bias Level** | **Participant Selection** | | **Exposure Assessment (Risk Factors)** | | **Outcome Assessment** | |
| --- | --- | --- | --- | --- | --- | --- |
|  | **Representativeness of the population (SARS-CoV-2 Infected pregnant people)** | **Confirmation of SARS-CoV-2 Infection** | **Assessment of risk factor and outcome** | **Adequacy of data completeness (by risk factor group)** | **Adequacy of follow up for pregnancy outcome** | **Adequacy of data completeness (by outcome group)** |
| **Lower risk of bias  (earns a quality star (*) in the Newcastle Ottawa Scale)** | a) At least 50% of the cases are identified using a method truly representative of the COVID-exposed (confirmed/suspected) pregnant persons in the community  (e.g., Pregnant women universally tested when presenting for delivery at the hospital; Pregnant women universally tested during antenatal care (ANC) as part of routine screening) | a) Viral test indicating active infection (e.g., PCR test, antigen test)    b) Serology/antibody test with confirmed onset during pregnancy based on date of pandemic and gestational age | a) Medical records (including electronic medical records) or hospital charts | a) Complete risk factor data collected - data available for > 99% of participants   b) Subjects with missing risk factor data unlikely to introduce bias (<10% of participants with missing data) | a) >90% of pregnancy outcomes ascertained | a) Complete follow up - data available for >99% of participants    b) Subjects lost to follow up unlikely to introduce bias (<10% of participants with missing data) |
| **Higher risk of bias** | b) 50% or more of the cases are identified using a method a somewhat representative of the COVID+ pregnant persons in the community  (e.g., Pregnant women tested at delivery based on symptoms or travel; Pregnant women tested at ANC based on symptoms or travel; Pregnant women tested for antibodies during routine screening; Medical records of pregnant women hospitalized based on symptoms)  c) selected group of pregnancies (e.g. nurses, volunteers) s | c) Clinical diagnosis or radiography consistent with WHO case definitions of probably and suspected cases  d) Self report | b) Self report | c) Data missing for 11-25% of participants    d) > 25% of participants are missing data for risk factors  e) Risk factor data not collected (excluded from analysis) | b) 75-90% of pregnancy outcomes ascertained  c) <75% of pregnancy outcomes ascertained | c) Data missing for 11-25% of participants    d) > 25% of participants are missing data (outcome is excluded from this analysis) |

Table S3. Definition of outcomes

| **Outcome Group** | **Outcome** | **Definition** |
| --- | --- | --- |
| COVID-19 Severity & Mortality | ICU admission | Admission to Intensive Care Unit (ICU) or the equivalent for any reason during pregnancy or within 42 days postpartum |
|  | Ventilation | Any ventilation (mechanical or non-mechanical) received |
|  | Critical Care | Any critical care recevied, as defined by each paritcipating study (note that the majority of studies defined "critical care" as either admission to ICU OR receipt of any ventilation). |
|  | Pneumonia | Pneumonia diagnosis, as defined by each participating study |
|  | Pregnancy-related death | Death due to any cause during pregnancy or within 42 days postpartum **^1^** |
| Maternal Morbidities | Haemorrhage | Haemorrhage at the time of labor, as defined by each participating study |
|  | Placental Abruption | Placental abruption, as defined by each participating study |
|  | Preeclampsia | Preeclampsia, as defined by each participating study |
|  | Preeclampsia or Eclampsia | Preeclampsia or eclampsia, as defined by each participating study; we include this combined outcome because some study sites report only a single combined indicator. |
|  | Hypertensive Disorders of Pregnancy (Any) | Any diagnosis of hypertensive disorders of pregnancy during the current pregnancy, including preeclampsia, eclampsia, and HELLP. |
|  | Hypertensive Disorders of Pregnancy (At/After Covid-19) | Any diagnosis of hypertensive disorders of pregnancy during the current pregnancy, including preeclampsia, eclampsia, and HELLP, with a documented date of diagnosis at the same time or after COVID-19 diagnosis. |
|  | Preterm labor | Preterm labor (as defined by each participating study) before 37 weeks gestational age |
|  | Preterm labor with onset before 37w GA | Preterm labor (as defined by each participating study) before 37 weeks gestational age, restricting to those observations with documented gestational age at onset of COVID-19 less than 37 weeks gestational age |
|  | Cesarean Delivery | Any cesarean delivery, for any indication |
|  | Intrapartum Cesarean Delivery | Intrapartum or emergency cesarean delivery, as defined by each participating study, for any indication |
| Fetal and Neonatal Morbidity & Mortality | Stillbirth | Fetal death before or during birth at or after 28 weeks gestation **^2^** |
|  | Perinatal death | Fetal death before or during birth at or after 28 weeks gestation + early neonatal deaths (within the first 7 days of life) **3** |
|  | Early neonatal death | Neonatal death that occurs within the first 7 days of life **^4^** |
|  | Neonatal death | Neonatal death in the first 28 days of life |
|  | NICU Admission at Birth | Admission to a Neonatal Intensive Care Unit (or the equivalent) at birth |
| Adverse Birth Outcomes | Very low birthweight (<1500g) | Live born infant with a birthweight less than 1500g |
|  | Low birthweight (<2500g) | Live born infant with a birthweight less than 2500g |
|  | Small for gestational age (3rd) | Live born infant with a birthweight less than the 3rd percentile for his or her gestational age based on the sex-specific INTERGROWTH-21st reference values for size by gestational age **^5,6^** |
|  | Small for gestational age (10th) | Live born infant with a birthweight less than the 10th percentile for his or her gestational age based on the sex-specific INTERGROWTH-21st reference values for size by gestational age Where sex at birth is not available in a given study, we use the midpoint of the sex-specific reference values to determine size for gestational age. **^5,6^** |
|  | Moderate preterm birth (<34w) | Live born infant born at less than 34 weeks gestational age |
|  | Moderate preterm birth (<34w) with onset before 34w GA | Live born infant born at less than 34 weeks gestational age, restricting to those pregnancies where the mother had a documented COVID-19 diagnosis before 34 weeks gestational age |
|  | Preterm birth (<37 wks) | Live born infant born at less than 37 weeks gestational age |
|  | Preterm birth (<37 wks) with onset before 37w GA | Live born infant born at less than 37 weeks gestational age, restricting to those pregnancies where the mother had a documented COVID-19 diagnosis before 37 weeks gestational age |
| **1** As defined by the World Health Organization (see World Health Organization. : Maternal deaths. In: The Global Health Observatory: Indicator Metadata Registry List [Internet]. [cited 26 Jul 2021]. Available: <https://www.who.int/data/gho/indicator-metadata-registry/imr-details/4622>)  **2** As defined by the World Health Organization (see Tavares Da Silva F, Gonik B, McMillan M, Keech C, Dellicour S, Bhange S, et al. Stillbirth: Case definition and guidelines for data collection, analysis, and presentation of maternal immunization safety data. Vaccine. 2016;34: 6057–6068.)  **3** As defined by the World Health Organization (see Barfield WD, COMMITTEE ON FETUS AND NEWBORN. Standard Terminology for Fetal, Infant, and Perinatal Deaths. PEDIATRICS. 2016. pp. e20160551–e20160551. doi:10.1542/peds.2016-0551)  4 As defined by the World Health Organization (see Pathirana J, Muñoz FM, Abbing-Karahagopian V, Bhat N, Harris T, Kapoor A, et al. Neonatal death: Case definition & guidelines for data collection, analysis, and presentation of immunization safety data. Vaccine. 2016;34: 6027–6037.)  **5** See Villar J, Cheikh Ismail L, Victora CG, Ohuma EO, Bertino E, Altman DG, et al. International standards for newborn weight, length, and head circumference by gestational age and sex: the Newborn Cross-Sectional Study of the INTERGROWTH-21st Project. Lancet 2014, 384(9946):857-68. <https://doi.org/10.1016/S0140-6736(14)60932-6>  **6** Where sex at birth is not available in a given study, we use the midpoint of the sex-specific reference values to determine size for gestational age. | | |
|  |  |  |

Table S4. Summary of risk of bias of individual studies based on an adapted Newcastle Ottawa Scale

| **Study** | **Selection** | | **Exposure Assessment (Risk Factors)** | | **Outcome Assessment ^2^** | | | **Total (*)** | | |  |  |  |  |  |  |  |  |
| --- | --- | --- | --- | --- | --- | --- | --- | --- | --- | --- | --- | --- | --- | --- | --- | --- | --- | --- |
|  | **Representativeness of the population (SARS-CoV-2 Infected pregnant people)** | **Confirmation of SARS-CoV-2 Infection** | **Assessment of risk factor and outcome** | **Adequacy of Risk Factor Data ^1^** | **Adequacy of follow up of cohorts for pregnancy outcome** | **Adequacy of data completeness** | |  |  |  |  |  |  |  |  |  |  |  |
| **Martinez-Portilla, 2021** | **3** | ***** | ***** | ***** | **6** | **a) Critical Care Indicators** | ***** | 4/5 stars | | |  |  |  |  |  |  |  |  |
|  |  |  |  |  |  | **b) Maternal Mortality & Morbidity** | **N/A** | N/A | | |  |  |  |  |  |  |  |  |
|  |  |  |  |  |  | **c) Fetal & Neonatal Mortality & Morbidity** | **N/A** | N/A | | |  |  |  |  |  |  |  |  |
|  |  |  |  |  |  | **d) Adverse Birth Outcome** | **N/A** | N/A | | |  |  |  |  |  |  |  |  |
| **Favre, Panchaud, 2021** | **3** | ***** | ***** | ***** | **4** | **a) Critical Care Indicators** | ***** | 4/5 stars | | |  |  |  |  |  |  |  |  |
|  |  |  |  |  |  | **b) Maternal Mortality & Morbidity** | ***** | 4/6 stars | | |  |  |  |  |  |  |  |  |
|  |  |  |  |  |  | **c) Fetal & Neonatal Mortality & Morbidity** | ***** | 4/6 stars | | |  |  |  |  |  |  |  |  |
|  |  |  |  |  |  | **d) Adverse Birth Outcome** | ***** | 4/6 stars | | |  |  |  |  |  |  |  |  |
| **Money, 2020 - Maternal Subset** | **3** | ***** | ***** | **5** | **6** | **a) Critical Care Indicators** | ***** | 3/5 stars | | |  |  |  |  |  |  |  |  |
|  |  |  |  |  |  | **b) Maternal Mortality & Morbidity** | **N/A** | N/A | | |  |  |  |  |  |  |  |  |
|  |  |  |  |  |  | **c) Fetal & Neonatal Mortality & Morbidity** | **N/A** | N/A | | |  |  |  |  |  |  |  |  |
|  |  |  |  |  |  | **d) Adverse Birth Outcome** | **N/A** | N/A | | |  |  |  |  |  |  |  |  |
| **Money, 2020 - Infant Subset** | **3** | ***** | ***** | **5** | ***** | **a) Critical Care Indicators** | **N/A** | N/A | | |  |  |  |  |  |  |  |  |
|  |  |  |  |  |  | **b) Maternal Mortality & Morbidity** | **N/A** | N/A | | |  |  |  |  |  |  |  |  |
|  |  |  |  |  |  | **c) Fetal & Neonatal Mortality & Morbidity** | **N/A** | N/A | | |  |  |  |  |  |  |  |  |
|  |  |  |  |  |  | **d) Adverse Birth Outcome** | ***** | 4/6 stars | | |  |  |  |  |  |  |  |  |
| **Knight, 2021** | **3** | ***** | ***** | ***** | **4** | **a) Critical Care Indicators** | ***** | 4/5 stars | | |  |  |  |  |  |  |  |  |
|  |  |  |  |  |  | **b) Maternal Mortality & Morbidity** | ***** | 4/6 stars | | |  |  |  |  |  |  |  |  |
|  |  |  |  |  |  | **c) Fetal & Neonatal Mortality & Morbidity** | ***** | 4/6 stars | | |  |  |  |  |  |  |  |  |
|  |  |  |  |  |  | **d) Adverse Birth Outcome** | ***** | 4/6 stars | | |  |  |  |  |  |  |  |  |
| **Bracero, Valencia, Delgado-Lopez, 2021** | **7** | ***** | ***** | **5** | **4** | **a) Critical Care Indicators** | **8** | 2/5 stars | | |  |  |  |  |  |  |  |  |
|  |  |  |  |  |  | **b) Maternal Mortality & Morbidity** | **8** | 2/6 stars | | |  |  |  |  |  |  |  |  |
|  |  |  |  |  |  | **c) Fetal & Neonatal Mortality & Morbidity** | **8** | 2/6 stars | | |  |  |  |  |  |  |  |  |
|  |  |  |  |  |  | **d) Adverse Birth Outcome** | **8** | 2/6 stars | | |  |  |  |  |  |  |  |  |
| **Sakowicz 2020** | **7** | ***** | ***** | **5** | ***** | **a) Critical Care Indicators** | ***** | 3/5 stars | | |  |  |  |  |  |  |  |  |
|  |  |  |  |  |  | **b) Maternal Mortality & Morbidity** | ***** | 4/6 stars | | |  |  |  |  |  |  |  |  |
|  |  |  |  |  |  | **c) Fetal & Neonatal Mortality & Morbidity** | ***** | 4/6 stars | | |  |  |  |  |  |  |  |  |
|  |  |  |  |  |  | **d) Adverse Birth Outcome** | ***** | 4/6 stars | | |  |  |  |  |  |  |  |  |
| **Sanin, Mesa, Tolosa, 2021** | **7** | ***** | ***** | **5** | **4** | **a) Critical Care Indicators** | **8** | 2/5 stars | | |  |  |  |  |  |  |  |  |
|  |  |  |  |  |  | **b) Maternal Mortality & Morbidity** | **N/A** | N/A | | |  |  |  |  |  |  |  |  |
|  |  |  |  |  |  | **c) Fetal & Neonatal Mortality & Morbidity** | ***** | 3/6 stars | | |  |  |  |  |  |  |  |  |
|  |  |  |  |  |  | **d) Adverse Birth Outcome** | **N/A** | N/A | | |  |  |  |  |  |  |  |  |
| **Nachega 2021** | **7** | ***** | ***** | ***** | **4** | **a) Critical Care Indicators** | ***** | 4/5 stars | | |  |  |  |  |  |  |  |  |
|  |  |  |  |  |  | **b) Maternal Mortality & Morbidity** | ***** | 4/6 stars | | |  |  |  |  |  |  |  |  |
|  |  |  |  |  |  | **c) Fetal & Neonatal Mortality & Morbidity** | ***** | 4/6 stars | | |  |  |  |  |  |  |  |  |
|  |  |  |  |  |  | **d) Adverse Birth Outcome** | **8** | 3/6 stars | | |  |  |  |  |  |  |  |  |
| **Waldorf, Lokken, 2021** | **7** | ***** | ***** | ***** | **4** | **a) Critical Care Indicators** | ***** | 4/5 stars | | |  |  |  |  |  |  |  |  |
|  |  |  |  |  |  | **b) Maternal Mortality & Morbidity** | ***** | 4/6 stars | | |  |  |  |  |  |  |  |  |
|  |  |  |  |  |  | **c) Fetal & Neonatal Mortality & Morbidity** | ***** | 4/6 stars | | |  |  |  |  |  |  |  |  |
|  |  |  |  |  |  | **d) Adverse Birth Outcome** | ***** | 4/6 stars | | |  |  |  |  |  |  |  |  |
| **Divakar, 2021** | ***** | ***** | ***** | ***** | ***** | **a) Critical Care Indicators** | ***** | 5/5 stars | | |  |  |  |  |  |  |  |  |
|  |  |  |  |  |  | **b) Maternal Mortality & Morbidity** | ***** | 6/6 stars | | |  |  |  |  |  |  |  |  |
|  |  |  |  |  |  | **c) Fetal & Neonatal Mortality & Morbidity** | ***** | 6/6 stars | | |  |  |  |  |  |  |  |  |
|  |  |  |  |  |  | **d) Adverse Birth Outcome** | ***** | 6/6 stars | | |  |  |  |  |  |  |  |  |
| **Gil, Fernandez Buhigas, 2021** | ***** | ***** | ***** | ***** | ***** | **a) Critical Care Indicators** | ***** | 5/5 stars | | |  |  |  |  |  |  |  |  |
|  |  |  |  |  |  | **b) Maternal Mortality & Morbidity** | ***** | 6/6 stars | | |  |  |  |  |  |  |  |  |
|  |  |  |  |  |  | **c) Fetal & Neonatal Mortality & Morbidity** | ***** | 6/6 stars | | |  |  |  |  |  |  |  |  |
|  |  |  |  |  |  | **d) Adverse Birth Outcome** | ***** | 6/6 stars | | |  |  |  |  |  |  |  |  |
| **Crovetto 2020, Cohort I** | ***** | ***** | ***** | ***** | ***** | **a) Critical Care Indicators** | ***** | 5/5 stars | | |  |  |  |  |  |  |  |  |
|  |  |  |  |  |  | **b) Maternal Mortality & Morbidity** | ***** | 6/6 stars | | |  |  |  |  |  |  |  |  |
|  |  |  |  |  |  | **c) Fetal & Neonatal Mortality & Morbidity** | ***** | 6/6 stars | | |  |  |  |  |  |  |  |  |
|  |  |  |  |  |  | **d) Adverse Birth Outcome** | ***** | 6/6 stars | | |  |  |  |  |  |  |  |  |
| **Crovetto 2020, Cohort II** | ***** | ***** | ***** | ***** | ***** | **a) Critical Care Indicators** | ***** | 5/5 stars | | |  |  |  |  |  |  |  |  |
|  |  |  |  |  |  | **b) Maternal Mortality & Morbidity** | ***** | 6/6 stars | | |  |  |  |  |  |  |  |  |
|  |  |  |  |  |  | **c) Fetal & Neonatal Mortality & Morbidity** | ***** | 6/6 stars | | |  |  |  |  |  |  |  |  |
|  |  |  |  |  |  | **d) Adverse Birth Outcome** | ***** | 6/6 stars | | |  |  |  |  |  |  |  |  |
| **Bevilacqua, Laurita Longo, 2020** | ***** | ***** | ***** | ***** | ***** | **a) Critical Care Indicators** | ***** | 5/5 stars | | |  |  |  |  |  |  |  |  |
|  |  |  |  |  |  | **b) Maternal Mortality & Morbidity** | ***** | 6/6 stars | | |  |  |  |  |  |  |  |  |
|  |  |  |  |  |  | **c) Fetal & Neonatal Mortality & Morbidity** | ***** | 6/6 stars | | |  |  |  |  |  |  |  |  |
|  |  |  |  |  |  | **d) Adverse Birth Outcome** | ***** | 6/6 stars | | |  |  |  |  |  |  |  |  |
| **Nunes 2021** | **7** | ***** | ***** | ***** | ***** | **a) Critical Care Indicators** | **N/A** | N/A | | |  |  |  |  |  |  |  |  |
|  |  |  |  |  |  | **b) Maternal Mortality & Morbidity** | ***** | 5/6 stars | | |  |  |  |  |  |  |  |  |
|  |  |  |  |  |  | **c) Fetal & Neonatal Mortality & Morbidity** | ***** | 5/6 stars | | |  |  |  |  |  |  |  |  |
|  |  |  |  |  |  | **d) Adverse Birth Outcome** | ***** | 5/6 stars | | |  |  |  |  |  |  |  |  |
| **Akelo, Tippett Barr 2021** | ***** | ***** | ***** | ***** | **4** | **a) Critical Care Indicators** | **N/A** | N/A | | |  |  |  |  |  |  |  |  |
|  |  |  |  |  |  | **b) Maternal Mortality & Morbidity** | ***** | 5/6 stars | | |  |  |  |  |  |  |  |  |
|  |  |  |  |  |  | **c) Fetal & Neonatal Mortality & Morbidity** | ***** | 5/6 stars | | |  |  |  |  |  |  |  |  |
|  |  |  |  |  |  | **d) Adverse Birth Outcomes** | **8** | 4/6 stars | | |  |  |  |  |  |  |  |  |
| **Yang, Juan, 2020** | **7** | **9** | ***** | ***** | **4** | **a) Critical Care Indicators** | ***** | 3/5 stars | | |  |  |  |  |  |  |  |  |
|  |  |  |  |  |  | **b) Maternal Mortality & Morbidity** | ***** | 3/6 stars | | |  |  |  |  |  |  |  |  |
|  |  |  |  |  |  | **c) Fetal & Neonatal Mortality & Morbidity** | ***** | 3/6 stars | | |  |  |  |  |  |  |  |  |
|  |  |  |  |  |  | **d) Adverse Birth Outcome** | ***** | 3/6 stars | | |  |  |  |  |  |  |  |  |
| **Kalafat 2020** | **7** | ***** | ***** | ***** | ***** | **a) Critical Care Indicators** | ***** | 4/5 stars | | |  |  |  |  |  |  |  |  |
|  |  |  |  |  |  | **b) Maternal Mortality & Morbidity** | ***** | 5/6 stars | | |  |  |  |  |  |  |  |  |
|  |  |  |  |  |  | **c) Fetal & Neonatal Mortality & Morbidity** | ***** | 5/6 stars | | |  |  |  |  |  |  |  |  |
|  |  |  |  |  |  | **d) Adverse Birth Outcomes** | ***** | 5/6 stars | | |  |  |  |  |  |  |  |  |
| **Brandt 2020** | ***** | ***** | ***** | ***** | ***** | **a) Critical Care Indicators** | ***** | 5/5 stars | | |  |  |  |  |  |  |  |  |
|  |  |  |  |  |  | **b) Maternal Mortality & Morbidity** | ***** | 6/6 stars | | |  |  |  |  |  |  |  |  |
|  |  |  |  |  |  | **c) Fetal & Neonatal Mortality & Morbidity** | ***** | 6/6 stars | | |  |  |  |  |  |  |  |  |
|  |  |  |  |  |  | **d) Adverse Birth Outcome** | ***** | 6/6 stars | | |  |  |  |  |  |  |  |  |
| **Poon 2021** | **3** | ***** | ***** | ***** | ***** | **a) Critical Care Indicators** | ***** | 4/5 stars | | |  |  |  |  |  |  |  |  |
|  |  |  |  |  |  | **b) Maternal Mortality & Morbidity** | ***** | 5/6 stars | | |  |  |  |  |  |  |  |  |
|  |  |  |  |  |  | **c) Fetal & Neonatal Mortality & Morbidity** | ***** | 5/6 stars | | |  |  |  |  |  |  |  |  |
|  |  |  |  |  |  | **d) Adverse Birth Outcome** | ***** | 5/6 stars | | |  |  |  |  |  |  |  |  |
| Notes: This table presents risk of bias findings for each participating study based on criteria adapted from the Newcastle Ottawa scale (see Table S2). In the final column, we provide the number of stars  awarded to the study for each outcome group. Critical Care outcomes are scored on a scale of 0-5 (excluding the rate of follow-up through pregnancy outcome). All other outcome groups are scored  on a scale of 0-6. | | | | | | | | | | | | | | | | | |  |
|  |  |  |  |  |  |  |  |  |  |  |  |  |  |  |  |  |  |  |
| **1** See Table S19 for a full description of the percentage of missing data by risk factor and study site. | | | | | | | | | | | | | |  |  |  |  |  |
| **2** See Table S20 for a full description of the overall follow-up rate through pregnancy outcome and percentage of missing data by outcome group. | | | | | | | | | | | | | | |  |  |  |  |
| **3** Representativeness of the exposed cohort domain deemed at higher risk of bias because information regarding testing methods were unavailable. | | | | | | | | | | | | | | | |  |  |  |
| **4** Pregnancy follow up domain deemed at higher risk of bias because <90% of pregnancy outcomes had been ascertained at the time of data transfer | | | | | | | | | | | | | | | |  |  |  |
| **5** Risk factor data completeness domain deemed at higher risk of bias because half or more of the available risk factors had missing data for over 11 % of participants | | | | | | | | | | | | | | | |  |  |  |
| **6** Pregnancy follow up domain deemed at higher risk of bias because adequacy of follow up of cohorts for pregnancy outcome is unknown | | | | | | | | | | | | | | |  |  |  |  |
| **7** Representativeness of the exposed cohort domain deemed at higher risk of bias because 50% or more of the cases were identified using a method that was only somewhat representative of all  SARS-CoV-2-infected pregnant women in the community (e.g., pregnant women tested at antenatal care of delivery based on symptoms or travel; pregnant women tested for antibodies during routine  screening; medical records of pregnant women hospitalized for any reason, excluding delivery). | | | | | | | | | | | | | | | | | |  |
|  |  |  |  |  |  |  |  |  |  |  |  |  |  |  |  |  |  |  |
| **8** Data completeness domain deemed at higher risk of bias because one or more outcomes in this category had missing data for more than 10% of participants | | | | | | | | | | | | | | | |  |  |  |
| **9** Ascertainment of exposure (SARS-CoV-2 Infection) domain deemed at higher risk of bias because a proportion of Covid-19 positive cases were identified through clinical diagnosis or  radiography consistent with WHO case definitions of probably and suspected cases. | | | | | | | | | | | | | | | | | |  |
|  | | | | | | | | |  |  | |  |  |  |  |  |  |  |

Table S5. Detailed risk of bias of the selection of the exposed population and risk factor assessment for individual studies based on an adapted Newcastle Ottawa Scale

| **Study** | **Selection** | | **Exposure Assessment (Risk Factors)** | |  |
| --- | --- | --- | --- | --- | --- |
|  | **Representativeness of the population (SARS-CoV-2 Infected pregnant people)** | **Confirmation of SARS-CoV-2 Infection** | **Assessment of risk factor and outcome** | **Adequacy of Risk Factor Data 1** |  |
| **Martinez-Portilla, 2021** | All patients were screened in the hospital; reason for hospitalization (COVID-related, pregnancy-related, or other) is not available | Positive PCR test result | All data was collected from medical records by the epidemiologist of each of the 475 monitoring hospitals. The epidemiologist was in charge of acquiring the data from the medical records and uploading it to the national database. | 1) Comorbidities: <1% missing for diabetes, hypertension, and CVD; HIV not available  2) Nutritional Status: n/a 3) Maternal Age: 0% missing for maternal age 4) Symptomatic Status: n/a |  |
| **Favre, Panchaud, 2021** | Unavailable (screening policies vary by center and indication for test is not collected). | Positive PCR test result  or Positive antigen test result | Deidentified data were prospectively recorded by each center using the REDCap (Research Electronic Data Capture) electronic data capture tool. | 1) Comorbidities: 0% missing for diabetes, hypertension, and CVD; 34% missing for HIV 2) Nutritional Status: 71% missing for anemia; BMI n/a 3) Maternal Age: 0% missing for maternal age 4) Symptomatic Status: 0% missing |  |
| **Money, 2020 - Maternal Subset** | Unavailable (screening policies vary by province/facility and indication for test is not collected); however, the majority of cases were tested due to clinical concern. | Positive PCR test result | Information was collected from medical records | 1) Comorbidities: <1% missing for hypertension and CVD; 14% missing for diabetes; n/a for HIV coinfection 2) Nutritional Status: 62% missing for BMI; n/a for anemia 3) Maternal Age: <1% missing for maternal age (ICU/critical care); 14% missing for maternal age (ventilation) 4) Symptomatic Status: n/a |  |
| **Money, 2020 - Infant Subset** | All patients were hospitalized; reason for hospitalization (COVID-related, pregnancy-related, or other) is not available | Positive PCR test result (of mother) | Information was collected from medical records | 1) Comorbidities: 70% missing for hypertension, diabetes, and CVD; n/a for HIV coinfection 2) Nutritional Status: 79% missing for BMI; n/a for anemia 3) Maternal Age: 70% missing for maternal age 4) Symptomatic Status: n/a |  |
| **Carrillo, 2021** | Universal screening at delivery: 244 cases Universal screening at ANC: 240 cases Universal screening in postpartum: 2 cases Universal screening for abortion: 14 cases Testing for clinical concern: 837 Unknown: 10 | Positive PCR test result (99% of sample) or  Positive antibody test result or  CT Scan | Each center recorded the deidentifed data prospectively using the REDCap (Research Electronic Data Capture) electronic data capture tool. | 1) Comorbidities: 0% missing for diabetes, hypertension, and CVD; HIV not available  2) Nutritional Status: 21% missing for BMI; anemia not available 3) Maternal Age: 1% missing for maternal age 4) Symptomatic Status: 0% missing |  |
| **Knight, 2021** | All patients were hospitalized; reason for hospitalization (COVID-related, pregnancy-related, or other) is not available | Positive PCR test result | Medical records | 1) Comorbidities: 0% missing for diabetes, hypertension, and CVD; HIV not available  2) Nutritional Status: n/a 3) Maternal Age: 8% missing for maternal age 4) Symptomatic Status: 0% missing |  |
| **Bracero, Valencia, Delgado-Lopez, 2021** | Screening at delivery: 140 cases (14.9%)  Testing for clinical concern: 798 cases (85.1%) | Positive PCR test result | The primary data collection method is by medical record abstraction. Additional methods are database linkages with PR Vatial Signs (Birth certificate database) and with the PR Covid 19 General Surveillance (All positive cases in Puerto Rico database) | 1) Comorbidities: 12-14% missing for diabetes, hypertension; 89% missing for CVD; 34% missing for HIV  2) Nutritional Status: 30% missing for BMI; anemia not available 3) Maternal Age: 2% missing for maternal age 4 Symptomatic Status: 46% missing |  |
| **Sakowicz 2020** | (March 2020-Feb2021): Testing for "clinical concern" (357 cases, 71%)  (Mar 2020-Feb 2021): Universal testing for admission to the Labor & Delivery (146 cases, 29%) | Electronic health records (EHRs) indicating PCR positive test results | Electronic health records (EHRs) were reviewed for all pregnant women identified to have a SARS-CoV-2 test performed | 1) Comorbidities: 64% missing for diabetes, hypertension and CVD; n/a for HIV 2) Nutritional Status: n/a 3) Maternal Age: 0% missing for maternal age 4) Symptomatic Status: 2% missing |  |
| **Sanin, Mesa, Tolosa, 2021** | Testing for clinical concern: 200 cases (49%) Hospitalized for COVID-19: 209 cases (51%) | Positive PCR test result  or Positive antibody test result or Positive antigen test result or  Epidemiological, clinical, and radiographic findings confirm the infection | Electronic medical records | 1) Comorbidities: 20% missing for diabetes, hypertension, CVD and HIV 2) Nutritional Status: 74% missing for BMI; 48% missing for anemia 3) Maternal Age: n/a 4) Symptomatic Status: 21% missing |  |
| **Nachega 2021** | Symptomatic hospitalized women | Positive PCR test result | All patient data were abstracted from the patient hospital charts and log books | 1) Comorbidities: <3% missing for diabetes, hypertension, and HIV; 51% missing for CVD 2) Nutritional Status: 49% missing for anemia; n/a for BMI 3) Maternal Age: 1% missing 4) Symptomatic Status: 0% missing |  |
| **Waldorf, Lokken, 2021** | Universal screening at delivery & before medical procedures: 45 (18.8%)   Testing for clinical concern: 195 cases (81.2%) | Positive PCR test result | Deidentified data were abstracted from electronic medical records | 1) Comorbidities: 0% missing for diabetes, hypertension, and CVD; 8-10% missing for HIV 2) Nutritional Status: 6% missing for BMI; n/a for anemia 3) Maternal Age: 0% missing 4) Symptomatic Status: 0% missing |  |
| **Divakar, 2021** | Screening at delivery:181 cases (84%) Screening at ANC: 27 cases (12%) Hospitalized for COVID-19: 2 cases (1%) Testing for clinical concern: 2 cases (1%) | Positive PCR test result | The data was collected from the medical records after obtaining consent from the patients | 1) Comorbidities: 0% missing for diabetes, hypertension, and CVD; n/a for HIV 2) Nutritional Status: 83% missing for BMI; 55% missing for anemia 3) Maternal Age: 0% missing 4) Symptomatic Status: 0% missing |  |
| **Gil, Fernandez Buhigas, 2021** | Screening at ANC: 162 cases (76.42%) Universal serological screening performed by Torrejon city: 28 cases (13.21%) Admitted to hospital for delivery: 11 cases (5.19%) Admitted to hospital for COVID-19: 9 cases (4.24%) Universal testing for hospital admission other than Labor & delivery including surgery for miscarriage: 2 cases (0.94%) | Positive PCR test result  **or** Positive antibody test result **or** Positive antigen test result | Electronic medical records | 1) Comorbidities: 0% missing for diabetes, hypertension, and CVD, HIV 2) Nutritional Status: 2% missing for BMI; n/a for anemia 3) Maternal Age: 0% missing 4) Symptomatic Status: 0% missing |  |
| **Crovetto 2020, Cohort I** | Phase 1 (Initial recruitment from March-May 2020): Pregnant women at 10-16 weeks of gestation who provided blood sample for Down's syndrome screening were tested for SARS- CoV-2 antibodies (141 cases, 81.5%) Phase 2: Follow-up testing at labor and delivery for all phase 1 participants using PCR; 32 cases, 18.5%) | Tested positive for SARS-CoV-2 antibodies in early pregnancy  **OR** PCR positive at delivery | All patient data were abstracted from the electronic medical records or hospital records | 1) Comorbidities: 0% missing for diabetes, CVD; n/a for hypertension, HIV 2) Nutritional Status: 0% missing for BMI; n/a for anemia 3) Maternal Age: 0% missing 4) Symptomatic Status: 0% missing |  |
| **Crovetto 2020, Cohort II** | Universal testing at Labor & Delivery (Apr-May 2020): Universal testing (antibody testing for all, PCR testing for some) at Labor & Delivery  Tested positive with PCR test only: 3 cases (1.7%) Tested positive for antibody test only: 138 cases (78.4%) Tested positive with both PCR and antibody test: 35 cases (19.9%) | PCR positive test result **OR** Positive antibody test result **OR** Positive PCR and antibody test at delivery | All patient data were abstracted from the electronic medical records or hospital records | 1) Comorbidities: 0% missing for diabetes, CVD; n/a for hypertension, HIV 2) Nutritional Status: 0% missing for BMI; n/a for anemia 3) Maternal Age: 0% missing 4) Symptomatic Status: 0% missing |  |
| **Bevilacqua, Laurita Longo, 2020** | Testing for "clinical concern" 36 cases (22%) Universal testing for admission to the Labor & Delivery 108 cases (66.2%) Universal testing for hospital admission other than Labor & delivery, including pregnancy-related admission pre-term 55 cases (33.7%) | Positive PCR test result | Clinical data were routinely collected in real time in the patient’s electronic medical records. | 1) Comorbidities: 0% missing for diabetes, hypertension, and CVD; HIV n/a 2) Nutritional Status: 10% missing for BMI; anemia n/a 3) Maternal Age: 0% missing for maternal age 4) Symptomatic Status: 0% missing |  |
| **Nunes 2021** | All women presenting for antenatal care (or admitted during pregnancy) with covid-like symptoms (119 cases, 85.6%)  Screening of 10 asymptomatic women per day (19 cases, 13.7%) Retested at labor & delivery after initial recruitment during antenatal care (1 case, 0.7%) | Positive PCR test result | All patient data were abstracted from the hospital records or telephonic contact | 1) Comorbidities: 0% missing for diabetes, hypertension, and CVD; 3% missing for HIV 2) Nutritional Status: n/a 3) Maternal Age: 0% missing for maternal age 4) Symptomatic Status: 1% missing |  |
| **Akelo, Tippett Barr 2021** | Population-based pregnancy surveillance: Universal screening at antenatal care, after enrollment (50 cases, 40.0%)  Universal screening at delivery (11 cases, 8.8%)  Universal screening at enrollment (32 cases, 25.6%)  Hospitalized for COVID-19 (1 cases diagnosed while hospitalized and 11 hospitalized for COVID-19 management, 9.6%) Other COVID-19 testing for clinical concern (1 case, 0.8%) Universal screening at postpartum visits (19 cases, 15.2%) | Positive PCR test result | Clinical data were routinely collected in real time based on clinic and home visits | 1) Comorbidities: 9% missing for diabetes, hypertension; n/a for CVD, HIV 2) Nutritional Status: n/a 3) Maternal Age: 0% missing for maternal age 4) Symptomatic Status: 3% missing |  |
| **Yang, Juan, 2020** | Hospitalized for COVID-19: 100% | Positive PCR test result  **or** Probable COVID-19 (clinical diagnosis or based on radiography) | Electronic medical records | 1) Comorbidities: n/a 2) Nutritional Status: n/a 3) Maternal Age: 0% missing for maternal age 4) Symptomatic Status: 0% missing |  |
| **Kalafat 2020** | Asymptomatic pregnant women admitted for delivery (19 cases, 24.7%) Symptomatic pregnant women evaluated for probable SARS-CoV-2 infection (58 cases, 75.3%) | Positive PCR test result | All patient data were abstracted from the patient hospital charts, logbooks and electronic patient records. | 1) Comorbidities: 0% missing for diabetes, hypertension, CVD, HIV 2) Nutritional Status: n/a 3) Maternal Age: 0% missing for maternal age 4) Symptomatic Status: 0% missing |  |
| **Brandt 2020** | (Mar-Apr 2020): Testing for "clinical concern", recent travel, or exposure to known case (11 cases, 18.0%) (Apr-Jun 2020): Universal testing for admission to the Labor & Delivery (50 cases, 82.0%) | Positive PCR test result | All patient data were abstracted from the electronic medical records | 1) Comorbidities: 0% missing for diabetes, hypertension; n/a for CVD, HIV 2) Nutritional Status: n/a 3) Maternal Age: 0% missing for maternal age 4) Symptomatic Status: 0% missing |  |
| **Poon 2021** | Testing for "clinical concern"  Universal testing for admission to the delivery  Universal testing for any hospital admission | Positive PCR test result | Electronic health records and clinical report | 1) Comorbidities: 0% missing for diabetes, hypertension, CVD, HIV 2) Nutritional Status: 64% missing for anemia; BMI n/a 3) Maternal Age: 0% missing for maternal age 4) Symptomatic Status: 0% missing |  |
| Notes: This table presents risk of bias findings for each participating study based on criteria adapted from the Newcastle Ottawa scale (see Table S2). In the final column, we provide the number of stars awarded to the study for each outcome group. Critical Care outcomes are scored on a scale of 0-5 (excluding the rate of follow-up through pregnancy outcome). All other outcome groups are scored on a scale of 0-6.  **1** See Table S19 for a full description of the percentage of missing data by risk factor and study site. | | | | |  |
|  |  |  |  |  |  |
|  |  |  |  |  |  |

Table S6. Detailed risk of bias assessment related to outcome assessment for individual studies based on an adapted Newcastle Ottawa Scale

| **Study** | **Outcome Assessment ^1^** | | **Total (*)** |  |
| --- | --- | --- | --- | --- |
|  | **Adequacy of follow up of cohorts for pregnancy outcome** | **Adequacy of data completeness** |  |  |
| **Martinez-Portilla, 2021** | Number of pregnancies with pregnancy outcome known: 0 (0%)   Note: Mexico National Registry does not include follow-up of pregnant people or pregnancy-specific outcomes. | a) Critical Care Indicators: <1% missing b) Maternal Mortality & Morbidity: <1% missing for maternal mortality; maternal morbidity not available c) Fetal & Neonatal Mortality & Morbidity: not available d) Adverse Birth Outcomes: not available | a) Critical Care Indicators: 4/5 b) Maternal Mortality & Morbidity: N/A c) Fetal & Neonatal Mortality & Morbidity: N/A d) Adverse Birth Outcomes: N/A |  |
| **Favre, Panchaud, 2021** | Number of pregnancies with pregnancy outcome known: 1,870 (78.2%) | a) Critical Care Indicators*: <1% missing for ICU admission, ventilation, critical care; 7% missing for pneumonia b) Maternal Mortality* & Morbidity: <3% missing c) Fetal & Neonatal Mortality & Morbidity: <4% missing d) Adverse Birth Outcomes: <8% missing  *Note: critical care and mortality indicators are only collected for inpatient participants | a) Critical Care Indicators: 4/5 b) Maternal Mortality & Morbidity: 4/6 c) Fetal & Neonatal Mortality & Morbidity: 4/6 d) Adverse Birth Outcomes: 4/6 |  |
| **Money, 2020 - Maternal Subset** | Number of pregnancies with pregnancy outcome known: Unknown (maternal COVID-19 outcome subset of data only) | a) Critical Care Indicators: <1% missing b) Maternal Mortality & Morbidity: not available c) Fetal & Neonatal Mortality & Morbidity: not available d) Adverse Birth Outcomes: not available | a) Critical Care Indicators: 3/5 b) Maternal Mortality & Morbidity: N/A c) Fetal & Neonatal Mortality & Morbidity: N/A d) Adverse Birth Outcomes: N/A |  |
| **Money, 2020 - Infant Subset** | Number of pregnancies with pregnancy outcome known: 2626 (100.0%)  Note: This subset of data represents liveborn infants born to mothers with Covid-19 only. | a) Critical Care Indicators: not available b) Maternal Mortality & Morbidity: not available c) Fetal & Neonatal Mortality & Morbidity: not available d) Adverse Birth Outcomes: <1% missing | a) Critical Care Indicators: N/A b) Maternal Mortality & Morbidity: N/A c) Fetal & Neonatal Mortality & Morbidity: N/A d) Adverse Birth Outcomes: 4/6 |  |
| **Carrillo, 2021** | Number of pregnancies with pregnancy outcome known: 1,127 (83.7%) | a) Critical Care Indicators: <5% missing b) Maternal Mortality & Morbidity: <2% missing c) Fetal & Neonatal Mortality & Morbidity: <1% missing, except NICU admission (11% missing)  d) Adverse Birth Outcomes: <10% missing | a) Critical Care Indicators: 4/5 b) Maternal Mortality & Morbidity: 4/6 c) Fetal & Neonatal Mortality & Morbidity: 3/6 d) Adverse Birth Outcomes: 4/6 |  |
| **Knight, 2021** | Number of pregnancies with pregnancy outcome known: 1,061 (85.4%) | a) Critical Care Indicators: <6% missing b) Maternal Mortality & Morbidity: 0% missing for pregnancy-related death; maternal morbidities n/a c) Fetal & Neonatal Mortality & Morbidity: <3% missing; NICU admission not available d) Adverse Birth Outcomes: <3% missing; low birthweight not available | a) Critical Care Indicators: 4/5 b) Maternal Mortality & Morbidity: 4/6 c) Fetal & Neonatal Mortality & Morbidity: 4/6 d) Adverse Birth Outcomes: 4/6 |  |
| **Bracero, Valencia, Delgado-Lopez, 2021** | Number of pregnancies with pregnancy outcome known: 754 (80.4%) | a) Critical Care Indicators: excluded (89-90% missing) b) Maternal Mortality & Morbidity: excluded (25% or more missing) c) Fetal & Neonatal Mortality & Morbidity: stillbirth - 0% missing; other outcomes excluded (32-33% missing) d) Adverse Birth Outcomes: excluded (25% or more missing) | a) Critical Care Indicators: 2/5 b) Maternal Mortality & Morbidity: 2/6 c) Fetal & Neonatal Mortality & Morbidity: 2/6 d) Adverse Birth Outcomes: 2/6 |  |
| **Sakowicz 2020** | Number of pregnancies with pregnancy outcome known: 503 (100%) | a) Critical Care Indicators: <1% missing b) Maternal Mortality & Morbidity: <1% missing c) Fetal & Neonatal Mortality & Morbidity: 0% missing for stillbirth; neonatal death and NICU admission excluded due to 68% missing data d) Adverse Birth Outcomes: <1% missing | a) Critical Care Indicators: 3/5 stars  b) Maternal Mortality & Morbidity: 4/6 stars  c) Fetal & Neonatal Mortality & Morbidity: 4/6 stars  d) Adverse Birth Outcomes: 4/6 stars |  |
| **Sanin, Mesa, Tolosa, 2021** | Number of pregnancies with pregnancy outcome known: 213 (52.5%) | a) Critical Care Indicators: ICU admission 21% missing; pneumonia and pregnancy-related death excluded (25-27% missing) b) Maternal Mortality & Morbidity: Haemorrhage 0% missing; cesarean delivery 23% missing, other outcomes excluded (27-28% missing) c) Fetal & Neonatal Mortality & Morbidity: Stillbirth 0% missing; other outcomes excluded (32-33% missing) d) Adverse Birth Outcomes: excluded (27-28% missing) | a) Critical Care Indicators: 2/5 b) Maternal Mortality & Morbidity: N/A c) Fetal & Neonatal Mortality & Morbidity: 3/6 d) Adverse Birth Outcomes: N/A |  |
| **Nachega 2021** | Number of pregnancies with pregnancy outcome known: 170 (48.7%) | a) Critical Care Indicators: 3%, except pneumonia (excluded due to 27% missing data) b) Maternal Mortality & Morbidity: <8%, except c-section (excluded due to 26% missing data) and preterm labor (excluded due to 33% missing data) c) Fetal & Neonatal Mortality & Morbidity: <3% d) Adverse Birth Outcomes: <20% | a) Critical Care Indicators: 4/5 stars  b) Maternal Mortality & Morbidity: 4/6 stars c) Fetal & Neonatal Mortality & Morbidity: 4/6 stars d) Adverse Birth Outcomes: 3/6 stars |  |
| **Waldorf, Lokken, 2021** | Number of pregnancies with pregnancy outcome known: 158 (65.8%) | a) Critical Care Indicators: <1% missing b) Maternal Mortality & Morbidity: <7% missing c) Fetal & Neonatal Mortality & Morbidity: <1% missing d) Adverse Birth Outcomes: <2% missing | a) Critical Care Indicators: 4/5 b) Maternal Mortality & Morbidity: 4/6  c) Fetal & Neonatal Mortality & Morbidity: 4/6  d) Adverse Birth Outcomes: 4/6 |  |
| **Divakar, 2021** | Number of pregnancies with pregnancy outcome known: 212 (100%) | a) Critical Care Indicators: <1% missing (ventilation only) b) Maternal Mortality & Morbidity: <1% missing, except for cesarean delivery (9% missing) c) Fetal & Neonatal Mortality & Morbidity: <1% missing d) Adverse Birth Outcomes: <2% missing | a) Critical Care Indicators: 5/5 b) Maternal Mortality & Morbidity: 6/6  c) Fetal & Neonatal Mortality & Morbidity: 6/6  d) Adverse Birth Outcomes: 6/6 |  |
| **Gil, Fernandez Buhigas, 2021** | Number of pregnancies with pregnancy outcome known: 172 (81.1%)  Note: This is an ongoing study and some missing pregnancy outcomes are because women have not yet delivered. The percentage of pregnancies with a recorded endpoint among those with expected due dates 4 weeks or more before the date data was shared is **100%.** | a) Critical Care Indicators: <2% missing b) Maternal Mortality & Morbidity: <4% missing c) Fetal & Neonatal Mortality & Morbidity: <2% missing d) Adverse Birth Outcomes: <3% missing | a) Critical Care Indicators: 5/5 b) Maternal Mortality & Morbidity: 6/6  c) Fetal & Neonatal Mortality & Morbidity: 6/6  d) Adverse Birth Outcomes: 6/6 |  |
| **Crovetto 2020, Cohort I** | Number of pregnancies with pregnancy outcome known: 159 (91.9%) | a) Critical Care Indicators: 0% missing  b) Maternal Mortality & Morbidity: <4% missing for all  c) Fetal & Neonatal Mortality & Morbidity: <1% missing, except NICU (where 10% is missing)  d) Adverse Birth Outcomes: <5% missing | a) Critical Care Indicators: 5/5 stars b) Maternal Mortality & Morbidity: 6/6 stars  c) Fetal & Neonatal Mortality & Morbidity: 6/6 stars  d) Adverse Birth Outcomes: 6/6 stars |  |
| **Crovetto 2020, Cohort II** | Number of pregnancies with pregnancy outcome known: 176 (100%) | a) Critical Care Indicators: 0% missing  b) Maternal Mortality & Morbidity: 0% missing  c) Fetal & Neonatal Mortality & Morbidity: < 5% missing  d) Adverse Birth Outcomes: 0% missing | a) Critical Care Indicators: 5/5 stars b) Maternal Mortality & Morbidity: 6/6 stars  c) Fetal & Neonatal Mortality & Morbidity: 6/6 stars  d) Adverse Birth Outcomes: 6/6 stars |  |
| **Bevilacqua, Laurita Longo, 2020** | Number of pregnancies with pregnancy outcome known: 163 (100%) | a) Critical Care Indicators: 0% missing b) Maternal Mortality & Morbidity: 0% missing, except c-section (3%) c) Fetal & Neonatal Mortality & Morbidity: 0% missing d) Adverse Birth Outcomes: <1% missing | a) Critical Care Indicators: 5/5 stars b) Maternal Mortality & Morbidity: 6/6 stars  c) Fetal & Neonatal Mortality & Morbidity: 6/6 stars  d) Adverse Birth Outcomes: 6/6 stars |  |
| **Nunes 2021** | Number of pregnancies with pregnancy outcome known: 133 (95.7%) | a) Critical Care Indicators: Data not available b) Maternal Mortality & Morbidity: <2% missing c) Fetal & Neonatal Mortality & Morbidity: 0% missing (NICU admission not available) d) Adverse Birth Outcomes: <5% missing | a) Critical Care Indicators: N/A b) Maternal Mortality & Morbidity: 5/6 stars  c) Fetal & Neonatal Mortality & Morbidity: 5/6 stars  d) Adverse Birth Outcomes: 5/6 stars |  |
| **Akelo, Tippett Barr 2021** | Number of pregnancies with pregnancy outcome known: 93 (74.4%)  Note: This is an ongoing cohort study and some missing pregnancy outcomes are because women have not yet delivered. The percentage of pregnancies (including both COVID-positive and COVID-negative observations) with a recorded endpoint among those with expected due dates 4 weeks or more before the date data was shared is **83%.** | a) Critical Care Indicators: Data excluded due to missingness >25% b) Maternal Mortality & Morbidity: 0% Missing c) Fetal & Neonatal Mortality & Morbidity: 0% missing for stillbirth; neonatal follow up in progress still and we thus exclude other measures of neonatal mortality (>25% missing). d) Adverse Birth Outcomes: Missing 7% preterm, 7% birthweight, 19% SGA | a) Critical Care Indicators: N/A b) Maternal Mortality & Morbidity: 5/6 stars  c) Fetal & Neonatal Mortality & Morbidity: 5/6 d) Adverse Birth Outcomes: 4/6 stars |  |
| **Yang, Juan, 2020** | Number of pregnancies with pregnancy outcome known: 100 (86.2%) | a) Critical Care Indicators: <1% missing b) Maternal Mortality & Morbidity: <1% missing c) Fetal & Neonatal Mortality & Morbidity: <2% missing d) Adverse Birth Outcomes: <3% missing | a) Critical Care Indicators: 3/5 stars  b) Maternal Mortality & Morbidity: 3/6 stars  c) Fetal & Neonatal Mortality & Morbidity: 3/6 stars d) Adverse Birth Outcomes: 3/6 stars |  |
| **Kalafat 2020** | Number of pregnancies with pregnancy outcome known: 74 (96.1%) | a) Critical Care Indicators: 0% missing b) Maternal Mortality & Morbidity: 0% missing c) Fetal & Neonatal Mortality & Morbidity: 1% missing d) Adverse Birth Outcomes: 0% missing | a) Critical Care Indicators: 4/5 stars  b) Maternal Mortality & Morbidity: 5/6 stars  c) Fetal & Neonatal Mortality & Morbidity: 5/6 stars d) Adverse Birth Outcomes: 5/6 stars |  |
| **Brandt 2020** | Number of pregnancies with pregnancy outcome known: 61 (100%) | a) Critical Care Indicators: 0% missing b) Maternal Mortality & Morbidity: 0% missing c) Fetal & Neonatal Mortality & Morbidity: 0% missing d) Adverse Birth Outcomes: 0% missing | a) Critical Care Indicators: 5/5 stars b) Maternal Mortality & Morbidity: 6/6 stars  c) Fetal & Neonatal Mortality & Morbidity: 6/6 stars  d) Adverse Birth Outcomes: 6/6 stars |  |
| **Poon 2021** | Number of pregnancies with pregnancy outcome known: 25 (100%) | a) Critical Care Indicators: 0% missing b) Maternal Mortality & Morbidity: 0% missing c) Fetal & Neonatal Mortality & Morbidity: 0% missing d) Adverse Birth Outcomes: 0% missing | a) Critical Care Indicators: 4/5 stars  b) Maternal Mortality & Morbidity: 5/6 stars  c) Fetal & Neonatal Mortality & Morbidity: 5/6 stars  d) Adverse Birth Outcomes: 5/6 stars |  |
| Notes: This table presents risk of bias findings for each participating study based on criteria adapted from the Newcastle Ottawa scale (see Table S2). In the final column, we provide the number of stars awarded to the study for each outcome group. Critical Care outcomes are scored on a scale of 0-5 (excluding the rate of follow-up through pregnancy outcome). All other outcome groups are scored on a scale of 0-6.  **1** See Table S20 for a full description of the overall follow-up rate through pregnancy outcome and percentage of missing data by outcome group. | | | |  |
|  |  |  |  |  |

Table S7. Risk of Outcomes among COVID+ pregnancy, stratified by chronic diabetes

| Outcome | N Studies | Included Studies **^1^** | With Chronic Diabetes | | Without Chronic Diabetes | | Pooled, unadjusted RR (95% CI) | I^2 (pvalue) |  |  |  |
| --- | --- | --- | --- | --- | --- | --- | --- | --- | --- | --- | --- |
|  |  |  | Events/Total | Pooled Risk (95% CI) | Events/Total | Pooled Risk (95% CI) |  |  |  |  |  |
|  |  |  |  |  |  |  |  |  |  |  |  |
| ICU admission | 16 | a b c1 d e g h i j l* m1* m2 n r s t | 49 / 583 | 0.09 (0.03, 0.21) | 635 / 18777 | 0.03 (0.02, 0.06) | **2.55 (1.97, 3.31)** | 0 (0.56) |  |  |  |
| Ventilation | 14 | a b c1 d e i j l m1* m2 n r s t | 23 / 568 | 0.04 (0.01, 0.14) | 265 / 18497 | 0.01 (0.01, 0.02) | **5.88 (2.77, 12.48)** | 51.49 (0.02) |  |  |  |
| Critical Care | 14 | a b c1 d e i j l m1* m2 n r s t* | 47 / 567 | 0.09 (0.04, 0.20) | 621 / 18473 | 0.03 (0.02, 0.05) | **3.03 (1.86, 4.92)** | 44.37 (0.05) |  |  |  |
| Pneumonia | 10 | a b d e l m1* m2 n r t* | 83 / 475 | 0.18 (0.06, 0.44) | 1240 / 16152 | 0.08 (0.03, 0.17) | **2.02 (1.65, 2.47)** | 0 (0.93) |  |  |  |
| Pregnancy-related death | 15 | a b d e h i j l* m1* m2* n* o r* s* t* | 27 / 477 | 5413.51 (1393.10, 18821.88) | 191 / 15228 | 1716.82 (623.52, 4637.72) | **3.79 (2.61, 5.50)** | 0 (0.70) |  |  |  |
|  |  |  |  |  |  |  |  |  |  |  |  |
| Haemorrhage | 7 | g h i j l n o | 7 / 38 | 0.18 (0.09, 0.34) | 105 / 1105 | 0.07 (0.04, 0.14) | 1.89 (0.96, 3.70) | 0 (0.49) |  |  |  |
| Placental Abruption | 6 | g i j l r* t | 2 / 28 | 0.07 (0.02, 0.24) | 11 / 678 | 0.02 (0.01, 0.03) | **7.25 (2.47, 21.25)** | 0 (0.69) |  |  |  |
| Preeclampsia | 10 | b d g l m1 m2 o r s t | 8 / 90 | 0.07 (0.01, 0.29) | 156 / 3812 | 0.05 (0.03, 0.07) | **2.98 (1.61, 5.51)** | 0 (0.55) |  |  |  |
| Preeclampsia or Eclampsia | 7 | g i m1 m2 n o t | 11 / 35 | 0.30 (0.10, 0.61) | 81 / 972 | 0.06 (0.03, 0.12) | **4.32 (1.58, 11.84)** | 50.55 (0.06) |  |  |  |
| Hypertensive Disorders of Pregnancy (Any) | 9 | d g i l m1 m2 n o t | 23 / 67 | 0.37 (0.21, 0.55) | 218 / 2227 | 0.09 (0.05, 0.14) | **2.73 (1.62, 4.58)** | 30.14 (0.18) |  |  |  |
| Hypertensive Disorders of Pregnancy (At/After Covid-19) | 2 | l t | -- | -- | -- | -- | -- | -- |  |  |  |
| Preterm labor | 8 | d j l m1* m2 n o t | 8 / 50 | 0.16 (0.08, 0.29) | 100 / 1892 | 0.05 (0.04, 0.07) | **3.54 (1.89, 6.61)** | 0 (0.73) |  |  |  |
| Preterm labor with onset before 37w GA **^2^** | 6 | d j l n o t | 6 / 33 | 0.17 (0.02, 0.38) | 85 / 959 | 0.08 (0.06, 0.11) | **2.48 (1.24, 4.98)** | 0 (0.88) |  |  |  |
| Cesarean Delivery | 12 | b d g h j l m1 m2 n o s t | 53 / 94 | 0.55 (0.41, 0.69) | 1448 / 4085 | 0.34 (0.28, 0.40) | **1.40 (1.13, 1.74)** | 0 (0.96) |  |  |  |
| Intrapartum Cesarean Delivery | 9 | b d j l m1* m2 n o t | 22 / 74 | 0.30 (0.20, 0.42) | 742 / 3571 | 0.20 (0.15, 0.26) | 1.30 (0.90, 1.87) | 0 (0.89) |  |  |  |
|  |  |  |  |  |  |  |  |  |  |  |  |
| Stillbirth **^3^** | 16 | b d e f g* h* i j l* m1 m2* n* o r s* t* | 1 / 121 | 8.29 (1.17, 56.29) | 37 / 5136 | 8.37 (4.61, 15.16) | **6.53 (2.13, 20.05)** | 0 (0.87) |  |  |  |
| Perinatal death | 12 | b d e j l* m1 m2 n* o r s* t* | 0 / 103 | *incalculable* | 40 / 4531 | 8.83 (6.48, 12.01) | **7.71 (2.12, 28.03)** | 0 (0.75) |  |  |  |
| Early neonatal death | 12 | b d e j* l* m1* m2 n* o* r* s* t* | 0 / 82 | *incalculable* | 13 / 4006 | 2.68 (1.11, 4.76) | **6.97 (1.07, 45.27)** | 0 (0.66) |  |  |  |
| Neonatal death **^4^** | 13 | b d e i j* l* m1* m2 n* o* r* s* t* | 0 / 88 | *incalculable* | 18 / 4136 | 4.35 (2.74, 6.90) | **6.85 (1.22, 38.49)** | 0 (0.69) |  |  |  |
| NICU Admission at Birth | 8 | b d m1 m2 n r s t | 17 / 73 | 0.34 (0.10, 0.70) | 336 / 3447 | 0.11 (0.04, 0.31) | **1.83 (1.15, 2.93)** | 14.55 (0.32) |  |  |  |
|  |  |  |  |  |  |  |  |  |  |  |  |
| Very low birthweight (<1500g) | 13 | b d g i j* l m1 m2 n o r* s t* | 6 / 93 | 0.06 (0.03, 0.14) | 80 / 3958 | 0.02 (0.02, 0.03) | **5.28 (2.62, 10.63)** | 0 (0.59) |  |  |  |
| Low birthweight (<2500g) | 13 | b d g i j l m1 m2 n o r s t | 20 / 102 | 0.20 (0.13, 0.28) | 468 / 4201 | 0.11 (0.08, 0.15) | **1.80 (1.21, 2.69)** | 0 (0.87) |  |  |  |
| Small for gestational age (3rd) | 14 | b d e g i j* l m1 m2 n o r s* t | 1 / 112 | 0.01 (0.00, 0.06) | 116 / 5010 | 0.03 (0.02, 0.04) | **4.11 (1.53, 11.06)** | 0 (0.71) |  |  |  |
| Small for gestational age (10th) | 14 | b d e g i j l m1 m2 n o r s t | 6 / 126 | 0.04 (0.01, 0.13) | 376 / 5211 | 0.08 (0.06, 0.10) | 1.62 (0.81, 3.21) | 0 (0.94) |  |  |  |
| Moderate preterm birth (<34w) | 14 | b d e g i j l m1 m2 n o r s t | 17 / 126 | 0.13 (0.08, 0.22) | 248 / 5211 | 0.04 (0.03, 0.06) | **3.23 (2.09, 5.01)** | 0 (0.84) |  |  |  |
| Moderate preterm birth (<34w) with onset before 34w GA **^2^** | 8 | b d g j n o s t | 12 / 49 | 0.21 (0.07, 0.49) | 156 / 1581 | 0.11 (0.08, 0.16) | **2.03 (1.24, 3.31)** | 0 (0.94) |  |  |  |
| Preterm birth (<37 wks) | 15 | b c2 d e g i j l m1 m2 n o r s t | 53 / 145 | 0.37 (0.29, 0.45) | 808 / 5975 | 0.12 (0.09, 0.17) | **2.25 (1.77, 2.86)** | 0 (0.77) |  |  |  |
| Preterm birth (<37 wks) with onset before 37w GA **^2^** | 8 | b d g j n o s t | 23 / 60 | 0.39 (0.25, 0.54) | 436 / 2117 | 0.22 (0.17, 0.29) | 1.40 (0.97, 2.01) | 0 (1.00) |  |  |  |
| *Notes: Pooled absolute risks are calculated using a logistic-normal random-effects meta-analysis, pooling all participating studies with at least 1 adverse event for the given outcome; in cases*  *where the model does not converge, we instead calculate pooled absolute risk using a random effects model with Freeman-Tukey Double Arcsine Transformation. For any study with zero*  *events in one arm at the study level (Risk Group or Reference Group), we apply a continuity correction of 0.5 for pooled absolute risks. For any outcome with zero events for all studies in a*  *given risk or reference group, we consider the absolute risk to be incalculable based on the current data. Relative risks are calculated by pooling unadjusted relative risks from all participating*  *studies with at least 1 adverse event for the given outcome using a DerSimonian-Laird random effects model meta-analysis. For any study with zero events in one arm (Risk Group or*  *Reference Group), we used a continuity correction of the inverse of the number of events in the oppposite group within the same study. We present pooled estimates for analyses where at*  *least three studies report the relevant data and report at least 1 adverse event for the outcome of interest.  1 This column lists the studies that provide data for the given estimate. Any study sites indicated with an asterisk (*) reported "0" events in among those with and without the given risk factor.*  *These "0 event" studies are excluded from the estimate and from the "Events/Total" column. Studies are identified as follows: a) Martinez-Portilla, 2021, Mexico; b) Favre, Panchaud, 2021,*  *multicountry COVI-Preg registry; c1) Money, 2020 – Maternal Subset, Canada; c2) Money, 2020 – Infant Subset, Canada; d) Carrillo, 2021, Chile; e) Knight, 2021, United Kingdom; f) Bracero,*  *Valencia, Delgado-Lopez, 2021, Puerto Rico (USA); g) Sakowicz, 2020, USA (Chicago); h) Sanin, Mesa, Tolosa, 2021, Colombia; i) Nachega, 2021, multicountry AFREHealth study; j) Waldorf,*  *Lokken, 2021, USA (Washington State); k) Divakar, 2021, India (Karnataka State); l) Gil, Fernandez Buhigas, 2021, Spain (Madrid); m1) Crovetto, 2020, Cohort I, Spain (Barcelona);*  *m2) Crovetto,2020, Cohort II, Spain (Barcelona); n) Bevilacqua, Laurita Longo, 2020, Italy (Rome); o) Nunes, 2021, South Africa; p) Akelo, Tippett Barr, 2021, Kenya; q) Yang, Juan, 2020, China;*  *r) Kalafat, 2020, Turkey; s) Brandt, 2020, USA (New Brunswick); t) Poon, 2021, Hong Kong (China). 2 These outcomes (preterm labor, moderate preterm birth before 34 weeks gestation, and preterm birth before 37 weeks’ gestation) were included in the sensitivity analyses where we restrict*  *confirmed COVID-19 cases to those with confirmed COVID-19 onset prior to 37 weeks’ gestation (or 34 weeks for very moderate preterm birth). The full comparison group is used for each of*  *the sensitivity analyses.  3 The outcome presented here is stillbirths occurring at or after 28 weeks gestational age per the WHO definition.  4 The outcome "neonatal death" is reported by 15 participating studies. However, most studies were not designed to follow-up neonates until 28 days after birth. Therefore, counts of neonatal*  *death are underestimated.* | | | | | | | | | | |  |
|  |  |  |  |  |  |  |  |  |  |  |  |
|  |  |  |  |  |  |  |  |  |  |  |  |
|  |  |  |  |  |  |  |  |  |  |  |  |
|  |  |  |  |  |  |  |  |  |  |  |  |
|  |  |  |  |  |  |  |  |  |  |  |  |
|  |  |  |  |  |  |  |  |  |  |  |  |
|  |  |  |  |  |  |  |  |  |  |  |  |
|  |  |  |  |  |  |  |  |  |  |  |  |
|  |  |  |  |  |  |  |  |  |  |  |  |
|  |  |  |  |  |  |  |  |  |  |  |  |
|  |  |  |  |  |  |  |  |  |  |  |  |
|  |  |  |  |  |  |  |  |  |  |  |  |
|  |  |  |  |  |  |  |  |  |  |  |  |
|  |  |  |  |  |  |  |  |  |  |  |  |
|  |  |  |  |  |  |  |  |  |  |  |  |

Table S8. Risk of Outcomes among COVID+ pregnancy, stratified by chronic hypertension

| Outcome | N Studies | Included Studies **^1^** | With Chronic Hypertension | | Without Chronic Hypertension | | Pooled, unadjusted RR (95% CI) | I^2 (pvalue) |  |
| --- | --- | --- | --- | --- | --- | --- | --- | --- | --- |
|  |  |  | Events/Total | Pooled Risk (95% CI) | Events/Total | Pooled Risk (95% CI) |  |  |  |
|  |  |  |  |  |  |  |  |  |  |
| ICU admission | 14 | a b c1 d e g h i j l* n r s t | 57 / 608 | 0.10 (0.05, 0.18) | 633 / 18846 | 0.04 (0.02, 0.06) | **2.10 (1.63, 2.70)** | 0 (0.54) |  |
| Ventilation | 13 | a b c1 d e i j k l n r s t | 29 / 571 | 0.05 (0.02, 0.11) | 259 / 18799 | 0.01 (0.01, 0.02) | **4.87 (2.93, 8.09)** | 22.07 (0.22) |  |
| Critical Care | 12 | a b c1 d e i j l n r s t* | 54 / 566 | 0.10 (0.06, 0.18) | 619 / 18567 | 0.03 (0.02, 0.05) | **2.42 (1.73, 3.39)** | 20.50 (0.25) |  |
| Pneumonia | 8 | a b d e l n r t* | 84 / 454 | 0.19 (0.08, 0.36) | 1232 / 15999 | 0.08 (0.03, 0.20) | **2.13 (1.74, 2.61)** | 0 (0.96) |  |
| Pregnancy-related death | 14 | a b d e h i j k* l* n* o r* s* t* | 29 / 494 | 5168.83 (1671.25, 14878.50) | 188 / 15211 | 1629.71 (599.81, 4350.63) | **2.75 (1.76, 4.28)** | 12.06 (0.34) |  |
|  |  |  |  |  |  |  |  |  |  |
| Haemorrhage | 7 | g h i j l n o | 9 / 88 | 0.10 (0.04, 0.21) | 102 / 1054 | 0.08 (0.04, 0.15) | 1.33 (0.60, 2.94) | 19.48 (0.28) |  |
| Placental Abruption | 6 | g i j l r* t | 4 / 60 | 0.04 (0.00, 0.43) | 9 / 645 | 0.01 (0.01, 0.03) | **6.68 (2.35, 18.98)** | 0 (0.60) |  |
| Preeclampsia | 9 | b d g k l o r s t | 37 / 129 | 0.30 (0.20, 0.42) | 136 / 3649 | 0.04 (0.03, 0.07) | **5.80 (4.11, 8.19)** | 0 (0.59) |  |
| Preeclampsia or Eclampsia | 6 | g i k n o t | 30 / 69 | 0.43 (0.32, 0.55) | 74 / 812 | 0.07 (0.03, 0.14) | **4.09 (2.08, 8.07)** | 48.19 (0.09) |  |
| Hypertensive Disorders of Pregnancy (Any) | 8 | d g i k l n o t | 53 / 109 | 0.49 (0.39, 0.59) | 198 / 2059 | 0.09 (0.06, 0.15) | **3.16 (2.24, 4.47)** | 23.24 (0.24) |  |
| Hypertensive Disorders of Pregnancy (At/After Covid-19) | 2 | l t | -- | -- | -- | -- | -- | -- |  |
| Preterm labor | 7 | d j k l n o t | 6 / 65 | 0.09 (0.04, 0.19) | 88 / 1911 | 0.04 (0.02, 0.07) | **3.93 (1.44, 10.75)** | 34.13 (0.17) |  |
| Preterm labor with onset before 37w GA **^2^** | 5 | d j l o t | 6 / 47 | 0.13 (0.06, 0.26) | 83 / 896 | 0.09 (0.08, 0.11) | 2.16 (0.73, 6.40) | 34.59 (0.19) |  |
| Cesarean Delivery | 11 | b d g h j k l n o s t | 78 / 141 | 0.54 (0.41, 0.66) | 1423 / 3915 | 0.36 (0.30, 0.43) | **1.31 (1.09, 1.57)** | 0 (0.98) |  |
| Intrapartum Cesarean Delivery | 8 | b d j k l n o t | 45 / 109 | 0.41 (0.32, 0.51) | 752 / 3568 | 0.21 (0.16, 0.28) | **1.58 (1.23, 2.04)** | 0 (0.98) |  |
|  |  |  |  |  |  |  |  |  |  |
| Stillbirth **^3^** | 15 | b d e f g* h* i j k l* n* o r s* t* | 4 / 187 | 9.68 (0.00, 41.64) **^5^** | 38 / 5124 | 7.34 (3.48, 12.30) **^5^** | **3.43 (1.41, 8.37)** | 0 (0.99) |  |
| Perinatal death | 11 | b d e j k l* n* o r s* t* | 4 / 132 | 12.49 (0.00, 55.93) | 39 / 4386 | 7.28 (4.33, 10.83) | **4.94 (2.07, 11.81)** | 0 (0.99) |  |
| Early neonatal death | 11 | b d e j* k* l* n* o* r* s* t* | 3 / 107 | 28.00 (9.05, 83.27) | 9 / 3803 | 2.36 (1.23, 4.54) | **11.74 (3.23, 42.70)** | 0 (0.96) |  |
| Neonatal death **^4^** | 12 | b d e i j* k l* n* o* r* s* t* | 3 / 133 | 14.91 (0.00, 54.26) | 15 / 4125 | 2.60 (1.01, 4.71) | **8.10 (2.71, 24.25)** | 0 (0.90) |  |
| NICU Admission at Birth | 6 | b d n r s t | 22 / 91 | 0.24 (0.16, 0.34) | 318 / 3097 | 0.17 (0.05, 0.46) | **2.28 (1.26, 4.13)** | 36.96 (0.16) |  |
|  |  |  |  |  |  |  |  |  |  |
| Very low birthweight (<1500g) | 12 | b d g i j* k l n o r* s t* | 14 / 147 | 0.03 (0.00, 0.09) | 76 / 3784 | 0.02 (0.01, 0.03) | **6.30 (3.16, 12.55)** | 17.41 (0.29) |  |
| Low birthweight (<2500g) | 12 | b d g i j k l n o r s t | 40 / 156 | 0.26 (0.19, 0.33) | 469 / 4027 | 0.12 (0.08, 0.17) | **1.87 (1.39, 2.50)** | 0 (0.89) |  |
| Small for gestational age (3rd) | 13 | b d e g i j* k l n o r s* t | 10 / 170 | 0.05 (0.02, 0.13) | 147 / 4832 | 0.03 (0.01, 0.06) | **3.34 (1.86, 6.00)** | 0 (0.60) |  |
| Small for gestational age (10th) | 13 | b d e g i j k l n o r s t | 22 / 178 | 0.12 (0.08, 0.18) | 412 / 5039 | 0.08 (0.05, 0.13) | **1.91 (1.29, 2.84)** | 0 (0.84) |  |
| Moderate preterm birth (<34w) | 13 | b d e g i j k l n o r s t | 30 / 178 | 0.17 (0.11, 0.24) | 233 / 5039 | 0.04 (0.03, 0.06) | **3.55 (2.48, 5.08)** | 0 (0.79) |  |
| Moderate preterm birth (<34w) with onset before 34w GA **^2^** | 7 | b d g j o s t | 19 / 74 | 0.25 (0.13, 0.42) | 146 / 1538 | 0.11 (0.07, 0.17) | **2.23 (1.46, 3.41)** | 0 (0.86) |  |
| Preterm birth (<37 wks) | 14 | b c2 d e g i j k l n o r s t | 73 / 200 | 0.34 (0.26, 0.43) | 795 / 5800 | 0.14 (0.11, 0.17) | **2.22 (1.72, 2.86)** | 18.89 (0.25) |  |
| Preterm birth (<37 wks) with onset before 37w GA **^2^** | 7 | b d g j o s t | 40 / 98 | 0.41 (0.31, 0.52) | 410 / 2036 | 0.23 (0.16, 0.32) | **1.61 (1.21, 2.12)** | 0 (0.77) |  |
| *Notes: Pooled absolute risks are calculated using a logistic-normal random-effects meta-analysis, pooling all participating studies with at least 1 adverse event for the given outcome; in cases where the model does not converge, we instead calculate pooled absolute risk using a random effects model with Freeman-Tukey Double Arcsine Transformation. For any study with zero events in one arm at the study level (Risk Group or Reference Group), we apply a continuity correction of 0.5 for pooled absolute risks. For any outcome with zero events for all studies in a given risk or reference group, we consider the absolute risk to be incalculable based on the current data. Relative risks are calculated by pooling unadjusted relative risks from all participating studies with at least 1 adverse event for the given outcome using a DerSimonian-Laird random effects model meta-analysis. For any study with zero events in one arm (Risk Group or Reference Group), we used a continuity correction of the inverse of the number of events in the oppposite group within the same study. We present pooled estimates for analyses where at least three studies report the relevant data and report at least 1 adverse event for the outcome of interest.  1 This column lists the studies that provide data for the given estimate. Any study sites indicated with an asterisk (*) reported "0" events in among those with and without the given risk factor. These "0 event" studies are excluded from the estimate and from the "Events/Total" column. Studies are identified as follows: a) Martinez-Portilla, 2021, Mexico; b) Favre, Panchaud, 2021, multicountry COVI-Preg registry; c1) Money, 2020 – Maternal Subset, Canada; c2) Money, 2020 – Infant Subset, Canada; d) Carrillo, 2021, Chile; e) Knight, 2021, United Kingdom; f) Bracero, Valencia, Delgado-Lopez, 2021, Puerto Rico (USA); g) Sakowicz, 2020, USA (Chicago); h) Sanin, Mesa, Tolosa, 2021, Colombia; i) Nachega, 2021, multicountry AFREHealth study; j) Waldorf, Lokken, 2021, USA (Washington State); k) Divakar, 2021, India (Karnataka State); l) Gil, Fernandez Buhigas, 2021, Spain (Madrid); m1) Crovetto, 2020, Cohort I, Spain (Barcelona); m2) Crovetto, 2020, Cohort II, Spain (Barcelona); n) Bevilacqua, Laurita Longo, 2020, Italy (Rome); o) Nunes, 2021, South Africa; p) Akelo, Tippett Barr, 2021, Kenya; q) Yang, Juan, 2020, China; r) Kalafat, 2020, Turkey; s) Brandt, 2020, USA (New Brunswick); t) Poon, 2021, Hong Kong (China). 2 These outcomes (preterm labor, moderate preterm birth before 34 weeks gestation, and preterm birth before 37 weeks’ gestation) were included in the sensitivity analyses where we restrict confirmed COVID-19 cases to those with confirmed COVID-19 onset prior to 37 weeks’ gestation (or 34 weeks for very moderate preterm birth). The full comparison group is used for each of the sensitivity analyses.  3 The outcome presented here is stillbirths occurring at or after 28 weeks gestational age per the WHO definition.  4 The outcome "neonatal death" is reported by 15 participating studies. However, most studies were not designed to follow-up neonates until 28 days after birth. Therefore, counts of neonatal death are underestimated.  5 The pooled absolute risk presented are calculated using a random effects model with Freeman-Tukey Double Arcsine Transformation, which was a better fit for the data in this case.* | | | | | | | | |  |
|  |  |  |  |  |  |  |  |  |  |
|  |  |  |  |  |  |  |  |  |  |
|  |  |  |  |  |  |  |  |  |  |
|  |  |  |  |  |  |  |  |  |  |
|  |  |  |  |  |  |  |  |  |  |
|  |  |  |  |  |  |  |  |  |  |
|  |  |  |  |  |  |  |  |  |  |
|  |  |  |  |  |  |  |  |  |  |
|  |  |  |  |  |  |  |  |  |  |
|  |  |  |  |  |  |  |  |  |  |
|  |  |  |  |  |  |  |  |  |  |
|  |  |  |  |  |  |  |  |  |  |
|  |  |  |  |  |  |  |  |  |  |
|  |  |  |  |  |  |  |  |  |  |
|  |  |  |  |  |  |  |  |  |  |

Table S9. Risk of Outcomes among COVID+ pregnancy, stratified by chronic cardiovascular disease

| Outcome | N Studies | Included Studies **^1^** | With Cardiovascular Disease | | Without Cardiovascular Disease | | Pooled, unadjusted RR (95% CI) | I^2 (pvalue) |  |
| --- | --- | --- | --- | --- | --- | --- | --- | --- | --- |
|  |  |  | Events/Total | Pooled Risk (95% CI) | Events/Total | Pooled Risk (95% CI) |  |  |  |
|  |  |  |  |  |  |  |  |  |  |
| ICU admission | 12 | a b c1 d e g i j l* m1* m2 n | 15 / 228 | 0.08 (0.03, 0.19) | 562 / 18744 | 0.03 (0.02, 0.05) | **2.98 (1.83, 4.85)** | 4.24 (0.40) |  |
| Ventilation | 12 | a b c1 d e i j k l m1* m2 n | 5 / 232 | 0.02 (0.01, 0.05) | 272 / 18982 | 0.01 (0.01, 0.02) | **6.11 (2.85, 13.08)** | 0 (0.96) |  |
| Critical Care | 11 | a b c1 d e i j l m1* m2 n | 15 / 230 | 0.08 (0.03, 0.18) | 615 / 18772 | 0.03 (0.02, 0.05) | **2.82 (1.78, 4.48)** | 0 (0.61) |  |
| Pneumonia | 8 | a b d e l m1* m2 n | 9 / 121 | 0.07 (0.03, 0.16) | 1274 / 16430 | 0.06 (0.03, 0.11) | 1.18 (0.65, 2.16) | 0 (0.83) |  |
| Pregnancy-related death | 11 | a b d e j k* l* m1* m2* n* o | 2 / 104 | 1311.96 (79.88, 18104.04) | 162 / 15264 | 760.89 (463.07, 1247.83) | **16.76 (4.42, 63.64)** | 0 (0.49) |  |
|  |  |  |  |  |  |  |  |  |  |
| Haemorrhage | 5 | g j l n o | 2 / 19 | 0.11 (0.03, 0.34) | 78 / 789 | 0.07 (0.02, 0.20) | 2.42 (0.29, 20.01) | 22.51 (0.27) |  |
| Placental Abruption | 3 | g j l | 0 / 14 | *incalculable* | 5 / 498 | 0.01 (0.00, 0.02) | **9.99 (1.70, 58.58)** | 0 (0.94) |  |
| Preeclampsia | 8 | b d g k l m1 m2 o | 6 / 55 | 0.09 (0.02, 0.33) | 175 / 3899 | 0.05 (0.03, 0.08) | **4.78 (2.24, 10.22)** | 0 (0.82) |  |
| Preeclampsia or Eclampsia | 6 | g k m1 m2 n o | 5 / 23 | 0.22 (0.09, 0.43) | 76 / 1000 | 0.06 (0.03, 0.11) | **6.38 (2.80, 14.58)** | 0 (0.74) |  |
| Hypertensive Disorders of Pregnancy (Any) | 8 | d g k l m1 m2 n o | 7 / 34 | 0.19 (0.06, 0.43) | 212 / 2276 | 0.08 (0.05, 0.13) | **4.29 (2.17, 8.48)** | 0 (0.68) |  |
| Hypertensive Disorders of Pregnancy (At/After Covid-19) | 1 | l | -- | -- | -- | -- | -- | -- |  |
| Preterm labor | 8 | d j k l m1* m2 n o | 2 / 30 | 0.15 (0.02, 0.33) **^5^** | 104 / 2097 | 0.04 (0.03, 0.07) **^5^** | **3.94 (1.39, 11.19)** | 0 (0.63) |  |
| Preterm labor with onset before 37w GA **^2^** | 5 | d j l n o | 0 / 17 | *incalculable* | 87 / 953 | 0.09 (0.07, 0.11) | 2.40 (0.31, 18.46) | 0 (0.69) |  |
| Cesarean Delivery | 10 | b d g j k l m1 m2 n o | 29 / 60 | 0.62 (0.26, 0.88) | 1489 / 4106 | 0.35 (0.28, 0.42) | **1.44 (1.08, 1.92)** | 0 (0.96) |  |
| Intrapartum Cesarean Delivery | 9 | b d j k l m1* m2 n o | 15 / 51 | 0.29 (0.19, 0.43) | 807 / 3778 | 0.20 (0.15, 0.27) | **1.59 (1.03, 2.48)** | 0 (0.98) |  |
|  |  |  |  |  |  |  |  |  |  |
| Stillbirth **^3^** | 12 | b d e f* g* j k l* m1 m2* n* o | 1 / 62 | 20.21 (0.00, 101.71) **5** | 30 / 4538 | 6.01 (2.83, 10.11) **^5^** | **9.10 (2.24, 36.92)** | 0 (0.88) |  |
| Perinatal death | 10 | b d e j k l* m1 m2 n* o | 2 / 69 | 28.99 (7.26, 108.60) | 42 / 4709 | 8.92 (6.60, 12.05) | **8.47 (2.70, 26.53)** | 0 (0.95) |  |
| Early neonatal death | 10 | b d e j* k* l* m1* m2 n* o* | 1 / 55 | 18.23 (2.57, 118.07) | 12 / 4033 | 2.98 (1.69, 5.23) | **12.58 (2.69, 58.80)** | 0 (0.95) |  |
| Neonatal death **^4^** | 10 | b d e j* k l* m1* m2 n* o* | 1 / 57 | 18.02 (0.00, 99.14) | 17 / 4243 | 3.25 (1.53, 5.45) | **13.04 (3.18, 53.43)** | 0 (0.89) |  |
| NICU Admission at Birth | 5 | b d m1 m2 n | 4 / 43 | 0.09 (0.04, 0.22) | 278 / 3322 | 0.05 (0.03, 0.09) | 2.02 (0.65, 6.30) | 20.1 (0.29) |  |
|  |  |  |  |  |  |  |  |  |  |
| Very low birthweight (<1500g) | 10 | b d g j* k l m1 m2 n o | 5 / 57 | 0.09 (0.04, 0.19) | 80 / 4012 | 0.02 (0.02, 0.02) | **8.35 (3.64, 19.19)** | 0 (0.80) |  |
| Low birthweight (<2500g) | 10 | b d g j k l m1 m2 n o | 11 / 61 | 0.18 (0.10, 0.30) | 471 / 4164 | 0.10 (0.07, 0.15) | **2.01 (1.19, 3.39)** | 0 (0.99) |  |
| Small for gestational age (3rd) | 11 | b d e g j* k l m1 m2 n o | 5 / 75 | 0.09 (0.01, 0.24) **^5^** | 152 / 5028 | 0.04 (0.02, 0.06) **^5^** | **3.14 (1.58, 6.23)** | 0 (0.86) |  |
| Small for gestational age (10th) | 11 | b d e g j k l m1 m2 n o | 12 / 79 | 0.15 (0.09, 0.25) | 429 / 5180 | 0.09 (0.06, 0.14) | **1.84 (1.11, 3.03)** | 0 (1.00) |  |
| Moderate preterm birth (<34w) | 11 | b d e g j k l m1 m2 n o | 7 / 79 | 0.09 (0.04, 0.17) | 241 / 5180 | 0.04 (0.02, 0.05) | **3.04 (1.57, 5.91)** | 0 (0.87) |  |
| Moderate preterm birth (<34w) with onset before 34w GA **^2^** | 5 | b g j n o | 3 / 17 | 0.18 (0.06, 0.43) | 101 / 1202 | 0.10 (0.06, 0.16) | 2.27 (0.93, 5.50) | 0 (1.00) |  |
| Preterm birth (<37 wks) | 12 | b c2 d e g j k l m1 m2 n o | 41 / 170 | 0.24 (0.18, 0.31) | 784 / 5872 | 0.12 (0.09, 0.15) | **1.90 (1.41, 2.56)** | 0 (0.94) |  |
| Preterm birth (<37 wks) with onset before 37w GA **^2^** | 6 | b d g j n o | 6 / 27 | 0.22 (0.10, 0.41) | 444 / 2123 | 0.23 (0.17, 0.30) | 1.25 (0.63, 2.49) | 0 (0.98) |  |
| *Notes: Pooled absolute risks are calculated using a logistic-normal random-effects meta-analysis, pooling all participating studies with at least 1 adverse event for the given outcome; in cases where the model does not converge, we instead calculate pooled absolute risk using a random effects model with Freeman-Tukey Double Arcsine Transformation. For any study with zero events in one arm at the study level (Risk Group or Reference Group), we apply a continuity correction of 0.5 for pooled absolute risks. For any outcome with zero events for all studies in a given risk or reference group, we consider the absolute risk to be incalculable based on the current data. Relative risks are calculated by pooling unadjusted relative risks from all participating studies with at least 1 adverse event for the given outcome using a DerSimonian-Laird random effects model meta-analysis. For any study with zero events in one arm (Risk Group or Reference Group), we used a continuity correction of the inverse of the number of events in the oppposite group within the same study. We present pooled estimates for analyses where at least three studies report the relevant data and report at least 1 adverse event for the outcome of interest.  1 This column lists the studies that provide data for the given estimate. Any study sites indicated with an asterisk (*) reported "0" events in among those with and without the given risk factor. These "0 event" studies are excluded from the estimate and from the "Events/Total" column. Studies are identified as follows: a) Martinez-Portilla, 2021, Mexico; b) Favre, Panchaud, 2021, multicountry COVI-Preg registry; c1) Money, 2020 – Maternal Subset, Canada; c2) Money, 2020 – Infant Subset, Canada; d) Carrillo, 2021, Chile; e) Knight, 2021, United Kingdom; f) Bracero, Valencia, Delgado-Lopez, 2021, Puerto Rico (USA); g) Sakowicz, 2020, USA (Chicago); h) Sanin, Mesa, Tolosa, 2021, Colombia; i) Nachega, 2021, multicountry AFREHealth study; j) Waldorf, Lokken, 2021, USA (Washington State); k) Divakar, 2021, India (Karnataka State); l) Gil, Fernandez Buhigas, 2021, Spain (Madrid); m1) Crovetto, 2020, Cohort I, Spain (Barcelona); m2) Crovetto, 2020, Cohort II, Spain (Barcelona); n) Bevilacqua, Laurita Longo, 2020, Italy (Rome); o) Nunes, 2021, South Africa; p) Akelo, Tippett Barr, 2021, Kenya; q) Yang, Juan, 2020, China; r) Kalafat, 2020, Turkey; s) Brandt, 2020, USA (New Brunswick); t) Poon, 2021, Hong Kong (China). 2 These outcomes (preterm labor, moderate preterm birth before 34 weeks gestation, and preterm birth before 37 weeks’ gestation) were included in the sensitivity analyses where we restrict confirmed COVID-19 cases to those with confirmed COVID-19 onset prior to 37 weeks’ gestation (or 34 weeks for very moderate preterm birth). The full comparison group is used for each of the sensitivity analyses.  3 The outcome presented here is stillbirths occurring at or after 28 weeks gestational age per the WHO definition.  4 The outcome "neonatal death" is reported by 15 participating studies. However, most studies were not designed to follow-up neonates until 28 days after birth. Therefore, counts of neonatal death are underestimated.  5 The pooled absolute risk presented are calculated using a random effects model with Freeman-Tukey Double Arcsine Transformation, which was a better fit for the data in this case.* | | | | | | | | |  |
|  |  |  |  |  |  |  |  |  |  |
|  |  |  |  |  |  |  |  |  |  |
|  |  |  |  |  |  |  |  |  |  |
|  |  |  |  |  |  |  |  |  |  |
|  |  |  |  |  |  |  |  |  |  |
|  |  |  |  |  |  |  |  |  |  |
|  |  |  |  |  |  |  |  |  |  |
|  |  |  |  |  |  |  |  |  |  |
|  |  |  |  |  |  |  |  |  |  |
|  |  |  |  |  |  |  |  |  |  |
|  |  |  |  |  |  |  |  |  |  |
|  |  |  |  |  |  |  |  |  |  |
|  |  |  |  |  |  |  |  |  |  |
|  |  |  |  |  |  |  |  |  |  |
|  |  |  |  |  |  |  |  |  |  |

Table S10. Risk of Outcomes among COVID+ pregnancy, stratified by HIV

| Outcome | N Studies | Included Studies **^1^** | With HIV | | Without HIV | | Pooled, unadjusted RR (95% CI) | I^2 (pvalue) |  |
| --- | --- | --- | --- | --- | --- | --- | --- | --- | --- |
|  |  |  | Events/Total | Pooled Risk (95% CI) | Events/Total | Pooled Risk (95% CI) |  |  |  |
|  |  |  |  |  |  |  |  |  |  |
| ICU admission | 3 | b i j | 22 / 80 | 0.28 (0.19, 0.38) | 108 / 2070 | 0.06 (0.03, 0.14) | **1.67 (1.06, 2.63)** | 0 (0.91) |  |
| Ventilation | 3 | b i j | 3 / 80 | 0.04 (0.01, 0.11) | 46 / 2070 | 0.03 (0.02, 0.04) | 1.01 (0.30, 3.32) | 0 (0.84) |  |
| Critical Care | 3 | b i j | 23 / 80 | 0.29 (0.20, 0.40) | 110 / 2070 | 0.06 (0.03, 0.14) | **1.72 (1.10, 2.69)** | 0 (0.92) |  |
| Pneumonia | 1 | b | -- | -- | -- | -- | -- | -- |  |
| Pregnancy-related death | 4 | b i j o | 14 / 96 | 1994.78 (19.29, 68223.01) | 28 / 1835 | 1894.07 (355.1, 9468.97) | 2.70 (0.58, 12.47) | 42.77 (0.15) |  |
|  |  |  |  |  |  |  |  |  |  |
| Haemorrhage | 3 | i j o | 13 / 91 | 0.14 (0.08, 0.23) | 52 / 361 | 0.14 (0.11, 0.19) | 1.06 (0.57, 1.99) | 0 (0.95) |  |
| Placental Abruption | 2 | i j | -- | -- | -- | -- | -- | -- |  |
| Preeclampsia | 2 | b o | -- | -- | -- | -- | -- | -- |  |
| Preeclampsia or Eclampsia | 2 | i o | -- | -- | -- | -- | -- | -- |  |
| Hypertensive Disorders of Pregnancy (Any) | 2 | i o | -- | -- | -- | -- | -- | -- |  |
| Hypertensive Disorders of Pregnancy (At/After Covid-19) | 0 | -- | -- | -- | -- | -- | -- | -- |  |
| Preterm labor | 2 | j o | -- | -- | -- | -- | -- | -- |  |
| Preterm labor with onset before 37w GA **^2^** | 2 | j o | -- | -- | -- | -- | -- | -- |  |
| Cesarean Delivery | 3 | b j o | 26 / 35 | 0.89 (0.57, 1) | 528 / 1653 | 0.38 (0.27, 0.5) | **1.51 (1.00, 2.28)** | 21.28 (0.28) |  |
| Intrapartum Cesarean Delivery | 3 | b j o | 16 / 35 | 0.46 (0.3, 0.62) | 304 / 1653 | 0.22 (0.14, 0.31) | 1.47 (0.91, 2.37) | 2.64 (0.36) |  |
|  |  |  |  |  |  |  |  |  |  |
| Stillbirth **^3^** | 4 | b i j o | 2 / 86 | 23.26 (5.83, 88.24) | 16 / 1787 | 12.96 (4.35, 37.95) | 2.97 (0.35, 25.26) | 54.25 (0.09) |  |
| Perinatal death | 3 | b j o | 1 / 37 | 27.03 (3.79, 168.47) | 13 / 1690 | 7.69 (4.47, 13.2) | **8.63 (1.40, 53.31)** | 0 (0.54) |  |
| Early neonatal death | 3 | b j* o* | -- | -- | -- | -- | -- | -- |  |
| Neonatal death **^4^** | 4 | b i j* o* | -- | -- | -- | -- | -- | -- |  |
| NICU Admission at Birth | 1 | b | -- | -- | -- | -- | -- | -- |  |
|  |  |  |  |  |  |  |  |  |  |
| Very low birthweight (<1500g) | 4 | b i j* o | 6 / 82 | 0.07 (0.03, 0.15) | 37 / 1619 | 0.02 (0.02, 0.03) | 2.41 (0.80, 7.20) | 0 (0.82) |  |
| Low birthweight (<2500g) | 4 | b i j o | 29 / 83 | 0.35 (0.22, 0.5) | 220 / 1769 | 0.14 (0.07, 0.26) | 1.38 (0.93, 2.04) | 0 (0.62) |  |
| Small for gestational age (3rd) | 4 | b i j* o | 10 / 82 | 0.12 (0.04, 0.3) | 50 / 1619 | 0.04 (0.02, 0.1) | **2.14 (1.02, 4.48)** | 4.10 (0.35) |  |
| Small for gestational age (10th) | 4 | b i j o | 18 / 83 | 0.22 (0.13, 0.35) | 149 / 1769 | 0.1 (0.06, 0.15) | 1.57 (0.93, 2.63) | 0 (0.97) |  |
| Moderate preterm birth (<34w) | 4 | b i j o | 12 / 83 | 0.13 (0.05, 0.29) | 89 / 1769 | 0.06 (0.04, 0.09) | 1.78 (0.67, 4.74) | 27.81 (0.25) |  |
| Moderate preterm birth (<34w) with onset before 34w GA **^2^** | 3 | b j o | 8 / 23 | 0.35 (0.18, 0.56) | 75 / 952 | 0.08 (0.06, 0.1) | 2.18 (0.93, 5.07) | 0 (0.91) |  |
| Preterm birth (<37 wks) | 4 | b i j o | 28 / 83 | 0.34 (0.24, 0.45) | 226 / 1769 | 0.17 (0.1, 0.27) | 1.22 (0.83, 1.81) | 0 (0.80) |  |
| Preterm birth (<37 wks) with onset before 37w GA **2** | 3 | b j o | 13 / 29 | 0.45 (0.28, 0.63) | 186 / 1197 | 0.19 (0.12, 0.28) | 1.40 (0.81, 2.41) | 0 (0.94) |  |
| *Notes: Pooled absolute risks are calculated using a logistic-normal random-effects meta-analysis, pooling all participating studies with at least 1 adverse event for the given outcome; in cases where the model does not converge, we instead calculate pooled absolute risk using a random effects model with Freeman-Tukey Double Arcsine Transformation. For any study with zero events in one arm at the study level (Risk Group or Reference Group), we apply a continuity correction of 0.5 for pooled absolute risks. For any outcome with zero events for all studies in a given risk or reference group, we consider the absolute risk to be incalculable based on the current data. Relative risks are calculated by pooling unadjusted relative risks from all participating studies with at least 1 adverse event for the given outcome using a DerSimonian-Laird random effects model meta-analysis. For any study with zero events in one arm (Risk Group or Reference Group), we used a continuity correction of the inverse of the number of events in the oppposite group within the same study. We present pooled estimates for analyses where at least three studies report the relevant data and report at least 1 adverse event for the outcome of interest.  1 This column lists the studies that provide data for the given estimate. Any study sites indicated with an asterisk (*) reported "0" events in among those with and without the given risk factor. These "0 event" studies are excluded from the estimate and from the "Events/Total" column. Studies are identified as follows: a) Martinez-Portilla, 2021, Mexico; b) Favre, Panchaud, 2021, multicountry COVI-Preg registry; c1) Money, 2020 – Maternal Subset, Canada; c2) Money, 2020 – Infant Subset, Canada; d) Carrillo, 2021, Chile; e) Knight, 2021, United Kingdom; f) Bracero, Valencia, Delgado-Lopez, 2021, Puerto Rico (USA); g) Sakowicz, 2020, USA (Chicago); h) Sanin, Mesa, Tolosa, 2021, Colombia; i) Nachega, 2021, multicountry AFREHealth study; j) Waldorf, Lokken, 2021, USA (Washington State); k) Divakar, 2021, India (Karnataka State); l) Gil, Fernandez Buhigas, 2021, Spain (Madrid); m1) Crovetto, 2020, Cohort I, Spain (Barcelona); m2) Crovetto, 2020, Cohort II, Spain (Barcelona); n) Bevilacqua, Laurita Longo, 2020, Italy (Rome); o) Nunes, 2021, South Africa; p) Akelo, Tippett Barr, 2021, Kenya; q) Yang, Juan, 2020, China; r) Kalafat, 2020, Turkey; s) Brandt, 2020, USA (New Brunswick); t) Poon, 2021, Hong Kong (China). 2 These outcomes (preterm labor, moderate preterm birth before 34 weeks gestation, and preterm birth before 37 weeks’ gestation) were included in the sensitivity analyses where we restrict confirmed COVID-19 cases to those with confirmed COVID-19 onset prior to 37 weeks’ gestation (or 34 weeks for very moderate preterm birth). The full comparison group is used for each of the sensitivity analyses.  3 The outcome presented here is stillbirths occurring at or after 28 weeks gestational age per the WHO definition.  4 The outcome "neonatal death" is reported by 15 participating studies. However, most studies were not designed to follow-up neonates until 28 days after birth. Therefore, counts of neonatal death are underestimated.* | | | | | | | | |  |
|  |  |  |  |  |  |  |  |  |  |
|  |  |  |  |  |  |  |  |  |  |
|  |  |  |  |  |  |  |  |  |  |
|  |  |  |  |  |  |  |  |  |  |
|  |  |  |  |  |  |  |  |  |  |
|  |  |  |  |  |  |  |  |  |  |
|  |  |  |  |  |  |  |  |  |  |
|  |  |  |  |  |  |  |  |  |  |
|  |  |  |  |  |  |  |  |  |  |
|  |  |  |  |  |  |  |  |  |  |
|  |  |  |  |  |  |  |  |  |  |
|  |  |  |  |  |  |  |  |  |  |
|  |  |  |  |  |  |  |  |  |  |
|  |  |  |  |  |  |  |  |  |  |
|  |  |  |  |  |  |  |  |  |  |

Table S11. Risk of Outcomes among COVID+ pregnancy, stratified by BMI (>30) – ref group = BMI 18.5-29

| Outcome | N Studies | Included Studies **^1^** | With BMI ≥ 30 | | With BMI 18.5-29.9 | | |  | |  |
| --- | --- | --- | --- | --- | --- | --- | --- | --- | --- | --- |
|  |  |  | Events/Total | Pooled Risk (95% CI) | | Events/Total | Pooled Risk (95% CI) | Pooled, unadjusted RR (95% CI) | I^2 (pvalue) |  |
|  |  |  |  |  |  | |  |  |  |  |
| ICU admission | 8 | c1 d h j l* m1* m2 n | 55 / 763 | 0.05 (0.02, 0.11) | 62 / 1675 | | 0.03 (0.01, 0.07) | **1.81 (1.26, 2.60)** | 0 (0.86) |  |
| Ventilation | 7 | c1 d j l m1* m2 n | 27 / 765 | 0.02 (0.01, 0.06) | 27 / 1774 | | 0.01 (0.01, 0.02) | **2.05 (1.20, 3.51)** | 0 (0.96) |  |
| Critical Care | 7 | c1 d j l m1* m2 n | 50 / 765 | 0.03 (0.01, 0.09) | 52 / 1774 | | 0.02 (0.01, 0.04) | **1.89 (1.28, 2.77)** | 0 (0.93) |  |
| Pneumonia | 5 | d l m1* m2 n | 58 / 461 | 0.13 (0.10, 0.16) | 74 / 1108 | | 0.06 (0.04, 0.09) | **1.66 (1.18, 2.33)** | 0 (0.83) |  |
| Pregnancy-related death | 7 | d h j l* m1* m2* n* | 2 / 399 | 258.08 (4.20, 13739.05) | 6 / 687 | | 1223.94 (264.99, 5463.08) | 1.00 (0.19, 5.26) | 0 (0.51) |  |
|  |  |  |  |  |  | |  |  |  |  |
| Haemorrhage | 4 | h j l n | 20 / 129 | 0.06 (0.01, 0.41) | 33 / 383 | | 0.06 (0.02, 0.18) | 1.43 (0.85, 2.41) | 0 (0.94) |  |
| Placental Abruption | 2 | j l | -- | -- | -- | | -- | -- | -- |  |
| Preeclampsia | 4 | d l m1 m2 | 30 / 384 | 0.08 (0.06, 0.11) | 48 / 977 | | 0.05 (0.04, 0.06) | **1.60 (1.01, 2.54)** | 0 (0.56) |  |
| Preeclampsia or Eclampsia | 3 | m1 m2 n | 3 / 70 | 0.05 (0.00, 0.15) **^5^** | 17 / 399 | | 0.04 (0.02, 0.07) **^5^** | 2.16 (0.68, 6.82) | 0 (0.46) |  |
| Hypertensive Disorders of Pregnancy (Any) | 5 | d l m1 m2 n | 52 / 412 | 0.13 (0.10, 0.16) | 67 / 1092 | | 0.06 (0.05, 0.08) | **1.86 (1.30, 2.67)** | 0 (0.99) |  |
| Hypertensive Disorders of Pregnancy (At/After Covid-19) | 1 | l | -- | -- | -- | | -- | -- | -- |  |
| Preterm labor | 6 | d j l m1* m2 n | 24 / 455 | 0.05 (0.04, 0.08) | 61 / 1042 | | 0.06 (0.04, 0.08) | 0.91 (0.57, 1.46) | 0 (0.70) |  |
| Preterm labor with onset before 37w GA **^2^** | 4 | d j l n | 22 / 249 | 0.09 (0.06, 0.13) | 47 / 482 | | 0.09 (0.06, 0.14) | 0.84 (0.51, 1.39) | 0.24 (0.39) |  |
| Cesarean Delivery | 7 | d h j l m1 m2 n | 244 / 476 | 0.40 (0.28, 0.53) | 410 / 1193 | | 0.30 (0.24, 0.36) | **1.23 (1.07, 1.41)** | 0 (0.93) |  |
| Intrapartum Cesarean Delivery | 6 | d j l m1* m2 n | 140 / 445 | 0.21 (0.13, 0.33) | 211 / 1023 | | 0.17 (0.12, 0.23) | **1.28 (1.06, 1.56)** | 0 (0.90) |  |
|  |  |  |  |  |  | |  |  |  |  |
| Stillbirth **^3^** | 8 | d f h* j l* m1 m2* n* | 4 / 568 | 7.80 (1.44, 41.11) | 6 / 1097 | | 5.47 (2.46, 12.12) | 1.89 (0.31, 11.60) | 26.43 (0.25) |  |
| Perinatal death | 6 | d j l* m1 m2 n* | 3 / 418 | 12.35 (0.00, 59.49) **^5^** | 8 / 920 | | 8.40 (2.76, 16.27) **^5^** | 3.17 (0.43, 23.21) | 18.84 (0.30) |  |
| Early neonatal death | 6 | d j* l* m1* m2 n* | -- | -- | -- | | -- | -- | -- |  |
| Neonatal death **^4^** | 6 | d j* l* m1* m2 n* | -- | -- | -- | | -- | -- | -- |  |
| NICU Admission at Birth | 4 | d m1 m2 n | 20 / 384 | 0.05 (0.03, 0.08) | 35 / 943 | | 0.04 (0.03, 0.05) | 1.42 (0.82, 2.47) | 0 (0.98) |  |
|  |  |  |  |  |  | |  |  |  |  |
| Very low birthweight (<1500g) | 6 | d j* l m1 m2 n | 9 / 408 | 0.02 (0.01, 0.04) | 14 / 1081 | | 0.01 (0.01, 0.02) | 1.70 (0.76, 3.79) | 0 (0.86) |  |
| Low birthweight (<2500g) | 6 | d j l m1 m2 n | 43 / 467 | 0.08 (0.04, 0.16) | 104 / 1164 | | 0.08 (0.06, 0.11) | 0.97 (0.68, 1.37) | 0 (0.99) |  |
| Small for gestational age (3rd) | 6 | d j* l m1 m2 n* | 4 / 380 | 0.01 (0.00, 0.03) | 23 / 971 | | 0.02 (0.02, 0.04) | 0.68 (0.24, 1.95) | 1.06 (0.39) |  |
| Small for gestational age (10th) | 6 | d j l m1 m2 n | 16 / 467 | 0.04 (0.02, 0.08) | 83 / 1164 | | 0.08 (0.06, 0.10) | 0.75 (0.41, 1.37) | 12.28 (0.34) |  |
| Moderate preterm birth (<34w) | 6 | d j l m1 m2 n | 28 / 467 | 0.05 (0.03, 0.08) **^5^** | 36 / 1164 | | 0.03 (0.02, 0.04) **^5^** | **1.75 (1.06, 2.89)** | 0 (0.93) |  |
| Moderate preterm birth (<34w) with onset before 34w GA **^2^** | 3 | d j n | 28 / 163 | 0.17 (0.12, 0.24) | 27 / 233 | | 0.12 (0.08, 0.16) | 1.46 (0.89, 2.40) | 0 (0.91) |  |
| Preterm birth (<37 wks) | 7 | c2 d j l m1 m2 n | 113 / 617 | 0.11 (0.05, 0.20) | 178 / 1559 | | 0.09 (0.06, 0.12) | **1.38 (1.10, 1.73)** | 0 (0.81) |  |
| Preterm birth (<37 wks) with onset before 37w GA **^2^** | 3 | d j n | 78 / 231 | 0.28 (0.16, 0.44) | 98 / 358 | | 0.20 (0.11, 0.34) | 1.17 (0.90, 1.51) | 0 (0.86) |  |
| *Notes: Pooled absolute risks are calculated using a logistic-normal random-effects meta-analysis, pooling all participating studies with at least 1 adverse event for the given outcome; in cases where the model does not converge, we instead calculate pooled absolute risk using a random effects model with Freeman-Tukey Double Arcsine Transformation. For any study with zero events in one arm at the study level (Risk Group or Reference Group), we apply a continuity correction of 0.5 for pooled absolute risks. For any outcome with zero events for all studies in a given risk or reference group, we consider the absolute risk to be incalculable based on the current data. Relative risks are calculated by pooling unadjusted relative risks from all participating studies with at least 1 adverse event for the given outcome using a DerSimonian-Laird random effects model meta-analysis. For any study with zero events in one arm (Risk Group or Reference Group), we used a continuity correction of the inverse of the number of events in the oppposite group within the same study. We present pooled estimates for analyses where at least three studies report the relevant data and report at least 1 adverse event for the outcome of interest.  1 This column lists the studies that provide data for the given estimate. Any study sites indicated with an asterisk (*) reported "0" events in among those with and without the given risk factor. These "0 event" studies are excluded from the estimate and from the "Events/Total" column. Studies are identified as follows: a) Martinez-Portilla, 2021, Mexico; b) Favre, Panchaud, 2021, multicountry COVI-Preg registry; c1) Money, 2020 – Maternal Subset, Canada; c2) Money, 2020 – Infant Subset, Canada; d) Carrillo, 2021, Chile; e) Knight, 2021, United Kingdom; f) Bracero, Valencia, Delgado-Lopez, 2021, Puerto Rico (USA); g) Sakowicz, 2020, USA (Chicago); h) Sanin, Mesa, Tolosa, 2021, Colombia; i) Nachega, 2021, multicountry AFREHealth study; j) Waldorf, Lokken, 2021, USA (Washington State); k) Divakar, 2021, India (Karnataka State); l) Gil, Fernandez Buhigas, 2021, Spain (Madrid); m1) Crovetto, 2020, Cohort I, Spain (Barcelona); m2) Crovetto, 2020, Cohort II, Spain (Barcelona); n) Bevilacqua, Laurita Longo, 2020, Italy (Rome); o) Nunes, 2021, South Africa; p) Akelo, Tippett Barr, 2021, Kenya; q) Yang, Juan, 2020, China; r) Kalafat, 2020, Turkey; s) Brandt, 2020, USA (New Brunswick); t) Poon, 2021, Hong Kong (China). 2 These outcomes (preterm labor, moderate preterm birth before 34 weeks gestation, and preterm birth before 37 weeks’ gestation) were included in the sensitivity analyses where we restrict confirmed COVID-19 cases to those with confirmed COVID-19 onset prior to 37 weeks’ gestation (or 34 weeks for very moderate preterm birth). The full comparison group is used for each of the sensitivity analyses.  3 The outcome presented here is stillbirths occurring at or after 28 weeks gestational age per the WHO definition.  4 The outcome "neonatal death" is reported by 15 participating studies. However, most studies were not designed to follow-up neonates until 28 days after birth. Therefore, counts of neonatal death are underestimated.  5 The pooled absolute risk presented are calculated using a random effects model with Freeman-Tukey Double Arcsine Transformation, which was a better fit for the data in this case.* | | | | | | | | | |  |
|  |  |  |  |  |  |  |  |  |  |  |
|  |  |  |  |  |  |  |  |  |  |  |
|  |  |  |  |  |  |  |  |  |  |  |
|  |  |  |  |  |  |  |  |  |  |  |
|  |  |  |  |  |  |  |  |  |  |  |
|  |  |  |  |  |  |  |  |  |  |  |
|  |  |  |  |  |  |  |  |  |  |  |
|  |  |  |  |  |  |  |  |  |  |  |
|  |  |  |  |  |  |  |  |  |  |  |
|  |  |  |  |  |  |  |  |  |  |  |
|  |  |  |  |  |  |  |  |  |  |  |
|  |  |  |  |  |  |  |  |  |  |  |
|  |  |  |  |  |  |  |  |  |  |  |
|  |  |  |  |  |  |  |  |  |  |  |
|  |  |  |  |  |  |  |  |  |  |  |

Table S12. Risk of Outcomes among COVID+ pregnancy, stratified by BMI (<18.5, 18.5-29) – ref group = BMI 18.5-29

| Outcome | N Studies | Included Studies **^1^** | With BMI < 18.5 | | With BMI 18.5-29.9 | |  | |  |
| --- | --- | --- | --- | --- | --- | --- | --- | --- | --- |
|  |  |  | Events/Total | Pooled Risk (95% CI) | Events/Total | Pooled Risk (95% CI) | Pooled, unadjusted RR (95% CI) | I^2 (pvalue) |  |
|  |  |  |  |  |  |  |  |  |  |
| ICU admission | 8 | c1 d h j l* m1* m2 n | 3 / 46 | 0.08 (0.00, 0.25) **^5^** | 62 / 1675 | 0.03 (0.01, 0.06) **^5^** | **5.53 (2.27, 13.44)** | 0 (0.75) |  |
| Ventilation | 7 | c1 d j l m1* m2 n | 3 / 48 | 0.08 (0.00, 0.22) **^5^** | 27 / 1774 | 0.01 (0.01, 0.02) **^5^** | **9.36 (3.87, 22.63)** | 0 (0.87) |  |
| Critical Care | 7 | c1 d j l m1* m2 n | 3 / 48 | 0.08 (0.00, 0.22) **^5^** | 52 / 1774 | 0.02 (0.01, 0.04) **^5^** | **5.71 (2.40, 13.59)** | 0 (0.82) |  |
| Pneumonia | 5 | d l m1* m2 n | 4 / 21 | 0.19 (0.07, 0.41) | 74 / 1108 | 0.06 (0.04, 0.09) | **2.71 (1.13, 6.49)** | 0 (0.94) |  |
| Pregnancy-related death | 7 | d h j l* m1* m2* n* | 1 / 13 | 7692.31 (1071.96, 39057.13) | 6 / 687 | 1223.94 (264.99, 5463.08) | **14.10 (2.83, 70.36)** | 0 (0.64) |  |
|  |  |  |  |  |  |  |  |  |  |
| Haemorrhage | 4 | h j l n | 0 / 11 | *incalculable* | 33 / 383 | 0.06 (0.02, 0.18) | 6.00 (0.89, 40.41) | 0 (0.56) |  |
| Placental Abruption | 2 | j l | -- | -- | -- | -- | -- | -- |  |
| Preeclampsia | 4 | d l m1 m2 | 2 / 23 | 0.09 (0.02, 0.29) | 48 / 977 | 0.05 (0.04, 0.06) | 2.18 (0.63, 7.53) | 0 (0.98) |  |
| Preeclampsia or Eclampsia | 3 | m1 m2 n | 1 / 13 | 0.08 (0.01, 0.39) | 17 / 399 | 0.04 (0.03, 0.07) | 3.08 (0.64, 14.81) | 0 (0.81) |  |
| Hypertensive Disorders of Pregnancy (Any) | 5 | d l m1 m2 n | 2 / 27 | 0.07 (0.02, 0.25) | 67 / 1092 | 0.06 (0.05, 0.08) | 1.93 (0.59, 6.26) | 0 (0.90) |  |
| Hypertensive Disorders of Pregnancy (At/After Covid-19) | 1 | l | -- | -- | -- | -- | -- | -- |  |
| Preterm labor | 6 | d j l m1* m2 n | 1 / 22 | 0.10 (0.00, 0.31) **^5^** | 61 / 1042 | 0.05 (0.03, 0.08) **^5^** | 3.76 (0.95, 14.82) | 0 (0.77) |  |
| Preterm labor with onset before 37w GA **^2^** | 3 | d j l | 0 / 9 | *incalculable* | 46 / 447 | 0.10 (0.07, 0.14) | 0.62 (0.02, 18.50) | 0 (0.88) |  |
| Cesarean Delivery | 7 | d h j l m1 m2 n | 5 / 28 | 0.18 (0.08, 0.36) | 410 / 1193 | 0.30 (0.24, 0.36) | 1.15 (0.54, 2.45) | 0 (0.99) |  |
| Intrapartum Cesarean Delivery | 6 | d j l m1* m2 n | 1 / 20 | 0.05 (0.01, 0.28) | 211 / 1023 | 0.17 (0.12, 0.23) | 1.42 (0.26, 7.78) | 0 (0.88) |  |
|  |  |  |  |  |  |  |  |  |  |
| Stillbirth **^3^** | 8 | d f h* j* l* m1* m2* n* | -- | -- | -- | -- | -- | -- |  |
| Perinatal death | 6 | d j* l* m1* m2 n* | -- | -- | -- | -- | -- | -- |  |
| Early neonatal death | 6 | d j* l* m1* m2 n* | -- | -- | -- | -- | -- | -- |  |
| Neonatal death **^4^** | 6 | d j* l* m1* m2 n* | -- | -- | -- | -- | -- | -- |  |
| NICU Admission at Birth | 4 | d m1 m2 n | 0 / 21 | *incalculable* | 35 / 943 | 0.04 (0.03, 0.05) | 2.21 (0.26, 18.78) | 0 (0.89) |  |
|  |  |  |  |  |  |  |  |  |  |
| Very low birthweight (<1500g) | 6 | d j* l m1 m2 n* | 1 / 21 | 0.09 (0.00, 0.29) **^5^** | 14 / 971 | 0.01 (0.01, 0.02) **^5^** | **14.81 (3.25, 67.39)** | 0 (0.73) |  |
| Low birthweight (<2500g) | 6 | d j l m1 m2 n | 3 / 27 | 0.11 (0.04, 0.29) | 104 / 1164 | 0.08 (0.06, 0.11) | 1.98 (0.74, 5.26) | 0 (0.91) |  |
| Small for gestational age (3rd) | 6 | d j* l m1 m2 n* | 2 / 21 | 0.10 (0.02, 0.31) | 23 / 971 | 0.02 (0.02, 0.04) | **7.14 (1.98, 25.73)** | 0 (0.41) |  |
| Small for gestational age (10th) | 6 | d j l m1 m2 n | 3 / 27 | 0.11 (0.04, 0.29) | 83 / 1164 | 0.08 (0.06, 0.10) | 2.46 (0.90, 6.70) | 0 (0.87) |  |
| Moderate preterm birth (<34w) | 6 | d j l m1 m2 n | 1 / 27 | 0.10 (0.00, 0.28) **^5^** | 36 / 1164 | 0.03 (0.02, 0.04) **^5^** | **7.53 (2.33, 24.29)** | 0 (0.96) |  |
| Moderate preterm birth (<34w) with onset before 34w GA **^2^** | 2 | d j | -- | -- | -- | -- | -- | -- |  |
| Preterm birth (<37 wks) | 7 | c2 d j l m1 m2 n | 3 / 44 | 0.07 (0.02, 0.19) | 178 / 1559 | 0.09 (0.06, 0.12) | 1.58 (0.59, 4.26) | 0 (0.84) |  |
| Preterm birth (<37 wks) with onset before 37w GA **^2^** | 2 | d j | -- | -- | -- | -- | -- | -- |  |
| *Notes: Pooled absolute risks are calculated using a logistic-normal random-effects meta-analysis, pooling all participating studies with at least 1 adverse event for the given outcome; in cases where the model does not converge, we instead calculate pooled absolute risk using a random effects model with Freeman-Tukey Double Arcsine Transformation. For any study with zero events in one arm at the study level (Risk Group or Reference Group), we apply a continuity correction of 0.5 for pooled absolute risks. For any outcome with zero events for all studies in a given risk or reference group, we consider the absolute risk to be incalculable based on the current data. Relative risks are calculated by pooling unadjusted relative risks from all participating studies with at least 1 adverse event for the given outcome using a DerSimonian-Laird random effects model meta-analysis. For any study with zero events in one arm (Risk Group or Reference Group), we used a continuity correction of the inverse of the number of events in the oppposite group within the same study. We present pooled estimates for analyses where at least three studies report the relevant data and report at least 1 adverse event for the outcome of interest.  1 This column lists the studies that provide data for the given estimate. Any study sites indicated with an asterisk (*) reported "0" events in among those with and without the given risk factor. These "0 event" studies are excluded from the estimate and from the "Events/Total" column. Studies are identified as follows: a) Martinez-Portilla, 2021, Mexico; b) Favre, Panchaud, 2021, multicountry COVI-Preg registry; c1) Money, 2020 – Maternal Subset, Canada; c2) Money, 2020 – Infant Subset, Canada; d) Carrillo, 2021, Chile; e) Knight, 2021, United Kingdom; f) Bracero, Valencia, Delgado-Lopez, 2021, Puerto Rico (USA); g) Sakowicz, 2020, USA (Chicago); h) Sanin, Mesa, Tolosa, 2021, Colombia; i) Nachega, 2021, multicountry AFREHealth study; j) Waldorf, Lokken, 2021, USA (Washington State); k) Divakar, 2021, India (Karnataka State); l) Gil, Fernandez Buhigas, 2021, Spain (Madrid); m1) Crovetto, 2020, Cohort I, Spain (Barcelona); m2) Crovetto, 2020, Cohort II, Spain (Barcelona); n) Bevilacqua, Laurita Longo, 2020, Italy (Rome); o) Nunes, 2021, South Africa; p) Akelo, Tippett Barr, 2021, Kenya; q) Yang, Juan, 2020, China; r) Kalafat, 2020, Turkey; s) Brandt, 2020, USA (New Brunswick); t) Poon, 2021, Hong Kong (China). 2 These outcomes (preterm labor, moderate preterm birth before 34 weeks gestation, and preterm birth before 37 weeks’ gestation) were included in the sensitivity analyses where we restrict confirmed COVID-19 cases to those with confirmed COVID-19 onset prior to 37 weeks’ gestation (or 34 weeks for very moderate preterm birth). The full comparison group is used for each of the sensitivity analyses.  3 The outcome presented here is stillbirths occurring at or after 28 weeks gestational age per the WHO definition.  4 The outcome "neonatal death" is reported by 15 participating studies. However, most studies were not designed to follow-up neonates until 28 days after birth. Therefore, counts of neonatal death are underestimated.  5 The pooled absolute risk presented are calculated using a random effects model with Freeman-Tukey Double Arcsine Transformation, which was a better fit for the data in this case.* | | | | | | | | |  |
|  |  |  |  |  |  |  |  |  |  |
|  |  |  |  |  |  |  |  |  |  |
|  |  |  |  |  |  |  |  |  |  |
|  |  |  |  |  |  |  |  |  |  |
|  |  |  |  |  |  |  |  |  |  |
|  |  |  |  |  |  |  |  |  |  |
|  |  |  |  |  |  |  |  |  |  |
|  |  |  |  |  |  |  |  |  |  |
|  |  |  |  |  |  |  |  |  |  |
|  |  |  |  |  |  |  |  |  |  |
|  |  |  |  |  |  |  |  |  |  |
|  |  |  |  |  |  |  |  |  |  |
|  |  |  |  |  |  |  |  |  |  |
|  |  |  |  |  |  |  |  |  |  |
|  |  |  |  |  |  |  |  |  |  |

Table S13. Risk of Outcomes among COVID+ pregnancy, stratified by Hgb <110 g/L

| Outcome | N Studies | Included Studies **^1^** | With Hgb < 110 g/L | | Without Hgb < 110 g/L | | Pooled, unadjusted RR (95% CI) | I^2 (pvalue) |  |
| --- | --- | --- | --- | --- | --- | --- | --- | --- | --- |
|  |  |  | Events/Total | Pooled Risk (95% CI) | Events/Total | Pooled Risk (95% CI) |  |  |  |
|  |  |  |  |  |  |  |  |  |  |
| ICU admission | 4 | b h i t* | 79 / 325 | 0.28 (0.16, 0.45) | 96 / 764 | 0.15 (0.09, 0.24) | **1.67 (1.28, 2.19)** | 0 (0.96) |  |
| Ventilation | 4 | b i k t* | 22 / 304 | 0.07 (0.04, 0.10) | 26 / 670 | 0.04 (0.02, 0.05) | **1.78 (1.02, 3.12)** | 0 (0.76) |  |
| Critical Care | 3 | b i t* | -- | -- | -- | -- | -- | -- |  |
| Pneumonia | 2 | b t* | -- | -- | -- | -- | -- | -- |  |
| Pregnancy-related death | 5 | b h i k* t* | 23 / 230 | 12603.19 (2739.89, 42469.08) | 19 / 579 | 4354.98 (1731.34, 10528.47) | **2.36 (1.15, 4.81)** | 27.20 (0.25) |  |
|  |  |  |  |  |  |  |  |  |  |
| Haemorrhage | 2 | h i | -- | -- | -- | -- | -- | -- |  |
| Placental Abruption | 2 | i t* | -- | -- | -- | -- | -- | -- |  |
| Preeclampsia | 3 | b k t* | -- | -- | -- | -- | -- | -- |  |
| Preeclampsia or Eclampsia | 3 | i k t* | -- | -- | -- | -- | -- | -- |  |
| Hypertensive Disorders of Pregnancy (Any) | 3 | i k t | 20 / 94 | 0.21 (0.14, 0.31) | 33 / 125 | 0.22 (0.09, 0.43) | 0.87 (0.52, 1.46) | 0 (0.58) |  |
| Hypertensive Disorders of Pregnancy (At/After Covid-19) | 1 | t | -- | -- | -- | -- | -- | -- |  |
| Preterm labor | 2 | k* t | -- | -- | -- | -- | -- | -- |  |
| Preterm labor with onset before 37w GA **^2^** | 2 | k* t | -- | -- | -- | -- | -- | -- |  |
| Cesarean Delivery | 4 | b h k t | 69 / 211 | 0.29 (0.19, 0.42) | 210 / 514 | 0.43 (0.35, 0.51) | 0.75 (0.47, 1.19) | 38.54 (0.18) |  |
| Intrapartum Cesarean Delivery | 3 | b k t | 42 / 196 | 0.16 (0.06, 0.34) | 110 / 443 | 0.25 (0.21, 0.29) | 0.67 (0.28, 1.62) | 53.88 (0.11) |  |
|  |  |  |  |  |  |  |  |  |  |
| Stillbirth **^3^** | 5 | b h* i k t* | 7 / 228 | 33.56 (12.70, 85.69) | 3 / 520 | 5.77 (1.86, 17.73) | **3.75 (1.00, 14.11)** | 0 (0.89) |  |
| Perinatal death | 3 | b k t* | -- | -- | -- | -- | -- | -- |  |
| Early neonatal death | 3 | b k* t* | -- | -- | -- | -- | -- | -- |  |
| Neonatal death **^4^** | 4 | b i k t* | 3 / 220 | 13.64 (4.40, 41.41) | 1 / 516 | 1.94 (0.27, 13.62) | 2.98 (0.49, 18.13) | 0 (0.98) |  |
| NICU Admission at Birth | 2 | b t | -- | -- | -- | -- | -- | -- |  |
|  |  |  |  |  |  |  |  |  |  |
| Very low birthweight (<1500g) | 4 | b i k t* | 9 / 220 | 0.04 (0.02, 0.10) | 14 / 516 | 0.03 (0.02, 0.05) | 1.64 (0.47, 5.73) | 28.69 (0.25) |  |
| Low birthweight (<2500g) | 4 | b i k t | 38 / 226 | 0.19 (0.12, 0.29) | 99 / 519 | 0.19 (0.16, 0.23) | 0.99 (0.60, 1.62) | 30.33 (0.23) |  |
| Small for gestational age (3rd) | 4 | b i k t* | 13 / 220 | 0.05 (0.01, 0.23) | 21 / 516 | 0.05 (0.02, 0.15) | 1.11 (0.56, 2.21) | 0 (0.55) |  |
| Small for gestational age (10th) | 4 | b i k t* | 27 / 220 | 0.14 (0.06, 0.31) | 56 / 516 | 0.14 (0.07, 0.24) | 0.99 (0.64, 1.53) | 0 (0.74) |  |
| Moderate preterm birth (<34w) | 4 | b i k t* | 15 / 220 | 0.06 (0.02, 0.17) | 43 / 516 | 0.08 (0.06, 0.11) | 0.91 (0.51, 1.61) | 0 (0.49) |  |
| Moderate preterm birth (<34w) with onset before 34w GA **^2^** | 2 | b t* | -- | -- | -- | -- | -- | -- |  |
| Preterm birth (<37 wks) | 4 | b i k t | 39 / 226 | 0.17 (0.11, 0.27) | 98 / 519 | 0.19 (0.16, 0.22) | 0.94 (0.67, 1.32) | 0 (0.88) |  |
| Preterm birth (<37 wks) with onset before 37w GA **^2^** | 3 | b k t | 25 / 98 | 0.38 (0.08, 0.81) | 75 / 253 | 0.34 (0.18, 0.55) | 0.92 (0.62, 1.37) | 0 (0.77) |  |
| *Notes: Pooled absolute risks are calculated using a logistic-normal random-effects meta-analysis, pooling all participating studies with at least 1 adverse event for the given outcome; in cases where the model does not converge, we instead calculate pooled absolute risk using a random effects model with Freeman-Tukey Double Arcsine Transformation. For any study with zero events in one arm at the study level (Risk Group or Reference Group), we apply a continuity correction of 0.5 for pooled absolute risks. For any outcome with zero events for all studies in a given risk or reference group, we consider the absolute risk to be incalculable based on the current data. Relative risks are calculated by pooling unadjusted relative risks from all participating studies with at least 1 adverse event for the given outcome using a DerSimonian-Laird random effects model meta-analysis. For any study with zero events in one arm (Risk Group or Reference Group), we used a continuity correction of the inverse of the number of events in the oppposite group within the same study. We present pooled estimates for analyses where at least three studies report the relevant data and report at least 1 adverse event for the outcome of interest.  1 This column lists the studies that provide data for the given estimate. Any study sites indicated with an asterisk (*) reported "0" events in among those with and without the given risk factor. These "0 event" studies are excluded from the estimate and from the "Events/Total" column. Studies are identified as follows: a) Martinez-Portilla, 2021, Mexico; b) Favre, Panchaud, 2021, multicountry COVI-Preg registry; c1) Money, 2020 – Maternal Subset, Canada; c2) Money, 2020 – Infant Subset, Canada; d) Carrillo, 2021, Chile; e) Knight, 2021, United Kingdom; f) Bracero, Valencia, Delgado-Lopez, 2021, Puerto Rico (USA); g) Sakowicz, 2020, USA (Chicago); h) Sanin, Mesa, Tolosa, 2021, Colombia; i) Nachega, 2021, multicountry AFREHealth study; j) Waldorf, Lokken, 2021, USA (Washington State); k) Divakar, 2021, India (Karnataka State); l) Gil, Fernandez Buhigas, 2021, Spain (Madrid); m1) Crovetto, 2020, Cohort I, Spain (Barcelona); m2) Crovetto, 2020, Cohort II, Spain (Barcelona); n) Bevilacqua, Laurita Longo, 2020, Italy (Rome); o) Nunes, 2021, South Africa; p) Akelo, Tippett Barr, 2021, Kenya; q) Yang, Juan, 2020, China; r) Kalafat, 2020, Turkey; s) Brandt, 2020, USA (New Brunswick); t) Poon, 2021, Hong Kong (China). 2 These outcomes (preterm labor, moderate preterm birth before 34 weeks gestation, and preterm birth before 37 weeks’ gestation) were included in the sensitivity analyses where we restrict confirmed COVID-19 cases to those with confirmed COVID-19 onset prior to 37 weeks’ gestation (or 34 weeks for very moderate preterm birth). The full comparison group is used for each of the sensitivity analyses.  3 The outcome presented here is stillbirths occurring at or after 28 weeks gestational age per the WHO definition.  4 The outcome "neonatal death" is reported by 15 participating studies. However, most studies were not designed to follow-up neonates until 28 days after birth. Therefore, counts of neonatal death are underestimated.* | | | | | | | | |  |
|  |  |  |  |  |  |  |  |  |  |
|  |  |  |  |  |  |  |  |  |  |
|  |  |  |  |  |  |  |  |  |  |
|  |  |  |  |  |  |  |  |  |  |
|  |  |  |  |  |  |  |  |  |  |
|  |  |  |  |  |  |  |  |  |  |
|  |  |  |  |  |  |  |  |  |  |
|  |  |  |  |  |  |  |  |  |  |
|  |  |  |  |  |  |  |  |  |  |
|  |  |  |  |  |  |  |  |  |  |
|  |  |  |  |  |  |  |  |  |  |
|  |  |  |  |  |  |  |  |  |  |
|  |  |  |  |  |  |  |  |  |  |
|  |  |  |  |  |  |  |  |  |  |
|  |  |  |  |  |  |  |  |  |  |

Table S14. Risk of Outcomes among COVID+ pregnancy, stratified by maternal age (20-34, >35) - ref group = age 20-34

| Outcome | N Studies | Included Studies **^1^** | Maternal Age 35-45 | | Maternal Age 20-34 | |  | |  |
| --- | --- | --- | --- | --- | --- | --- | --- | --- | --- |
|  |  |  | Events/Total | Pooled Risk (95% CI) | Events/Total | Pooled Risk (95% CI) | Pooled, unadjusted RR (95% CI) | I^2 (pvalue) |  |
|  |  |  |  |  |  |  |  |  |  |
| ICU admission | 16 | a b c1 d e g i j l* m1* m2 n q r s t | 200 / 4154 | 0.05 (0.03, 0.08) | 411 / 14604 | 0.03 (0.01, 0.04) | **1.60 (1.36, 1.89)** | 0 (0.72) |  |
| Ventilation | 16 | a b c1 d e i j k l m1* m2 n q r s t | 105 / 4043 | 0.03 (0.02, 0.04) | 181 / 14364 | 0.01 (0.01, 0.02) | **2.13 (1.68, 2.71)** | 0 (0.93) |  |
| Critical Care | 15 | a b c1 d e i j l m1* m2 n q r s t* | 215 / 4085 | 0.05 (0.03, 0.08) | 445 / 14367 | 0.03 (0.02, 0.05) | **1.62 (1.38, 1.90)** | 0 (0.58) |  |
| Pneumonia | 10 | a b d e l m1* m2 n r t* | 375 / 3424 | 0.12 (0.04, 0.31) | 884 / 12246 | 0.06 (0.03, 0.14) | **1.51 (1.35, 1.70)** | 0 (0.86) |  |
| Pregnancy-related death | 16 | a b d e i j k* l* m1* m2* n* o q* r* s* t* | 74 / 3107 | 2066.46 (679.77, 6107.97) | 122 / 11559 | 956.79 (282.46, 3189.51) | 1.62 (0.81, 3.24) | 55.94 (0.03) |  |
|  |  |  |  |  |  |  |  |  |  |
| Haemorrhage | 6 | g i j l n o | 41 / 394 | 0.10 (0.05, 0.17) | 86 / 861 | 0.09 (0.04, 0.16) | 1.17 (0.82, 1.68) | 0 (0.72) |  |
| Placental Abruption | 6 | g i j l r* t | 10 / 303 | 0.03 (0.02, 0.08) | 4 / 690 | 0.00 (0.00, 0.03) | **3.94 (1.40, 11.13)** | 0 (0.92) |  |
| Preeclampsia | 13 | b d g k l m1 m2 o p q r s t | 57 / 1183 | 0.05 (0.02, 0.09) | 159 / 3264 | 0.05 (0.03, 0.07) | 1.12 (0.73, 1.74) | 25.59 (0.19) |  |
| Preeclampsia or Eclampsia | 9 | g i k m1 m2 n o p t | 35 / 452 | 0.07 (0.04, 0.12) | 111 / 1115 | 0.06 (0.03, 0.13) | 0.93 (0.63, 1.37) | 0 (0.48) |  |
| Hypertensive Disorders of Pregnancy (Any) | 12 | d g i k l m1 m2 n o p q t | 88 / 754 | 0.10 (0.07, 0.15) | 222 / 2109 | 0.08 (0.05, 0.13) | 1.17 (0.93, 1.49) | 0 (0.59) |  |
| Hypertensive Disorders of Pregnancy (At/After Covid-19) | 3 | l p t | 8 / 87 | 0.09 (0.05, 0.17) | 8 / 173 | 0.03 (0.01, 0.15) | 1.91 (0.45, 8.16) | 36.13 (0.21) |  |
| Preterm labor | 10 | d j k l m1* m2 n o q t | 41 / 531 | 0.08 (0.05, 0.14) | 86 / 1606 | 0.05 (0.03, 0.08) | 1.39 (0.96, 2.02) | 0 (0.90) |  |
| Preterm labor with onset before 37w GA **^2^** | 8 | d j k l n o q t | 33 / 271 | 0.15 (0.06, 0.33) | 76 / 741 | 0.09 (0.04, 0.20) | 1.28 (0.87, 1.87) | 0 (0.94) |  |
| Cesarean Delivery | 13 | b d g j k l m1 m2 n o q s t | 508 / 1210 | 0.43 (0.36, 0.50) | 1158 / 3294 | 0.35 (0.25, 0.47) | **1.21 (1.10, 1.32)** | 0 (0.83) |  |
| Intrapartum Cesarean Delivery | 10 | b d j k l m1* m2 n o t | 205 / 973 | 0.22 (0.17, 0.27) | 594 / 2738 | 0.21 (0.15, 0.28) | 1.03 (0.89, 1.20) | 0 (0.80) |  |
|  |  |  |  |  |  |  |  |  |  |
| Stillbirth **^3^** | 18 | b d e f g i j k l* m1 m2* n* o p q* r* s* t* | 13 / 1428 | 9.16 (3.93, 21.18) | 30 / 4334 | 7.53 (4.38, 12.92) | 1.75 (0.92, 3.33) | 0 (0.91) |  |
| Perinatal death | 14 | b d e j k l* m1 m2 n* o q r* s* t* | 14 / 1251 | 11.19 (6.64, 18.81) | 31 / 3414 | 9.08 (6.39, 12.88) | 1.53 (0.82, 2.83) | 0 (0.92) |  |
| Early neonatal death | 14 | b d e j* k* l* m1* m2 n* o* q r* s* t* | 4 / 1087 | 2.97 (0.61, 14.24) | 10 / 2907 | 3.44 (1.85, 6.38) | 1.80 (0.51, 6.33) | 0 (0.56) |  |
| Neonatal death **^4^** | 15 | b d e i j* k l* m1* m2 n* o* q r* s* t* | 5 / 1134 | 2.72 (0.40, 18.02) | 15 / 3200 | 4.69 (2.83, 7.76) | 1.96 (0.65, 5.87) | 0 (0.52) |  |
| NICU Admission at Birth | 9 | b d m1 m2 n q r s t | 132 / 931 | 0.17 (0.06, 0.41) | 258 / 2560 | 0.12 (0.04, 0.31) | **1.35 (1.12, 1.63)** | 0 (0.79) |  |
|  |  |  |  |  |  |  |  |  |  |
| Very low birthweight (<1500g) | 16 | b d g i j* k l m1 m2 n o p* q* r* s t* | 30 / 1206 | 0.02 (0.02, 0.04) | 64 / 3218 | 0.02 (0.01, 0.03) | 1.39 (0.89, 2.16) | 0 (0.95) |  |
| Low birthweight (<2500g) | 16 | b d g i j k l m1 m2 n o p q r s t | 168 / 1279 | 0.13 (0.09, 0.17) | 390 / 3556 | 0.10 (0.07, 0.14) | **1.24 (1.04, 1.47)** | 0 (0.89) |  |
| Small for gestational age (3rd) | 17 | b d e g i j* k l m1 m2 n o p q r s* t | 47 / 1536 | 0.03 (0.01, 0.06) | 118 / 4051 | 0.02 (0.01, 0.04) | **1.46 (1.01, 2.12)** | 0 (0.71) |  |
| Small for gestational age (10th) | 17 | b d e g i j k l m1 m2 n o p q r s t | 113 / 1584 | 0.07 (0.04, 0.10) | 357 / 4212 | 0.09 (0.06, 0.12) | 0.98 (0.79, 1.21) | 0 (0.91) |  |
| Moderate preterm birth (<34w) | 17 | b d e g i j k l m1 m2 n o p q r s t | 98 / 1584 | 0.05 (0.04, 0.08) | 183 / 4212 | 0.04 (0.03, 0.06) | **1.51 (1.19, 1.93)** | 0 (0.93) |  |
| Moderate preterm birth (<34w) with onset before 34w GA **^2^** | 10 | b d g j k n o p s t | 64 / 494 | 0.16 (0.10, 0.24) | 119 / 1302 | 0.11 (0.07, 0.17) | **1.43 (1.07, 1.90)** | 0 (1.00) |  |
| Preterm birth (<37 wks) | 18 | b c2 d e g i j k l m1 m2 n o p q r s t | 310 / 1786 | 0.18 (0.14, 0.22) | 606 / 4790 | 0.12 (0.09, 0.16) | **1.40 (1.19, 1.64)** | 15.77 (0.26) |  |
| Preterm birth (<37 wks) with onset before 37w GA **^2^** | 11 | b d g j k n o p q s t | 164 / 650 | 0.39 (0.23, 0.59) | 374 / 1789 | 0.28 (0.16, 0.44) | **1.27 (1.07, 1.50)** | 0 (0.49) |  |
| *Notes: Pooled absolute risks are calculated using a logistic-normal random-effects meta-analysis, pooling all participating studies with at least 1 adverse event for the given outcome; in cases where the model does not converge, we instead calculate pooled absolute risk using a random effects model with Freeman-Tukey Double Arcsine Transformation. For any study with zero events in one arm at the study level (Risk Group or Reference Group), we apply a continuity correction of 0.5 for pooled absolute risks. For any outcome with zero events for all studies in a given risk or reference group, we consider the absolute risk to be incalculable based on the current data. Relative risks are calculated by pooling unadjusted relative risks from all participating studies with at least 1 adverse event for the given outcome using a DerSimonian-Laird random effects model meta-analysis. For any study with zero events in one arm (Risk Group or Reference Group), we used a continuity correction of the inverse of the number of events in the oppposite group within the same study. We present pooled estimates for analyses where at least three studies report the relevant data and report at least 1 adverse event for the outcome of interest.  1 This column lists the studies that provide data for the given estimate. Any study sites indicated with an asterisk (*) reported "0" events in among those with and without the given risk factor. These "0 event" studies are excluded from the estimate and from the "Events/Total" column. Studies are identified as follows: a) Martinez-Portilla, 2021, Mexico; b) Favre, Panchaud, 2021, multicountry COVI-Preg registry; c1) Money, 2020 – Maternal Subset, Canada; c2) Money, 2020 – Infant Subset, Canada; d) Carrillo, 2021, Chile; e) Knight, 2021, United Kingdom; f) Bracero, Valencia, Delgado-Lopez, 2021, Puerto Rico (USA); g) Sakowicz, 2020, USA (Chicago); h) Sanin, Mesa, Tolosa, 2021, Colombia; i) Nachega, 2021, multicountry AFREHealth study; j) Waldorf, Lokken, 2021, USA (Washington State); k) Divakar, 2021, India (Karnataka State); l) Gil, Fernandez Buhigas, 2021, Spain (Madrid); m1) Crovetto, 2020, Cohort I, Spain (Barcelona); m2) Crovetto, 2020, Cohort II, Spain (Barcelona); n) Bevilacqua, Laurita Longo, 2020, Italy (Rome); o) Nunes, 2021, South Africa; p) Akelo, Tippett Barr, 2021, Kenya; q) Yang, Juan, 2020, China; r) Kalafat, 2020, Turkey; s) Brandt, 2020, USA (New Brunswick); t) Poon, 2021, Hong Kong (China). 2 These outcomes (preterm labor, moderate preterm birth before 34 weeks gestation, and preterm birth before 37 weeks’ gestation) were included in the sensitivity analyses where we restrict confirmed COVID-19 cases to those with confirmed COVID-19 onset prior to 37 weeks’ gestation (or 34 weeks for very moderate preterm birth). The full comparison group is used for each of the sensitivity analyses.  3 The outcome presented here is stillbirths occurring at or after 28 weeks gestational age per the WHO definition.  4 The outcome "neonatal death" is reported by 15 participating studies. However, most studies were not designed to follow-up neonates until 28 days after birth. Therefore, counts of neonatal death are underestimated.  5 The pooled absolute risk presented are calculated using a random effects model with Freeman-Tukey Double Arcsine Transformation, which was a better fit for the data in this case.* | | | | | | | | |  |
|  |  |  |  |  |  |  |  |  |  |
|  |  |  |  |  |  |  |  |  |  |
|  |  |  |  |  |  |  |  |  |  |
|  |  |  |  |  |  |  |  |  |  |
|  |  |  |  |  |  |  |  |  |  |
|  |  |  |  |  |  |  |  |  |  |
|  |  |  |  |  |  |  |  |  |  |
|  |  |  |  |  |  |  |  |  |  |
|  |  |  |  |  |  |  |  |  |  |
|  |  |  |  |  |  |  |  |  |  |
|  |  |  |  |  |  |  |  |  |  |
|  |  |  |  |  |  |  |  |  |  |
|  |  |  |  |  |  |  |  |  |  |
|  |  |  |  |  |  |  |  |  |  |
|  |  |  |  |  |  |  |  |  |  |

Table S15. Risk of Outcomes among COVID+ pregnancy, stratified by maternal age (<20, 20-34) - ref group = age 20-34

| Outcome | N Studies | Included Studies **^1^** | Maternal Age 15-19 | | Maternal Age 20-34 | | Pooled, unadjusted RR (95% CI) |  |  |
| --- | --- | --- | --- | --- | --- | --- | --- | --- | --- |
|  |  |  | Events/Total | Pooled Risk (95% CI) | Events/Total | Pooled Risk (95% CI) |  | I^2 (pvalue) |  |
|  |  |  |  |  |  |  |  |  |  |
| ICU admission | 12 | a b d e g i j l* m1* m2 n* r | 16 / 858 | 0.02 (0.01, 0.03) | 381 / 12789 | 0.03 (0.02, 0.06) | 1.42 (0.53, 3.77) | 48.53 (0.05) |  |
| Ventilation | 12 | a b d e i j k l* m1* m2 n r | 6 / 845 | 0.01 (0.00, 0.02) | 170 / 12749 | 0.01 (0.01, 0.02) | 2.59 (0.79, 8.51) | 42.65 (0.07) |  |
| Critical Care | 11 | a b d e i j l* m1* m2 n r | 16 / 842 | 0.02 (0.01, 0.03) | 415 / 12553 | 0.04 (0.02, 0.06) | 1.24 (0.48, 3.17) | 38.15 (0.11) |  |
| Pneumonia | 9 | a b d e l m1* m2 n r | 47 / 827 | 0.03 (0.00, 0.18) | 884 / 12246 | 0.06 (0.03, 0.14) | 0.82 (0.62, 1.08) | 0 (0.81) |  |
| Pregnancy-related death | 13 | a b d e i j* k* l* m1* m2* n* o r* | 3 / 801 | 0.00 (0.00, 1235.35) | 122 / 11440 | 1684.94 (547.12, 3342.26) | 0.73 (0.27, 1.94) | 0 (0.69) |  |
|  |  |  |  |  |  |  |  |  |  |
| Haemorrhage | 6 | g i j l* n o | 6 / 33 | 0.20 (0.07, 0.44) | 86 / 765 | 0.12 (0.09, 0.16) | 1.93 (0.94, 3.98) | 0 (0.73) |  |
| Placental Abruption | 5 | g i j* l* r* | -- | -- | -- | -- | -- | -- |  |
| Preeclampsia | 10 | b d g k l m1 m2 o p* r | 8 / 130 | 0.08 (0.00, 0.21) **^5^** | 152 / 3061 | 0.06 (0.03, 0.09) **^5^** | 2.03 (0.89, 4.61) | 25.94 (0.21) |  |
| Preeclampsia or Eclampsia | 8 | g i k m1 m2 n o p* | 5 / 36 | 0.11 (0.02, 0.46) | 110 / 1038 | 0.08 (0.04, 0.15) | **3.27 (1.11, 9.64)** | 31.08 (0.19) |  |
| Hypertensive Disorders of Pregnancy (Any) | 10 | d g i k l m1 m2 n o p | 9 / 118 | 0.10 (0.00, 0.25) **^5^** | 217 / 2014 | 0.09 (0.06, 0.14) **^5^** | 2.06 (0.77, 5.55) | 45.56 (0.06) |  |
| Hypertensive Disorders of Pregnancy (At/After Covid-19) | 2 | l p* | -- | -- | -- | -- | -- | -- |  |
| Preterm labor | 8 | d j k l m1* m2 n o | 2 / 98 | 0.04 (0.00, 0.20) **^5^** | 70 / 1511 | 0.04 (0.02, 0.06) **^5^** | 2.48 (0.53, 11.60) | 44.63 (0.09) |  |
| Preterm labor with onset before 37w GA **^2^** | 5 | d j k l o | 2 / 46 | 0.04 (0.01, 0.16) | 60 / 678 | 0.08 (0.05, 0.13) | 1.62 (0.42, 6.22) | 23.17 (0.27) |  |
| Cesarean Delivery | 10 | b d g j k l m1 m2 n o | 40 / 132 | 0.30 (0.23, 0.39) | 1071 / 3158 | 0.31 (0.24, 0.40) | 0.86 (0.65, 1.13) | 0 (0.99) |  |
| Intrapartum Cesarean Delivery | 9 | b d j k l m1* m2 n o | 24 / 114 | 0.21 (0.15, 0.29) | 591 / 2727 | 0.20 (0.14, 0.28) | 0.90 (0.63, 1.31) | 0 (0.99) |  |
|  |  |  |  |  |  |  |  |  |  |
| Stillbirth **^3^** | 15 | b d e f g i j k l* m1* m2* n* o p r | 2 / 227 | 8.81 (2.20, 34.53) | 30 / 4302 | 7.64 (4.45, 13.11) | **4.59 (1.69, 12.45)** | 0 (0.99) |  |
| Perinatal death | 11 | b d e j k l* m1* m2 n* o r | 1 / 139 | 11.05 (0.00, 72.76) | 30 / 3298 | 6.89 (3.89, 10.51) | **4.80 (1.28, 17.99)** | 0 (0.80) |  |
| Early neonatal death | 11 | b d e j* k* l* m1* m2 n* o* r* | 0 / 120 | *incalculable* | 9 / 2823 | 3.19 (1.66, 6.11) | **5.94 (1.02, 34.56)** | 0 (0.74) |  |
| Neonatal death **^4^** | 12 | b d e i j* k l* m1* m2 n* o* r* | 0 / 126 | *incalculable* | 14 / 3116 | 4.49 (2.66, 7.57) | **9.38 (2.21, 39.89)** | 0 (0.75) |  |
| NICU Admission at Birth | 6 | b d m1 m2 n r | 3 / 103 | 0.03 (0.01, 0.09) | 181 / 2423 | 0.05 (0.03, 0.11) | 1.59 (0.48, 5.23) | 22.82 (0.26) |  |
|  |  |  |  |  |  |  |  |  |  |
| Very low birthweight (<1500g) | 13 | b d g i j* k l m1 m2 n* o p* r* | 2 / 131 | 0.02 (0.00, 0.11) **5** | 63 / 3072 | 0.02 (0.01, 0.03) **^5^** | **6.27 (1.86, 21.15)** | 0 (0.65) |  |
| Low birthweight (<2500g) | 13 | b d g i j k l m1 m2 n o p r | 10 / 144 | 0.07 (0.04, 0.12) | 379 / 3419 | 0.10 (0.07, 0.15) | 0.96 (0.54, 1.73) | 0 (0.72) |  |
| Small for gestational age (3rd) | 14 | b d e g i j* k l m1 m2* n* o p r | 4 / 155 | 0.03 (0.00, 0.11) **^5^** | 115 / 3746 | 0.03 (0.01, 0.06) **^5^** | **4.33 (1.87, 10.06)** | 0 (0.65) |  |
| Small for gestational age (10th) | 14 | b d e g i j k l m1 m2 n o p r | 12 / 166 | 0.08 (0.04, 0.15) | 349 / 4075 | 0.09 (0.06, 0.13) | 1.40 (0.83, 2.36) | 0 (0.91) |  |
| Moderate preterm birth (<34w) | 14 | b d e g i j k l m1 m2 n o p r | 5 / 166 | 0.03 (0.01, 0.09) | 178 / 4075 | 0.04 (0.03, 0.06) | **3.06 (1.48, 6.35)** | 0 (0.80) |  |
| Moderate preterm birth (<34w) with onset before 34w GA **^2^** | 7 | b d g j k o p | 3 / 42 | 0.13 (0.00, 0.44) **5** | 116 / 1279 | 0.11 (0.06, 0.16) **^5^** | **2.90 (1.18, 7.14)** | 0 (0.92) |  |
| Preterm birth (<37 wks) | 14 | b d e g i j k l m1 m2 n o p r | 24 / 166 | 0.14 (0.10, 0.21) | 520 / 4075 | 0.12 (0.09, 0.17) | 1.22 (0.84, 1.78) | 0 (0.84) |  |
| Preterm birth (<37 wks) with onset before 37w GA **^2^** | 7 | b d g j k o p | 16 / 64 | 0.25 (0.16, 0.37) | 348 / 1725 | 0.25 (0.14, 0.40) | 1.06 (0.68, 1.67) | 0 (0.69) |  |
| *Notes: Pooled absolute risks are calculated using a logistic-normal random-effects meta-analysis, pooling all participating studies with at least 1 adverse event for the given outcome; in cases where the model does not converge, we instead calculate pooled absolute risk using a random effects model with Freeman-Tukey Double Arcsine Transformation. For any study with zero events in one arm at the study level (Risk Group or Reference Group), we apply a continuity correction of 0.5 for pooled absolute risks. For any outcome with zero events for all studies in a given risk or reference group, we consider the absolute risk to be incalculable based on the current data. Relative risks are calculated by pooling unadjusted relative risks from all participating studies with at least 1 adverse event for the given outcome using a DerSimonian-Laird random effects model meta-analysis. For any study with zero events in one arm (Risk Group or Reference Group), we used a continuity correction of the inverse of the number of events in the oppposite group within the same study. We present pooled estimates for analyses where at least three studies report the relevant data and report at least 1 adverse event for the outcome of interest.  1 This column lists the studies that provide data for the given estimate. Any study sites indicated with an asterisk (*) reported "0" events in among those with and without the given risk factor. These "0 event" studies are excluded from the estimate and from the "Events/Total" column. Studies are identified as follows: a) Martinez-Portilla, 2021, Mexico; b) Favre, Panchaud, 2021, multicountry COVI-Preg registry; c1) Money, 2020 – Maternal Subset, Canada; c2) Money, 2020 – Infant Subset, Canada; d) Carrillo, 2021, Chile; e) Knight, 2021, United Kingdom; f) Bracero, Valencia, Delgado-Lopez, 2021, Puerto Rico (USA); g) Sakowicz, 2020, USA (Chicago); h) Sanin, Mesa, Tolosa, 2021, Colombia; i) Nachega, 2021, multicountry AFREHealth study; j) Waldorf, Lokken, 2021, USA (Washington State); k) Divakar, 2021, India (Karnataka State); l) Gil, Fernandez Buhigas, 2021, Spain (Madrid); m1) Crovetto, 2020, Cohort I, Spain (Barcelona); m2) Crovetto, 2020, Cohort II, Spain (Barcelona); n) Bevilacqua, Laurita Longo, 2020, Italy (Rome); o) Nunes, 2021, South Africa; p) Akelo, Tippett Barr, 2021, Kenya; q) Yang, Juan, 2020, China; r) Kalafat, 2020, Turkey; s) Brandt, 2020, USA (New Brunswick); t) Poon, 2021, Hong Kong (China). 2 These outcomes (preterm labor, moderate preterm birth before 34 weeks gestation, and preterm birth before 37 weeks’ gestation) were included in the sensitivity analyses where we restrict confirmed COVID-19 cases to those with confirmed COVID-19 onset prior to 37 weeks’ gestation (or 34 weeks for very moderate preterm birth). The full comparison group is used for each of the sensitivity analyses.  3 The outcome presented here is stillbirths occurring at or after 28 weeks gestational age per the WHO definition.  4 The outcome "neonatal death" is reported by 15 participating studies. However, most studies were not designed to follow-up neonates until 28 days after birth. Therefore, counts of neonatal death are underestimated.  5 The pooled absolute risk presented are calculated using a random effects model with Freeman-Tukey Double Arcsine Transformation, which was a better fit for the data in this case.* | | | | | | | | |  |
|  |  |  |  |  |  |  |  |  |  |
|  |  |  |  |  |  |  |  |  |  |
|  |  |  |  |  |  |  |  |  |  |
|  |  |  |  |  |  |  |  |  |  |
|  |  |  |  |  |  |  |  |  |  |
|  |  |  |  |  |  |  |  |  |  |
|  |  |  |  |  |  |  |  |  |  |
|  |  |  |  |  |  |  |  |  |  |
|  |  |  |  |  |  |  |  |  |  |
|  |  |  |  |  |  |  |  |  |  |
|  |  |  |  |  |  |  |  |  |  |
|  |  |  |  |  |  |  |  |  |  |
|  |  |  |  |  |  |  |  |  |  |
|  |  |  |  |  |  |  |  |  |  |
|  |  |  |  |  |  |  |  |  |  |

Table S16. Risk of Outcomes among COVID+ pregnancy, stratified by primiparity (first birth)

| Outcome | N Studies | Included Studies **^1^** | With primiparity | | Without primiparity | | Pooled, unadjusted RR (95% CI) | I^2 (pvalue) |  |
| --- | --- | --- | --- | --- | --- | --- | --- | --- | --- |
|  |  |  | Events/Total | Pooled Risk (95% CI) | Events/Total | Pooled Risk (95% CI) |  |  |  |
|  |  |  |  |  |  |  |  |  |  |
| ICU admission | 14 | b e g h i j l* m1* m2 n q r s t | 101 / 2035 | 0.02 (0.01, 0.06) | 207 / 3572 | 0.05 (0.02, 0.09) | 0.90 (0.71, 1.13) | 0 (0.86) |  |
| Ventilation | 12 | b e i j l m1* m2 n q r s t | 14 / 1763 | 0.01 (0, 0.02) | 67 / 3226 | 0.02 (0.01, 0.04) | 0.67 (0.39, 1.16) | 0 (0.96) |  |
| Critical Care | 12 | b e i j l m1* m2 n q r s t* | 65 / 1753 | 0.01 (0, 0.05) | 176 / 3211 | 0.04 (0.02, 0.08) | 0.82 (0.62, 1.08) | 0 (0.75) |  |
| Pneumonia | 8 | b e l m1* m2 n r t* | 68 / 1561 | 0.04 (0.01, 0.13) | 206 / 2688 | 0.10 (0.03, 0.27) | **0.59 (0.46, 0.77)** | 0 (0.84) |  |
| Pregnancy-related death | 14 | b e h i j l* m1* m2* n* o q* r* s* t* | 20 / 1263 | 1833.91 (429.04, 7492.74) | 54 / 2282 | 2837.03 (938.75, 8254.08) | 0.75 (0.45, 1.25) | 0 (0.79) |  |
|  |  |  |  |  |  |  |  |  |  |
| Haemorrhage | 7 | g h i j l n o | 52 / 524 | 0.09 (0.05, 0.16) | 86 / 931 | 0.08 (0.04, 0.15) | 1.26 (0.90, 1.77) | 0 (0.43) |  |
| Placental Abruption | 6 | g i j l r* t | 2 / 348 | 0.01 (0.00, 0.02) | 13 / 674 | 0.02 (0.01, 0.04) | 0.64 (0.19, 2.09) | 0 (0.99) |  |
| Preeclampsia | 11 | b g l m1 m2 o p q r s t | 74 / 1225 | 0.06 (0.04, 0.10) | 51 / 2055 | 0.03 (0.02, 0.05) | **2.10 (1.45, 3.03)** | 0 (0.91) |  |
| Preeclampsia or Eclampsia | 8 | g i m1 m2 n o p t | 63 / 553 | 0.07 (0.03, 0.17) | 57 / 841 | 0.05 (0.02, 0.10) | **1.75 (1.22, 2.53)** | 0 (0.61) |  |
| Hypertensive Disorders of Pregnancy (Any) | 10 | g i l m1 m2 n o p q t | 90 / 681 | 0.08 (0.04, 0.16) | 95 / 984 | 0.07 (0.04, 0.12) | **1.56 (1.13, 2.15)** | 6.55 (0.38) |  |
| Hypertensive Disorders of Pregnancy (At/After Covid-19) | 3 | l p t | 8 / 105 | 0.08 (0.04, 0.15) | 9 / 165 | 0.06 (0.02, 0.13) | 1.39 (0.54, 3.57) | 0 (0.61) |  |
| Preterm labor | 8 | j l m1* m2 n o q t | 24 / 347 | 0.06 (0.03, 0.11) | 44 / 577 | 0.08 (0.04, 0.15) | 0.86 (0.51, 1.43) | 0 (0.53) |  |
| Preterm labor with onset before 37w GA **^2^** | 6 | j l n o q t | 14 / 141 | 0.08 (0.02, 0.29) | 37 / 295 | 0.15 (0.06, 0.35) | 0.88 (0.51, 1.51) | 0 (0.79) |  |
| Cesarean Delivery | 12 | b g h j l m1 m2 n o q s t | 439 / 1333 | 0.37 (0.26, 0.50) | 715 / 2173 | 0.37 (0.28, 0.47) | 1.00 (0.90, 1.11) | 0 (0.70) |  |
| Intrapartum Cesarean Delivery | 8 | b j l m1* m2 n o t | 194 / 897 | 0.22 (0.16, 0.30) | 266 / 1659 | 0.17 (0.12, 0.23) | **1.35 (1.14, 1.60)** | 0 (1.00) |  |
|  |  |  |  |  |  |  |  |  |  |
| Stillbirth **^3^** | 17 | b e f g h* i j l* m1 m2* n* o p q* r s* t* | 13 / 1778 | 7.31 (4.25, 12.55) | 22 / 2961 | 6.98 (2.90, 16.68) | 1.34 (0.62, 2.90) | 0 (0.76) |  |
| Perinatal death | 12 | b e j l* m1 m2 n* o q r s* t* | 16 / 1356 | 6.52 (1.77, 13.25) | 17 / 2264 | 4.33 (1.38, 8.39) | 1.78 (0.89, 3.54) | 0 (0.91) |  |
| Early neonatal death | 12 | b e j* l* m1* m2 n* o* q r* s* t* | 5 / 1181 | 4.24 (1.77, 10.14) | 5 / 1898 | 2.98 (0.79, 11.17) | 1.60 (0.45, 5.62) | 0 (0.52) |  |
| Neonatal death **^4^** | 13 | b e i j* l* m1* m2 n* o* q r* s* t* | 5 / 1218 | 4.11 (1.71, 9.82) | 8 / 1995 | 4.18 (1.63, 10.69) | 1.25 (0.43, 3.60) | 0 (0.82) |  |
| NICU Admission at Birth | 8 | b m1 m2 n q r s t | 134 / 970 | 0.14 (0.03, 0.41) | 227 / 1546 | 0.19 (0.06, 0.43) | 1.03 (0.85, 1.25) | 0 (0.80) |  |
|  |  |  |  |  |  |  |  |  |  |
| Very low birthweight (<1500g) | 14 | b g i j* l m1 m2 n o p* q* r* s t* | 21 / 1219 | 0.01 (0.01, 0.03) | 43 / 2049 | 0.02 (0.02, 0.03) | 1.03 (0.61, 1.73) | 0 (0.93) |  |
| Low birthweight (<2500g) | 14 | b g i j l m1 m2 n o p q r s t | 165 / 1349 | 0.12 (0.09, 0.15) | 241 / 2342 | 0.09 (0.06, 0.13) | **1.27 (1.04, 1.54)** | 0 (0.73) |  |
| Small for gestational age (3rd) | 15 | b e g i j* l m1 m2 n o p q r s* t | 56 / 1728 | 0.03 (0.02, 0.06) | 48 / 2776 | 0.02 (0.01, 0.03) | **2.11 (1.42, 3.11)** | 0 (1.00) |  |
| Small for gestational age (10th) | 15 | b e g i j l m1 m2 n o p q r s t | 183 / 1766 | 0.10 (0.08, 0.13) | 175 / 2953 | 0.06 (0.04, 0.08) | **1.74 (1.41, 2.15)** | 0 (0.50) |  |
| Moderate preterm birth (<34w) | 15 | b e g i j l m1 m2 n o p q r s t | 84 / 1766 | 0.05 (0.03, 0.06) | 143 / 2953 | 0.05 (0.03, 0.06) | 1.10 (0.84, 1.44) | 0 (0.98) |  |
| Moderate preterm birth (<34w) with onset before 34w GA **^2^** | 8 | b g j n o p q t | 45 / 507 | 0.09 (0.07, 0.12) | 81 / 938 | 0.11 (0.07, 0.17) | 1.07 (0.74, 1.53) | 0 (0.98) |  |
| Preterm birth (<37 wks) | 15 | b e g i j l m1 m2 n o p q r s t | 226 / 1766 | 0.12 (0.09, 0.17) | 393 / 2953 | 0.14 (0.10, 0.19) | 1.02 (0.87, 1.19) | 0 (0.99) |  |
| Preterm birth (<37 wks) with onset before 37w GA **^2^** | 9 | b g j n o p q s t | 114 / 654 | 0.23 (0.10, 0.46) | 217 / 1225 | 0.31 (0.19, 0.46) | 1.02 (0.83, 1.26) | 0 (0.90) |  |
| *Notes: Pooled absolute risks are calculated using a logistic-normal random-effects meta-analysis, pooling all participating studies with at least 1 adverse event for the given outcome; in cases where the model does not converge, we instead calculate pooled absolute risk using a random effects model with Freeman-Tukey Double Arcsine Transformation. For any study with zero events in one arm at the study level (Risk Group or Reference Group), we apply a continuity correction of 0.5 for pooled absolute risks. For any outcome with zero events for all studies in a given risk or reference group, we consider the absolute risk to be incalculable based on the current data. Relative risks are calculated by pooling unadjusted relative risks from all participating studies with at least 1 adverse event for the given outcome using a DerSimonian-Laird random effects model meta-analysis. For any study with zero events in one arm (Risk Group or Reference Group), we used a continuity correction of the inverse of the number of events in the oppposite group within the same study. We present pooled estimates for analyses where at least three studies report the relevant data and report at least 1 adverse event for the outcome of interest.  1 This column lists the studies that provide data for the given estimate. Any study sites indicated with an asterisk (*) reported "0" events in among those with and without the given risk factor. These "0 event" studies are excluded from the estimate and from the "Events/Total" column. Studies are identified as follows: a) Martinez-Portilla, 2021, Mexico; b) Favre, Panchaud, 2021, multicountry COVI-Preg registry; c1) Money, 2020 – Maternal Subset, Canada; c2) Money, 2020 – Infant Subset, Canada; d) Carrillo, 2021, Chile; e) Knight, 2021, United Kingdom; f) Bracero, Valencia, Delgado-Lopez, 2021, Puerto Rico (USA); g) Sakowicz, 2020, USA (Chicago); h) Sanin, Mesa, Tolosa, 2021, Colombia; i) Nachega, 2021, multicountry AFREHealth study; j) Waldorf, Lokken, 2021, USA (Washington State); k) Divakar, 2021, India (Karnataka State); l) Gil, Fernandez Buhigas, 2021, Spain (Madrid); m1) Crovetto, 2020, Cohort I, Spain (Barcelona); m2) Crovetto, 2020, Cohort II, Spain (Barcelona); n) Bevilacqua, Laurita Longo, 2020, Italy (Rome); o) Nunes, 2021, South Africa; p) Akelo, Tippett Barr, 2021, Kenya; q) Yang, Juan, 2020, China; r) Kalafat, 2020, Turkey; s) Brandt, 2020, USA (New Brunswick); t) Poon, 2021, Hong Kong (China). 2 These outcomes (preterm labor, moderate preterm birth before 34 weeks gestation, and preterm birth before 37 weeks’ gestation) were included in the sensitivity analyses where we restrict confirmed COVID-19 cases to those with confirmed COVID-19 onset prior to 37 weeks’ gestation (or 34 weeks for very moderate preterm birth). The full comparison group is used for each of the sensitivity analyses.  3 The outcome presented here is stillbirths occurring at or after 28 weeks gestational age per the WHO definition.  4 The outcome "neonatal death" is reported by 15 participating studies. However, most studies were not designed to follow-up neonates until 28 days after birth. Therefore, counts of neonatal death are underestimated.* | | | | | | | | |  |
|  |  |  |  |  |  |  |  |  |  |
|  |  |  |  |  |  |  |  |  |  |
|  |  |  |  |  |  |  |  |  |  |
|  |  |  |  |  |  |  |  |  |  |
|  |  |  |  |  |  |  |  |  |  |
|  |  |  |  |  |  |  |  |  |  |
|  |  |  |  |  |  |  |  |  |  |
|  |  |  |  |  |  |  |  |  |  |
|  |  |  |  |  |  |  |  |  |  |
|  |  |  |  |  |  |  |  |  |  |
|  |  |  |  |  |  |  |  |  |  |
|  |  |  |  |  |  |  |  |  |  |
|  |  |  |  |  |  |  |  |  |  |
|  |  |  |  |  |  |  |  |  |  |
|  |  |  |  |  |  |  |  |  |  |

Table S17. Relative risk and 95% CI comparing women with each risk factor to women without risk factor - symptomatic COVID-19

| **Outcome** |  | **Symptomatic** |  |  |  |  |  |
| --- | --- | --- | --- | --- | --- | --- | --- |
|  | N | Pooled RR (95% CI) |  |  |  |  |  |
| **COVID-19 Severity & Mortality** |  |  |  |  |  |  |  |
| ICU admission | 14 | **6.52 (3.34, 12.74)** |  |  |  |  |  |
| Ventilation | 13 | 2.20 (0.81, 5.97) |  |  |  |  |  |
| Critical Care | 12 | **7.35 (3.36, 16.08)** |  |  |  |  |  |
| Pneumonia | 9 | **12.16 (2.27, 65.28)** |  |  |  |  |  |
| Pregnancy-related death | 15 | 2.88 (0.62, 13.28) |  |  |  |  |  |
|  |  |  |  |  |  |  |  |
| **Maternal Morbidity** |  |  |  |  |  |  |  |
| Haemorrhage | 6 | 0.82 (0.55, 1.22) |  |  |  |  |  |
| Placental Abruption | 5 | 0.43 (0.12, 1.61) |  |  |  |  |  |
| Preeclampsia | 13 | 1.17 (0.89, 1.54) |  |  |  |  |  |
| Preeclampsia or Eclampsia | 8 | 1.34 (0.90, 1.98) |  |  |  |  |  |
| Hypertensive Disorders of Pregnancy (Any) | 11 | 1.26 (0.99, 1.61) |  |  |  |  |  |
| Hypertensive Disorders of Pregnancy (At/After Covid-19) | 3 | -- |  |  |  |  |  |
| Preterm labor | 10 | 1.24 (0.85, 1.79) |  |  |  |  |  |
| Preterm labor with onset before 37w GA | 8 | **0.55 (0.37, 0.80)** |  |  |  |  |  |
| Cesarean Delivery | 14 | 1.08 (0.99, 1.18) |  |  |  |  |  |
| Intrapartum Cesarean Delivery | 10 | **1.25 (1.05, 1.48)** |  |  |  |  |  |
|  |  |  |  |  |  |  |  |
| **Fetal & Neonatal Mortality and Morbidity** |  |  |  |  |  |  |  |
| Stillbirth | 18 | 0.99 (0.51, 1.91) |  |  |  |  |  |
| Perinatal death | 14 | 0.92 (0.50, 1.71) |  |  |  |  |  |
| Early neonatal death | 14 | 0.73 (0.22, 2.42) |  |  |  |  |  |
| Neonatal death | 14 | 0.67 (0.25, 1.80) |  |  |  |  |  |
| NICU Admission at Birth | 9 | 1.08 (0.89, 1.30) |  |  |  |  |  |
|  |  |  |  |  |  |  |  |
| **Adverse Birth Outcomes** |  |  |  |  |  |  |  |
| Very low birthweight (<1500g) | 15 | 1.48 (0.92, 2.38) |  |  |  |  |  |
| Low birthweight (<2500g) | 15 | 1.18 (0.99, 1.40) |  |  |  |  |  |
| Small for gestational age (3rd) | 16 | 1.00 (0.71, 1.39) |  |  |  |  |  |
| Small for gestational age (10th) | 16 | 0.99 (0.79, 1.25) |  |  |  |  |  |
| Moderate preterm birth (<34w) | 16 | **1.67 (1.02, 2.74)** |  |  |  |  |  |
| Moderate preterm birth (<34w) with onset before 34w GA | 9 | **0.64 (0.44, 0.93)** |  |  |  |  |  |
| Preterm birth (<37 wks) | 16 | **1.31 (1.07, 1.62)** |  |  |  |  |  |
| Preterm birth (<37 wks) with onset before 37w GA | 10 | 0.73 (0.52, 1.03) |  |  |  |  |  |
| Notes: Relative risks are calculated by pooling unadjusted relative risks from all participating studies with at least 1 adverse event for the given outcome using a DerSimonian-Laird random effects model meta-analysis. For any study with zero events in one arm (Risk Group or Reference Group), we used a continuity correction of the inverse of the number of events in the oppposite group within the same study.  1 These outcomes (preterm labor, moderate preterm birth before 34 weeks gestation, and preterm birth before 37 weeks’ gestation) were included in the sensitivity analyses where we restrict confirmed COVID-19 cases to those with confirmed COVID-19 onset prior to 37 weeks’ gestation (or 34 weeks for very moderate preterm birth). The full comparison group is used for each of the sensitivity analyses.  2 The outcome presented here is stillbirths occurring at or after 28 weeks gestational age per the WHO definition.  3 The outcome "neonatal death" is reported by 15 participating studies. However, most studies were not designed to follow-up neonates until 28 days after birth. Therefore, counts of neonatal death are underestimated. | | | |  |  |  |  |
|  |  |  |  |  | | | |
|  |  |  |  |  | | | |
|  |  |  |  |  | | | |
|  |  |  |  |  | | | |
|  |  |  |  |  | | | |
|  |  |  |  |  | | | |

Table S18. Risk of Outcomes among COVID+ pregnancy, stratified by symptomatic/asymptomatic = ref group = asymptomatic

| Outcome | N Studies **final** | Included Studies **^1^** | Symptomatic for COVID-19 | | Asymptomatic for COVID-19 | | Pooled, unadjusted RR (95% CI) | I^2 (pvalue) |  |
| --- | --- | --- | --- | --- | --- | --- | --- | --- | --- |
|  |  |  | Events/Total | Pooled Risk (95% CI) **final** | Events/Total | Pooled Risk (95% CI) **final** |  |  |  |
|  |  |  |  |  |  |  |  |  |  |
| ICU admission | 14 | b d e g h j l* m1* m2 n q r s t | 321 / 4355 | 0.05 (0.03, 0.09) | 7 / 2293 | 0.00 (0.00, 0.01) | **6.52 (3.34, 12.74)** | 0 (0.45) |  |
| Ventilation | 13 | b d e j k l m1* m2 n q r s t | 113 / 3971 | 0.03 (0.02, 0.04) | 2 / 2286 | 0.00 (0.00, 0.01) | 2.20 (0.81, 5.97) | 0 (0.93) |  |
| Critical Care | 12 | b d e j l m1* m2 n q r s t* | 263 / 3885 | 0.05 (0.03, 0.08) | 5 / 2135 | 0.00 (0.00, 0.01) | **7.35 (3.36, 16.08)** | 3.48 (0.41) |  |
| Pneumonia | 9 | b d e l m1* m2 n r t* | 385 / 3583 | 0.17 (0.06, 0.32) | 8 / 2020 | 0.00 (0.00, 0.01) | **12.16 (2.27, 65.28)** | 57.05 (0.03) |  |
| Pregnancy-related death | 15 | b d e h j k* l* m1* m2* n* o q* r* s* t* | 45 / 2825 | 1714.01 (642.16, 4494.02) | 1 / 1688 | 0.00 (0.00, 64.21) | 2.88 (0.62, 13.28) | 0 (0.64) |  |
|  |  |  |  |  |  |  |  |  |  |
| Haemorrhage | 6 | g h j l n o | 68 / 886 | 0.06 (0.02, 0.14) | 42 / 398 | 0.10 (0.06, 0.16) | 0.82 (0.55, 1.22) | 6.78 (0.37) |  |
| Placental Abruption | 5 | g j l r* t | 4 / 589 | 0.01 (0.00, 0.02) | 4 / 261 | 0.02 (0.01, 0.04) | 0.43 (0.12, 1.61) | 0 (0.94) |  |
| Preeclampsia | 13 | b d g k l m1 m2 o p* q r s t | 130 / 2710 | 0.06 (0.04, 0.09) | 92 / 1812 | 0.05 (0.03, 0.08) | 1.17 (0.89, 1.54) | 0 (1.00) |  |
| Preeclampsia or Eclampsia | 8 | g k m1 m2 n o p* t | 66 / 713 | 0.08 (0.05, 0.13) | 45 / 646 | 0.06 (0.03, 0.10) | 1.34 (0.90, 1.98) | 0 (0.93) |  |
| Hypertensive Disorders of Pregnancy (Any) | 11 | d g k l m1 m2 n o p q t | 160 / 1496 | 0.09 (0.06, 0.13) | 106 / 1320 | 0.07 (0.04, 0.11) | 1.26 (0.99, 1.61) | 0 (0.94) |  |
| Hypertensive Disorders of Pregnancy (At/After Covid-19) | 3 | l p* t | -- | -- | -- | -- | -- | -- |  |
| Preterm labor | 10 | d j k l m1* m2 n o q t | 84 / 1196 | 0.07 (0.04, 0.12) | 46 / 1052 | 0.04 (0.02, 0.07) | 1.24 (0.85, 1.79) | 0 (0.45) |  |
| Preterm labor with onset before 37w GA **^2^** | 8 | d j k l n o q t | 74 / 783 | 0.11 (0.05, 0.24) | 38 / 285 | 0.10 (0.04, 0.25) | **0.55 (0.37, 0.80)** | 0 (0.62) |  |
| Cesarean Delivery | 14 | b d g h j k l m1 m2 n o q s t | 1094 / 2884 | 0.40 (0.31, 0.49) | 674 / 1909 | 0.34 (0.26, 0.44) | 1.08 (0.99, 1.18) | 0 (0.94) |  |
| Intrapartum Cesarean Delivery | 10 | b d j k l m1* m2 n o t | 534 / 2314 | 0.23 (0.18, 0.29) | 296 / 1536 | 0.17 (0.12, 0.25) | **1.25 (1.05, 1.48)** | 12.56 (0.33) |  |
|  |  |  |  |  |  |  |  |  |  |
| Stillbirth **^3^** | 18 | b d e f g h* j k l* m1 m2* n* o p q* r s* t* | 21 / 3328 | 6.51 (3.42, 12.33) | 16 / 2323 | 6.89 (4.22, 11.21) | 0.99 (0.51, 1.91) | 0 (0.85) |  |
| Perinatal death | 14 | b d e j k l* m1 m2 n* o q r s* t* | 26 / 2929 | 8.88 (6.05, 13.01) | 19 / 2018 | 9.42 (6.01, 14.71) | 0.92 (0.50, 1.71) | 0 (0.72) |  |
| Early neonatal death | 14 | b d e j* k* l* m1* m2 n* o* q r* s* t* | 8 / 2532 | 3.16 (1.58, 6.30) | 6 / 1654 | 3.58 (1.49, 8.59) | 0.73 (0.22, 2.42) | 0 (0.67) |  |
| Neonatal death **^4^** | 14 | b d e j* k l* m1* m2 n* o* q r* s* t* | 9 / 2598 | 3.46 (1.80, 6.64) | 10 / 1800 | 5.55 (2.99, 10.29) | 0.67 (0.25, 1.80) | 0 (0.62) |  |
| NICU Admission at Birth | 9 | b d m1 m2 n q r s t | 263 / 2121 | 0.16 (0.06, 0.36) | 137 / 1497 | 0.12 (0.04, 0.33) | 1.08 (0.89, 1.30) | 0 (0.85) |  |
|  |  |  |  |  |  |  |  |  |  |
| Very low birthweight (<1500g) | 15 | b d g j* k l m1 m2 n o p* q* r* s t* | 62 / 2622 | 0.02 (0.02, 0.03) | 29 / 1818 | 0.01 (0.01, 0.03) | 1.48 (0.92, 2.38) | 0 (0.90) |  |
| Low birthweight (<2500g) | 15 | b d g j k l m1 m2 n o p q r s t | 348 / 2918 | 0.12 (0.09, 0.15) | 190 / 1944 | 0.08 (0.06, 0.12) | 1.18 (0.99, 1.40) | 0 (0.51) |  |
| Small for gestational age (3rd) | 16 | b d e g j* k l m1 m2 n o p q r s* t | 94 / 3398 | 0.03 (0.01, 0.05) | 72 / 2283 | 0.02 (0.01, 0.05) | 1.00 (0.71, 1.39) | 0 (0.83) |  |
| Small for gestational age (10th) | 16 | b d e g j k l m1 m2 n o p q r s t | 267 / 3532 | 0.08 (0.06, 0.12) | 203 / 2364 | 0.07 (0.04, 0.11) | 0.99 (0.79, 1.25) | 14.05 (0.29) |  |
| Moderate preterm birth (<34w) | 16 | b d e g j k l m1 m2 n o p q r s t | 209 / 3532 | 0.05 (0.04, 0.07) | 69 / 2364 | 0.02 (0.01, 0.04) | **1.67 (1.02, 2.74)** | 42.34 (0.04) |  |
| Moderate preterm birth (<34w) with onset before 34w GA **^2^** | 9 | b d g j k n o p t | 130 / 1448 | 0.11 (0.07, 0.17) | 57 / 395 | 0.16 (0.10, 0.24) | **0.64 (0.44, 0.93)** | 17.02 (0.29) |  |
| Preterm birth (<37 wks) | 16 | b d e g j k l m1 m2 n o p q r s t | 550 / 3532 | 0.16 (0.12, 0.20) | 257 / 2364 | 0.10 (0.07, 0.13) | **1.31 (1.07, 1.62)** | 27.48 (0.15) |  |
| Preterm birth (<37 wks) with onset before 37w GA **^2^** | 10 | b d g j k n o p q t | 364 / 1921 | 0.30 (0.18, 0.47) | 191 / 585 | 0.37 (0.23, 0.53) | 0.73 (0.52, 1.03) | 64.48 (0.003) |  |
| *Notes: Pooled absolute risks are calculated using a logistic-normal random-effects meta-analysis, pooling all participating studies with at least 1 adverse event for the given outcome; in cases where the model does not converge, we instead calculate pooled absolute risk using a random effects model with Freeman-Tukey Double Arcsine Transformation. For any study with zero events in one arm at the study level (Risk Group or Reference Group), we apply a continuity correction of 0.5 for pooled absolute risks. For any outcome with zero events for all studies in a given risk or reference group, we consider the absolute risk to be incalculable based on the current data. Relative risks are calculated by pooling unadjusted relative risks from all participating studies with at least 1 adverse event for the given outcome using a DerSimonian-Laird random effects model meta-analysis. For any study with zero events in one arm (Risk Group or Reference Group), we used a continuity correction of the inverse of the number of events in the oppposite group within the same study. We present pooled estimates for analyses where at least three studies report the relevant data and report at least 1 adverse event for the outcome of interest.  1 This column lists the studies that provide data for the given estimate. Any study sites indicated with an asterisk (*) reported "0" events in among those with and without the given risk factor. These "0 event" studies are excluded from the estimate and from the "Events/Total" column. Studies are identified as follows: a) Martinez-Portilla, 2021, Mexico; b) Favre, Panchaud, 2021, multicountry COVI-Preg registry; c1) Money, 2020 – Maternal Subset, Canada; c2) Money, 2020 – Infant Subset, Canada; d) Carrillo, 2021, Chile; e) Knight, 2021, United Kingdom; f) Bracero, Valencia, Delgado-Lopez, 2021, Puerto Rico (USA); g) Sakowicz, 2020, USA (Chicago); h) Sanin, Mesa, Tolosa, 2021, Colombia; i) Nachega, 2021, multicountry AFREHealth study; j) Waldorf, Lokken, 2021, USA (Washington State); k) Divakar, 2021, India (Karnataka State); l) Gil, Fernandez Buhigas, 2021, Spain (Madrid); m1) Crovetto, 2020, Cohort I, Spain (Barcelona); m2) Crovetto, 2020, Cohort II, Spain (Barcelona); n) Bevilacqua, Laurita Longo, 2020, Italy (Rome); o) Nunes, 2021, South Africa; p) Akelo, Tippett Barr, 2021, Kenya; q) Yang, Juan, 2020, China; r) Kalafat, 2020, Turkey; s) Brandt, 2020, USA (New Brunswick); t) Poon, 2021, Hong Kong (China). 2 These outcomes (preterm labor, moderate preterm birth before 34 weeks gestation, and preterm birth before 37 weeks’ gestation) were included in the sensitivity analyses where we restrict confirmed COVID-19 cases to those with confirmed COVID-19 onset prior to 37 weeks’ gestation (or 34 weeks for very moderate preterm birth). The full comparison group is used for each of the sensitivity analyses.  3 The outcome presented here is stillbirths occurring at or after 28 weeks gestational age per the WHO definition.  4 The outcome "neonatal death" is reported by 15 participating studies. However, most studies were not designed to follow-up neonates until 28 days after birth. Therefore, counts of neonatal death are underestimated.* | | | | | | | | |  |
|  |  |  |  |  |  |  |  |  |  |
|  |  |  |  |  |  |  |  |  |  |
|  |  |  |  |  |  |  |  |  |  |
|  |  |  |  |  |  |  |  |  |  |
|  |  |  |  |  |  |  |  |  |  |
|  |  |  |  |  |  |  |  |  |  |
|  |  |  |  |  |  |  |  |  |  |
|  |  |  |  |  |  |  |  |  |  |
|  |  |  |  |  |  |  |  |  |  |
|  |  |  |  |  |  |  |  |  |  |
|  |  |  |  |  |  |  |  |  |  |
|  |  |  |  |  |  |  |  |  |  |
|  |  |  |  |  |  |  |  |  |  |
|  |  |  |  |  |  |  |  |  |  |
|  |  |  |  |  |  |  |  |  |  |

Table S19 Risk Factor Missingness by Site

| **Study** | **BMI** | **Diabetes (existing)** | **Hypertension (chronic)** | **CVD (existing)** | **Primapara** | **Maternal age (15-45)** | **A/Symptomatic** | **Hgb at diagnosis (<110g/L)** | **HIV** |  |
| --- | --- | --- | --- | --- | --- | --- | --- | --- | --- | --- |
| Martinez-Portilla, 2021 | 100% | 0% | 0% | 0% | 100% | 0% | 100% | 100% | 100% |  |
| Favre, Panchaud, 2021 | 100% | 0% | 0% | 0% | 0% | 0% | 0% | 71% | 34% |  |
| Money, 2020 - Maternal Subset ^1,2^ | 62% | 14% | 0% | 0% | 100% | 0-14% | 100% | 100% | 100% |  |
| Money, 2020 - Infant Subset ^1^ | 79% | 70% | 70% | 70% | 100% | 70% | 100% | 100% | 100% |  |
| Carrillo, 2021 | 21% | 0% | 0% | 0% | 100% | 1% | 0% | 100% | 100% |  |
| Knight, 2021 | 100% | 0% | 0% | 0% | 1% | 8% | 0% | 100% | 100% |  |
| Bracero, Valencia, Delgado-Lopez, 2021 | 30% | 12% | 14% | 89% | 10% | 2% | 46% | 100% | 100% |  |
| Sakowicz, 2020 | 100% | 64% | 64% | 64% | 0% | 0% | 2% | 100% | 100% |  |
| Sanin, Mesa, Tolosa, 2021 | 74% | 20% | 20% | 20% | 19% | 100% | 21% | 48% | 20% |  |
| Nachega, 2021 | 100% | 0% | 3% | 51% | 14% | 1% | 0% | 49% | 0% |  |
| Waldorf, Lokken, 2021 | 6% | 0% | 0% | 0% | 0% | 0% | 0% | 100% | 8% |  |
| Divakar, 2021 | 83% | 0% | 0% | 0% | 100% | 0% | 0% | 55% | 100% |  |
| Gil, Fernandez Buhigas, 2021 | 2% | 0% | 0% | 0% | 0% | 0% | 0% | 100% | 0% |  |
| Crovetto, 2020, Cohort II | 0% | 0% | 100% | 0% | 0% | 0% | 0% | 100% | 100% |  |
| Crovetto, 2020, Cohort I | 0% | 0% | 100% | 0% | 0% | 0% | 0% | 100% | 100% |  |
| Bevilacqua, Laurita Longo, 2020 | 10% | 0% | 0% | 0% | 1% | 0% | 0% | 100% | 100% |  |
| Nunes, 2021 | 100% | 0% | 0% | 0% | 5% | 0% | 1% | 100% | 3% |  |
| Akelo, Tippett Barr, 2021 | 100% | 9% | 9% | 100% | 1% | 0% | 3% | 100% | 100% |  |
| Yang, Juan, 2020 | 100% | 100% | 100% | 100% | 0% | 0% | 0% | 100% | 100% |  |
| Kalafat, 2020 | 100% | 0% | 0% | 0% | 0% | 0% | 0% | 100% | 0% |  |
| Brandt, 2020 | 100% | 0% | 0% | 100% | 0% | 0% | 0% | 100% | 100% |  |
| Poon, 2021 | 100% | 0% | 0% | 0% | 0% | 0% | 0% | 64% | 0% |  |
| Notes:This table shows the percentage of missing or unknown observations for each risk factor. | | | | | | | | | |  |
| 1 Note: Data from Cancovid-Preg (Money, 2020) represents all provinces, with missing data randomly distributed across provinces. Because of reporting differences based on the timing of data submission for this ongoing study, the availability of risk factors varies across infant and maternal cohorts. | | | | | | | | | |  |
|  |  |  |  |  |  |  |  |  |  |  |
| 2 Note: Data availability by outcome varies within the Cancovid-Preg study (Money, 2020) due to differences based on the timing of data submission for this ongoing study. For hypertension and maternal age, the rate of missing data for risk factors of maternal outcomes varies from 0% (ICU admission/critical care) to 14% (ventilation). | | | | | | | | | |  |
|  |  |  |  |  |  |  |  |  |  |  |

**Table S20.** Description of Follow-up by Study and Review of Missing Data by Outcome **^1^**

|  | Overall Follow-up | | Missing Data by Outcome: among all pregnancies | | | |  |
| --- | --- | --- | --- | --- | --- | --- | --- |
| **Study** | Number of pregnancies identified | Number of pregnancies with a recorded endpoint (%) | ICU admission | Ventilation | Critical care | Pneumonia |  |
| Martinez-Portilla, 2021 **^3^** | 11,031 | 0 (0%) | 0% | 0% | 0% | 0% |  |
| Favre, Panchaud, 2021 | 2,391 | 1,870 (78.2%) | 0% **^4^** | 0% **^4^** | 0% | 7% |  |
| Money, 2020 - Maternal Subset | 2,045 | n/a | 0% | 0% | 0% | -- |  |
| Money, 2020 - Infant Subset | -- | 2,626 **5** | -- | -- | -- | -- |  |
| Carrillo, 2021 | 1,347 | 1127 (83.7%) | 1% | 1% | 1% | 4% |  |
| Knight, 2021 | 1,243 | 1061 (85.4%) | 0% | 0% **^6^** | 0% | 3% |  |
| Bracero, Valencia, Delgado-Lopez, 2021 | 938 | 754 (80.4%) | 89%* | 90%* | 90%* | 90%* |  |
| Sakowicz, 2020 | 503 | 503 (100%) | 0% | -- | -- | -- |  |
| Sanin, Mesa, Tolosa, 2021 | 406 | 213 (52.5%) | 21% | -- | -- | 27% |  |
| Nachega, 2021 | 349 | 170 (48.7%) | 0% | 3% | 3% | 27% |  |
| Waldorf, Lokken, 2021 | 240 | 158 (65.8%) | 0% | 0% **^7^** | 0% | -- |  |
| Divakar, 2021 | 212 | 212 (100%) | -- | 0% | -- | -- |  |
| Gil, Fernandez Buhigas, 2021 | 212 | 172 (81.1%) **^8^** | 0% | 1% | 0% | 0% |  |
| Crovetto, 2020, Cohort II | 173 | 159 (91.9%) | 0% | 0% | 0% | 0% |  |
| Crovetto, 2020, Cohort I | 176 | 176 (100%) | 0% | 0% | 0% | 0% |  |
| Bevilacqua, Laurita Longo, 2020 | 163 | 163 (100%) | 0% | 0% | 0% | 0% |  |
| Nunes, 2021 | 139 | 133 (95.7%) | -- | -- | -- | -- |  |
| Akelo, Tippett Barr, 2021 | 125 | 93 (74.4%) **^9^** | 91%* | 92%* | 92%* | 92%* |  |
| Yang, Juan, 2020 | 116 | 100 (86.2%) | 0% | 0% | 0% | -- |  |
| Kalafat, 2020 | 77 | 74 (96.1%) | 0% | 0% | 0% | 0% |  |
| Brandt, 2020 | 61 | 61 (100%) | 0% | 0% | 0% | -- |  |
| Poon, 2021 | 25 | 25 (100%) | 0% | 0% | 0% | 0% |  |
| Notes **1** For each outcome, this table shows the percentage of the sample relevant for the given outcome where data is missing or unknown. Note that for the meta-analysis, we exclude any outcome that is more than 25% missing or unknown. These excluded outcomes are indicated with an asterisk (*). We indicate cases where outcomes are not collected for a study site using the "--" symbol. **2** The identified outcomes are sensitivity analyses, where we restrict each outcome (preterm labor, preterm birth, or moderate/very preterm birth) to those cases where Covid-19 onset is confirmed to be earlier than 37 weeks (for preterm birth or labor) or 34 weeks (for moderate/very preterm birth). If the study reports more than 25% missing data on preterm labor or preterm birth overall, these sensitivity analyses are also excluded.  **3** The Mexico National Registry (Martinez-Portilla, 2021) identifies cases of COVID-19 in pregnancy, but does not record pregnancy-specific outcomes (such as pregnancy outcome). For this site, the outcomes are restricted ot ICU admission, ventilation, critical care, pneumonia, and pregnancy-related death. Note that for pregnancy related death, we consider all pregnant people ages 15 to 45 who appear in registry (rather than restricted to completed pregnancies only, as this data is unavailable).  4 Note that the indicated outcomes for the COVI-Preg (Favre, Paunchaurd, 2021) study (ICU admission, ventilation, pregnancy related death) are only reported among those treated inpatient. We include these estimates -- with the complete denominator of patients in the study overall -- as the likelihood of patients having these outcomes without being treated inpatient is low. **5** Note that the Cancovid-Preg study (Money, 2020) Infant Subset includes only liveborn infants born to mothers with Covid-19; therefore, we consider all observations in this cohort to be completed pregnancies (100% follow-up).  **6** Note that ventilation data for the UKOSS study (Knight, 2021) is only reported among patients admitted to the ICU (approximately 5% of the sample). We include this estimate -- with the complete denominator of patients in the study overall -- as patients in the UK would not receive ventilation outside of the ICU setting.  7 Note that ventilation data for the Washington State Collaborative study (Waldorf, Lokken, 2021) is only reported among hospitalized patients (approximately 10% of the sample). We include this estimate -- with the complete denominator of patients in the study overall -- as the likelihood of patients receiving ventilation without being hospitalized is very small. **8** Note that for Gil and Fernandez Buhigas, 2021 (the Torrejon Hospital Study in Madrid, Spain) is an ongoing registry and some missing pregnancy outcomes can be accounted for by pregnant women who have been identified for the study but have not yet delivered. The percentage of total pregnancies in the study with a recorded endpoint among those with expected due dates 4 weeks or more before the date data was shared (May 4, 2021) is 100%.  **9** Note that the ANCOV Kenya study (Akelo, Tippett Barr, 2021) is an ongoing cohort study conducting population-level surveillance and some missing pregnancy outcomes can be accounted for by pregnant women who have been identified for the study but have not yet delivered. The percentage of total pregnancies in the study (including both Covid-positive and Covid-negative observations) with a recorded endpoint among those with expected due dates 4 weeks or more before the date data was shared (August 19, 2021) is 83%, with 147 observations missing pregnancy outcome out of 885. | | | | | | |  |
|  |  |  |  |  |  |  |  |
|  |  |  |  |  |  |  |  |

**Table S20.** Description of Follow-up by Study and Review of Missing Data by Outcome **^1^** (continued)

Missing Data by Outcome: among all completed pregnancies (with a recorded pregnancy endpoint)

| **Study** | Pregnancy-related death | Haemorrhage | Placental abruption | Preeclampsia | Preeclampsia or eclampsia | Hypertensive disorders of pregnancy (any) | Hypertensive disorders of pregnancy (diagnosed at/after COVID onset) | Preterm labor | Preterm labor (among those with onset <37w) **^2^** | Cesearean delivery | Intrapartum cesarean delivery |  |
| --- | --- | --- | --- | --- | --- | --- | --- | --- | --- | --- | --- | --- |
| Martinez-Portilla, 2021 **^3^** | 0% | -- | -- | -- | -- | -- | -- | -- | -- | -- | -- |  |
| Favre, Panchaud, 2021 | 0% **^4^** | -- | -- | 0% | -- | -- | -- | -- | -- | 2% | 2% |  |
| Money, 2020 - Maternal Subset | -- | -- | -- | -- | -- | -- | -- | -- | -- | -- | -- |  |
| Money, 2020 - Infant Subset | -- | -- | -- | -- | -- | -- | -- | -- | -- | -- | -- |  |
| Carrillo, 2021 | 1% | -- | -- | 0% | -- | 0% | -- | 0% | 0% | 1% | 1% |  |
| Knight, 2021 | 0% | -- | -- | -- | -- | -- | -- | -- | -- | -- | -- |  |
| Bracero, Valencia, Delgado-Lopez, 2021 | 25%* | -- | -- | -- | -- | -- | -- | -- | -- | -- | -- |  |
| Sakowicz, 2020 | -- | 0% | 0% | 0% | 0% | 0% | -- | -- | -- | 0% | -- |  |
| Sanin, Mesa, Tolosa, 2021 | 25%* | 0% | -- | -- | 27%* | 27%* | -- | 28.%* | -- | 23% | -- |  |
| Nachega, 2021 | 0% | 7% | 6% | -- | 5% | 3% | -- | 33%* | -- | 26%* | 26%* |  |
| Waldorf, Lokken, 2021 | 0% | 6% | 2% | -- | -- | -- | -- | 2% | 3% | 0% | 0% |  |
| Divakar, 2021 | 0% | -- | -- | 0% | 0% | 0% | -- | 0% | 0% | 9% | 9% |  |
| Gil, Fernandez Buhigas, 2021 | 0% | 0% | 0% | 0% | -- | 0% | 0% | 3% | 3% | 2% | 2% |  |
| Crovetto, 2020, Cohort II | 0% | -- | -- | 0% | 0% | 0% | -- | 3% | -- | 0% | 0% |  |
| Crovetto, 2020, Cohort I | 0% | -- | -- | 0% | 0% | 0% | -- | 0% | 0% | 0% | 0% |  |
| Bevilacqua, Laurita Longo, 2020 | 0% | 0% | -- | -- | 0% | 0% | -- | 0% | 0% | 3% | 3% |  |
| Nunes, 2021 | 1% | 0% | -- | 0% | 0% | 0% | -- | 0% | 0% | 1% | 1% |  |
| Akelo, Tippett Barr, 2021 | -- | -- | -- | 0% | 0% | 0% | 0% | -- | -- | -- | -- |  |
| Yang, Juan, 2020 | 0% | -- | -- | 0% | -- | 0% | -- | 0% | 0% | 0% | -- |  |
| Kalafat, 2020 | 0% | -- | 0% | 0% | -- | -- | -- | -- | -- | -- | -- |  |
| Brandt, 2020 | 0% | -- | -- | 0% | -- | -- | -- | -- | -- | 0% | -- |  |
| Poon, 2021 | 0% | -- | 0% | 0% | 0% | 0% | 0% | 0% | 0% | 0% | 0% |  |
| Notes: |  |  |  |  |  |  |  |  |  |  |  |  |
| **1** For each outcome, this table shows the percentage of the sample relevant for the given outcome where data is missing or unknown. Note that for the meta-analysis, we exclude any outcome that is more than 25% missing or unknown. These excluded outcomes are indicated with an asterisk (*). We indicate cases where outcomes are not collected for a study site using the "--" symbol.  **2.** The identified outcomes are sensitivity analyses, where we restrict each outcome (preterm labor, preterm birth, or moderate/very preterm birth) to those cases where Covid-19 onset is confirmed to be earlier than 37 weeks (for preterm birth or labor) or 34 weeks (for moderate/very preterm birth). If the study reports more than 25% missing data on preterm labor or preterm birth overall, these sensitivity analyses are also excluded.  **3** The Mexico National Registry (Martinez-Portilla, 2021) identifies cases of COVID-19 in pregnancy, but does not record pregnancy-specific outcomes (such as pregnancy outcome). For this site, the outcomes are restricted ot ICU admission, ventilation, critical care, pneumonia, and pregnancy-related death. Note that for pregnancy related death, we consider all pregnant people ages 15 to 45 who appear in registry (rather than restricted to completed pregnancies only, as this data is unavailable).  4 Note that the indicated outcomes for the COVI-Preg (Favre, Paunchaurd, 2021) study (ICU admission, ventilation, pregnancy related death) are only reported among those treated inpatient. We include these estimates -- with the complete denominator of patients in the study overall -- as the likelihood of patients having these outcomes without being treated inpatient is low. **5** Note that the Cancovid-Preg study (Money, 2020) Infant Subset includes only liveborn infants born to mothers with Covid-19; therefore, we consider all observations in this cohort to be completed pregnancies (100% follow-up).  **6** Note that ventilation data for the UKOSS study (Knight, 2021) is only reported among patients admitted to the ICU (approximately 5% of the sample). We include this estimate -- with the complete denominator of patients in the study overall -- as patients in the UK would not receive ventilation outside of the ICU setting.  7 Note that ventilation data for the Washington State Collaborative study (Waldorf, Lokken, 2021) is only reported among hospitalized patients (approximately 10% of the sample). We include this estimate -- with the complete denominator of patients in the study overall -- as the likelihood of patients receiving ventilation without being hospitalized is very small. **8** Note that for Gil and Fernandez Buhigas, 2021 (the Torrejon Hospital Study in Madrid, Spain) is an ongoing registry and some missing pregnancy outcomes can be accounted for by pregnant women who have been identified for the study but have not yet delivered. The percentage of total pregnancies in the study with a recorded endpoint among those with expected due dates 4 weeks or more before the date data was shared (May 4, 2021) is 100%.  **9** Note that the ANCOV Kenya study (Akelo, Tippett Barr, 2021) is an ongoing cohort study conducting population-level surveillance and some missing pregnancy outcomes can be accounted for by pregnant women who have been identified for the study but have not yet delivered. The percentage of total pregnancies in the study (including both Covid-positive and Covid-negative observations) with a recorded endpoint among those with expected due dates 4 weeks or more before the date data was shared (August 19, 2021) is 83%, with 147 observations missing pregnancy outcome out of 885. | | | | | | | | | | | |  |
|  |  |  |  |  |  |  |  |  |  |  |  |  |
|  |  |  |  |  |  |  |  |  |  |  |  |  |
|  |  |  |  |  |  |  |  |  |  |  |  |  |

**Table S20.** Description of Follow-up by Study and Review of Missing Data by Outcome **^1^** (continued)

|  | Missing Data by Outcome: among all births or livebirths (with a recorded pregnancy endpoint) | | | | | | | | | |  |
| --- | --- | --- | --- | --- | --- | --- | --- | --- | --- | --- | --- |
| **Study** | Stillbirth (at/after 28w) | Perinatal death | Early neonatal death | Neonatal death | NICU admission | Low birthweight | SGA (3rd or 10th) | Preterm birth (<37w) | Moderate or very preterm (among those with onset <34w) **^2^** | Preterm birth (among those with onset <37w) **^2^** |  |
| Martinez-Portilla, 2021 **^3^** | -- | -- | -- | -- | -- | -- | -- | -- | -- | -- |  |
| Favre, Panchaud, 2021 | 0% | 4% | 4% | 4% | 3% | 3% | 8% | 0% | 0% | 0% |  |
| Money, 2020 - Maternal Subset | -- | -- | -- | -- | -- | -- | -- | -- | -- | -- |  |
| Money, 2020 - Infant Subset | -- | -- | -- | -- | -- | -- | -- | 0% | -- | -- |  |
| Carrillo, 2021 | 0% | 0% | 0% | 0% | 11% | 2% | 9% | 1% | 0% | 0% |  |
| Knight, 2021 | 0% | 2% | 2% | 2% | -- | -- | 2% | 0% | -- | -- |  |
| Bracero, Valencia, Delgado-Lopez, 2021 | 0% | 33%* | 33%* | 32%* | -- | 30%* | 53%* | 25%* | -- | -- |  |
| Sakowicz, 2020 | 0% | -- | -- | 68%* | 68%* | 0% | 0% | 0% | 0% | 0% |  |
| Sanin, Mesa, Tolosa, 2021 | 0% | 33%* | 33%* | 33%* | 32%* | 28.%* | 29.%* | 27%* | -- | -- |  |
| Nachega, 2021 | 2% | -- | -- | 0% | -- | 13% | 18% | 12% | -- | -- |  |
| Waldorf, Lokken, 2021 | 0% | 0% | 0% | 0% | -- | 1% | 1% | 0% | 0% | 0% |  |
| Divakar, 2021 | 0% | 0% | 0% | 0% | -- | 0% | 1% | 0% | 0% | 0% |  |
| Gil, Fernandez Buhigas, 2021 | 0% | 1% | 1% | 1% | -- | 1% | 2% | 0% | 0% | 0% |  |
| Crovetto, 2020, Cohort II | 0% | 0% | 0% | 0% | 10% | 2% | 4% | 1% | -- | -- |  |
| Crovetto, 2020, Cohort I | 0% | 0% | 0% | 0% | 5% | 0% | 0% | 0% | 0% | 0% |  |
| Bevilacqua, Laurita Longo, 2020 | 0% | 0% | 0% | 0% | 1% | 1% | 1% | 0% | 0% | 0% |  |
| Nunes, 2021 | 0% | 0% | 0% | 0% | -- | 2% | 4% | 1% | 0% | 0% |  |
| Akelo, Tippett Barr, 2021 | 0% | 28.%* | 29.%* | 29.%* | -- | 7% | 19% | 7% | 0% | 0% |  |
| Yang, Juan, 2020 | 0% | 0% | 0% | 0% | 1% | 2% | 2% | 0% | 0% | 0% |  |
| Kalafat, 2020 | 0% | 1% | 1% | 1% | 1% | 0% | 0% | 0% | -- | -- |  |
| Brandt, 2020 | 0% | 0% | 0% | 0% | 0% | 0% | 0% | 0% | 0% | 0% |  |
| Poon, 2021 | 0% | 0% | 0% | 0% | 0% | 0% | 0% | 0% | 0% | 0% |  |
| Notes: |  |  |  |  |  |  |  |  |  |  |  |
| **1** For each outcome, this table shows the percentage of the sample relevant for the given outcome where data is missing or unknown. Note that for the meta-analysis, we exclude any outcome that is more than 25% missing or unknown. These excluded outcomes are indicated with an asterisk (*). We indicate cases where outcomes are not collected for a study site using the "--" symbol.  **2.** The identified outcomes are sensitivity analyses, where we restrict each outcome (preterm labor, preterm birth, or moderate/very preterm birth) to those cases where Covid-19 onset is confirmed to be earlier than 37 weeks (for preterm birth or labor) or 34 weeks (for moderate/very preterm birth). If the study reports more than 25% missing data on preterm labor or preterm birth overall, these sensitivity analyses are also excluded.  **3** The Mexico National Registry (Martinez-Portilla, 2021) identifies cases of COVID-19 in pregnancy, but does not record pregnancy-specific outcomes (such as pregnancy outcome). For this site, the outcomes are restricted ot ICU admission, ventilation, critical care, pneumonia, and pregnancy-related death. Note that for pregnancy related death, we consider all pregnant people ages 15 to 45 who appear in registry (rather than restricted to completed pregnancies only, as this data is unavailable).  4 Note that the indicated outcomes for the COVI-Preg (Favre, Paunchaurd, 2021) study (ICU admission, ventilation, pregnancy related death) are only reported among those treated inpatient. We include these estimates -- with the complete denominator of patients in the study overall -- as the likelihood of patients having these outcomes without being treated inpatient is low. **5** Note that the Cancovid-Preg study (Money, 2020) Infant Subset includes only liveborn infants born to mothers with Covid-19; therefore, we consider all observations in this cohort to be completed pregnancies (100% follow-up).  **6** Note that ventilation data for the UKOSS study (Knight, 2021) is only reported among patients admitted to the ICU (approximately 5% of the sample). We include this estimate -- with the complete denominator of patients in the study overall -- as patients in the UK would not receive ventilation outside of the ICU setting.  7 Note that ventilation data for the Washington State Collaborative study (Waldorf, Lokken, 2021) is only reported among hospitalized patients (approximately 10% of the sample). We include this estimate -- with the complete denominator of patients in the study overall -- as the likelihood of patients receiving ventilation without being hospitalized is very small. **8** Note that for Gil and Fernandez Buhigas, 2021 (the Torrejon Hospital Study in Madrid, Spain) is an ongoing registry and some missing pregnancy outcomes can be accounted for by pregnant women who have been identified for the study but have not yet delivered. The percentage of total pregnancies in the study with a recorded endpoint among those with expected due dates 4 weeks or more before the date data was shared (May 4, 2021) is 100%.  **9** Note that the ANCOV Kenya study (Akelo, Tippett Barr, 2021) is an ongoing cohort study conducting population-level surveillance and some missing pregnancy outcomes can be accounted for by pregnant women who have been identified for the study but have not yet delivered. The percentage of total pregnancies in the study (including both Covid-positive and Covid-negative observations) with a recorded endpoint among those with expected due dates 4 weeks or more before the date data was shared (August 19, 2021) is 83%, with 147 observations missing pregnancy outcome out of 885. | | | | | | | | | | |  |
|  |  |  |  |  |  |  |  |  |  |  |  |
|  |  |  |  |  |  |  |  |  |  |  |  |
